# Molecular basis of mood and cognitive adverse events elucidated via a combination of pharmacovigilance data mining and functional enrichment analysis

**DOI:** 10.1007/s00204-020-02788-1

**Published:** 2020-06-05

**Authors:** Christos Andronis, João Pedro Silva, Eftychia Lekka, Vassilis Virvilis, Helena Carmo, Konstantina Bampali, Margot Ernst, Yang Hu, Irena Loryan, Jacques Richard, Félix Carvalho, Miroslav M. Savić

**Affiliations:** 1 Biovista, 34 Rodopoleos Street, 16777 Athens, Greece; 2 UCIBIO, REQUIMTE, Laboratory of Toxicology, Department of Biological Sciences, Faculty of Pharmacy, University of Porto, 4050-313 Porto, Portugal; 3 Department of Molecular Neurosciences, Medical University of Vienna, Spitalgasse 4, 1090 Vienna, Austria; 4 Translational PKPD Group, Department of Pharmaceutical Biosciences, Associate Member of SciLifeLab, Uppsala University, Uppsala, Sweden; 5 Sanofi R&D, 371 avenue Professeur Blayac, 34000 Montpellier, France; 6 Department of Pharmacology, Faculty of Pharmacy, University of Belgrade, Vojvode Stepe 450, 11000 Belgrade, Serbia

**Keywords:** Neurotoxicity, Pharmaceuticals’ safety, Psychiatric/psychological adverse events, Cross-talk analysis, Adverse outcome pathways

## Abstract

Drug-induced Mood- and Cognition-related adverse events (MCAEs) are often only detected during the clinical trial phases of drug development, or even after marketing, thus posing a major safety concern and a challenge for both pharmaceutical companies and clinicians. To fill some gaps in the understanding and elucidate potential biological mechanisms of action frequently associated with MCAEs, we present a unique workflow linking observational population data with the available knowledge at molecular, cellular, and psychopharmacology levels. It is based on statistical analysis of pharmacovigilance reports and subsequent signaling pathway analyses, followed by evidence-based expert manual curation of the outcomes. Our analysis: (a) ranked pharmaceuticals with high occurrence of such adverse events (AEs), based on disproportionality analysis of the FDA Adverse Event Reporting System (FAERS) database, and (b) identified 120 associated genes and common pathway nodes possibly underlying MCAEs. Nearly two-thirds of the identified genes were related to immune modulation, which supports the critical involvement of immune cells and their responses in the regulation of the central nervous system function. This finding also means that pharmaceuticals with a negligible central nervous system exposure may induce MCAEs through dysregulation of the peripheral immune system. Knowledge gained through this workflow unravels putative hallmark biological targets and mediators of drug-induced mood and cognitive disorders that need to be further assessed and validated in experimental models. Thereafter, they can be used to substantially improve in silico/in vitro/in vivo tools for predicting these adversities at a preclinical stage.

**Electronic supplementary material:**

The online version of this article (10.1007/s00204-020-02788-1) contains supplementary material, which is available to authorized users.

## Introduction

Serious safety concerns contribute to a significant proportion of clinical trial failures, thus heavily increasing the costs associated with drug development (Allison 2012). Although drug safety is addressed throughout every preclinical and clinical trial phase, adverse events (AEs) may only become directly attributable to a drug as late as in phase III and post-approval (phase IV) stages (Crowther 2013). Some AEs may also occur following compassionate use of drugs. For example, the United States’ Food and Drug Administration has recently authorized the use of methylenedioxymethamphetamine (MDMA)-assisted psychotherapy for post-traumatic stress disorder (PTSD), although MDMA may induce suicidal ideation and behavior (Sessa et al. 2019). Such AEs, which show a likely causality to a drug, are known in clinical practice as adverse reactions or adverse effects (Edwards and Aronson 2000).

Within the scope of drug development, the Central Nervous System (CNS) is responsible for a lower percentage of project terminations due to safety concerns at preclinical when compared to clinical stages, implicating that certain serious CNS-related AEs are hardly predictable in the preclinical phase (Cook et al. 2014). Various drug classes are capable of inducing CNS-related AEs, in particular Mood and/or Cognition AEs (MCAEs) (Afzal et al. 2017). As a couple of illustrative examples, interferon-based immunotherapy has been shown to induce depression and suicidal ideation in patients with hepatitis C (Renault et al. 1987), cancer (Valentine et al. 1998), or multiple sclerosis (Fragoso et al. 2010). Furthermore, a recent cohort study performed in Danish women with no previous psychiatric diagnoses identified a positive association of hormonal contraception with suicidal ideation and attempt (Skovlund et al. 2018). Antiepileptic drugs such as perampanel have also been associated with adverse mood changes (e.g. depression) and effects on cognition (Afzal et al. 2017; Goji and Kanemoto 2019). In patients with lower urinary tract symptoms (Muderrisoglu et al. 2019), muscarinic antagonists and 5-alpha-reductase inhibitors were associated with impaired cognition and depression, respectively. Recently, both the United States and European regulators issued a warning about the potential of neuropsychiatric AEs of fluoroquinolones, including suicidal ideation (Bennett et al. 2019).

These AEs were largely revealed by PharmacoEpidemiology (PE) and PharmacoVigilance (PV) data. Such databases include the FDA Adverse Event Reporting System (FAERS) database (FDA 2018) that retrieves information on adverse event and medication error reports submitted to the FDA; the Global Individual Case Safety (ICSRs) Reports Database System (VigiBase) (Lindquist 2008), that collects reports of suspected adverse effects of medicines worldwide, and Eudravigilance (Postigo et al. 2018), the European Union data processing network and management system for reporting and evaluation of suspected adverse drug reactions.

As a concept complementary to PV databases, Adverse Outcome Pathways (AOPs) help to understand the mechanisms leading to adverse outcomes (AOs). In essence, AOPs describe how the interaction of a substance with a biological/toxicological target, also termed Molecular Initiating Event (MIE), triggers a sequential cascade of linked events (Key Events-KEs), at molecular, cellular or tissue levels, ultimately resulting in an AO in the organism (Bal-Price et al. 2017; Bal-Price and Meek 2017). Validated AOPs become valuable prediction tools to estimate a drug’s potential to elicit an AO (Pletz et al. 2018).

This work comprises a comprehensive joint effort led by the NeuroDeRisk consortium, formed through the Innovative Medicines Initiative (IMI) to collect, organize, and expand existing knowledge on the biological pathway elements implicated in the onset of MCAEs, with a particular focus on the role of immune system modulation. Here, we employed an original methodology with a unique workflow that represents, to the best of our knowledge, the first attempt to provide a new mechanistic interpretation of neurotoxic effects involved in mood and cognition. Contrasting with the more traditional approaches (e.g. systematic reviews, meta-analysis of clinical trials), we mined the selected PV database (FAERS) for pharmaceuticals that demonstrate a high occurrence of MCAEs based on disproportionality analysis. We then performed a functional enrichment and cross-talk analysis with the aim to elucidate the molecular mechanisms underlying these drug-induced changes. Our integrated approach has particularly highlighted the key role played by immune function mediators in the mechanisms leading to MCAEs. At the same time, our parallel top-down and bottom-up analysis revealed distinct differences both in the sets of drugs and in the biological pathways associated with MCAEs.

## Methods

### Data sources

FAERS is a publicly available database that contains Adverse Event reports that were submitted to FDA by healthcare professionals and patients (https://www.fda.gov) (FDA 2018). As of this writing, it contains over 9 million reports from 2004 to date. Individual reports in FAERS are patient-based. Reports document the patient-experienced AEs as a result of the administration of one or more drugs. Each report lists the drugs taken, with information regarding the putative role of the drug in the AEs of the report, i.e. whether a drug has been a suspect for an AE or is simply a concomitant drug. However, there is no direct association between each AE and respective drug(s) in a report. Other information contained in FAERS reports include the age of the patient and the indication for which the drug has been prescribed. FAERS data (4th quarter of 2018) were obtained using the openFDA drug adverse event Application Programming Interface (API, https://open.fda.gov/apis/downloads/).

The drugs of interest were used as queries to collect data from DGIdb (Drug Gene Interaction Database) (Cotto et al. 2018). Medical Dictionary for Regulatory Activities (MedDRA) classes corresponding to mood and cognitive AEs, Anatomical Therapeutic Chemical (ATC) codes mapping drugs to therapeutic classes and MeSH pharmacological actions, were taken from the NIH UMLS (Unified Medical Language System) version 2018AB database (Bodenreider 2004). Bibliographic links to Drugs (e.g. from Genes, Pathways, Physiology terms, etc.) were taken from Biovista’s COSS (Clinical Outcome Search Space) database, specifically using the Vizit tool (Andronis et al. 2012). Biovista Vizit is a visual bibliographic PubMed-based search tool that helps researchers and medical practitioners explore the existing knowledge of a biomedical domain, discover non-obvious links between entities (e.g. genes, pathways, drugs, etc.), visualize biological interconnections and communicate findings with peers (https://www.biovista.com/vizit/). Data on immune system genes were obtained from two publicly available databases: a) InnateDB (Breuer et al. 2013) developed by the Department of Molecular Biology and Biochemistry, Simon Fraser University, Canada, and b) The Immunology Database and Analysis Portal (ImmPort), developed by the National Institutes of Health (NIH), National Institute of Allergy and Infectious Diseases (NIAID), Division of Allergy, Immunology, and Transplantation (DAIT) (Bhattacharya et al. 2018).

### FAERS drug entries / Drug name unification

A selection of fields encompassing Indications, AEs, Patient age, Date received and Adverse Drug Reaction outcome, were used to populate an internal database.

AE names in FAERS are standardized on the MedDRA terminology. On the other hand, drug names entered in FAERS are not based on any standard terminology. Drug entries in FAERS may contain any of the following variations of terms: Drug names appended with the pharmaceutical salt, Active ingredients, Brand names, Drug combinations (usually, but not always, separated by ‘;’). Drug names also frequently contain typographic errors, making any aggregation attempt quite difficult. Finally, drugs appearing in FAERS also include Nutritional supplements used in alternative therapies and Investigational compounds. Lack of data standardization in drug naming in FAERS is a known issue and efforts to tame this problem have been carried out in the past (Banda et al. 2016; Maciejewski et al. 2017). An example drug presented with this issue is isotretinoin which exists in FAERS with at least 4 synonyms (number of rows in FAERS database in parenthesis): Accutane (10 038), isotretinoin (8 832), Roaccutane (894), Roacutan (240). Clearly, any effort to find a meaningful Drug-AEs combination would have to address drug name synonymization.

**Fig. 1:**
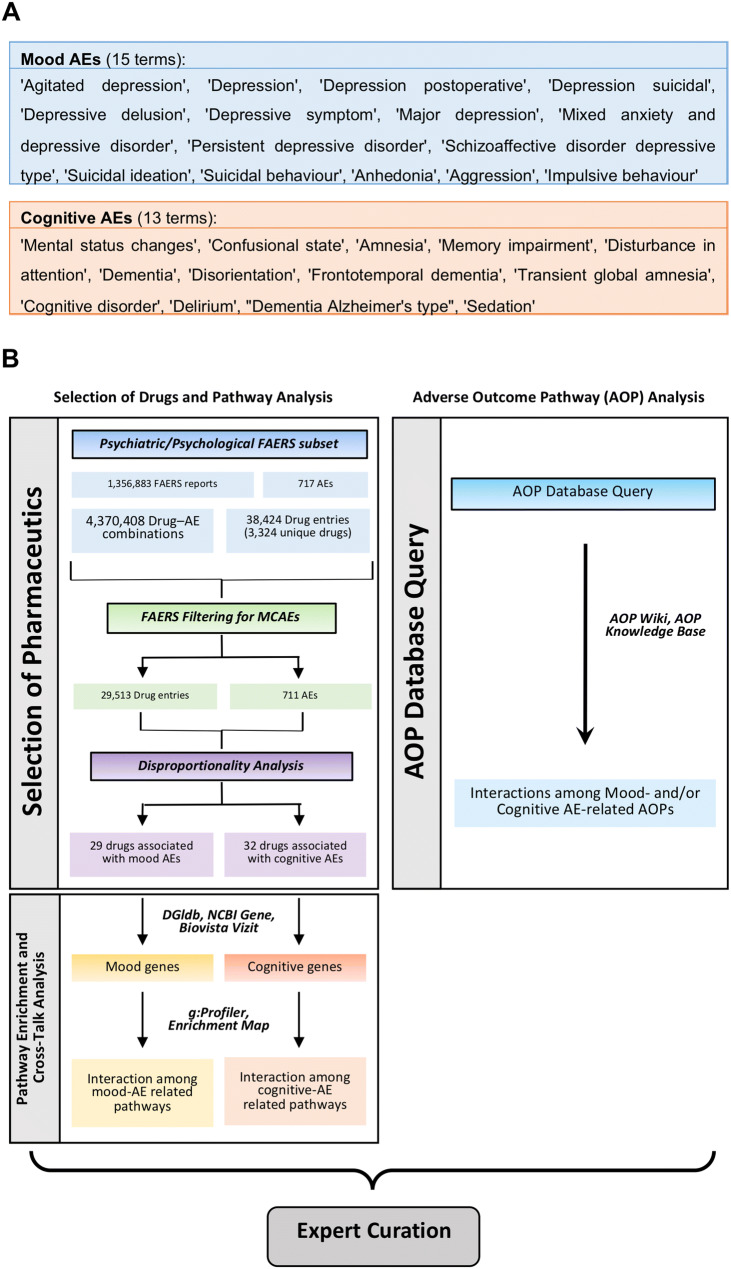
Major elements of the workflow applied to the analysis of pharmacovigilance reports and its subsequent linking with the basic biological knowledge. **a** Mood and cognitive MedDRA terms used for the selection of mood- and cognitive-related AEs. **b** Graphical representation of the two approaches used to identify the pathways and mechanisms of action leading to Mood and Cognitive AEs. Expert curation was carried out using PubMed and the Biovista Vizit tool

The initial P/P subset of AEs contained 4,370,408 Drug-AE combinations, 1,356,883 reports, 38,424 Drug entries and 717 AEs. As a result of the synonymization process, drug names were normalized to 88.4% of the reports in the P/P subset of FAERS based on their canonical form (usually the active ingredient of the drug) (Fig. 1b). More specifically, out of 38,424 drug entries in the P/P subset of FAERS, 12,235 were normalized to 3,324 drugs. 26,189 drug entries could not be further mapped to canonical names of drugs. However, these 26,189 drug entries cover only 11.6% of the reports in this subset of FAERS. All in all, 92.6% of actual Drug-AE combinations have been covered by our Drug name synonymization efforts. The P/P subset of FAERS remaining after filtering contained 1,211,954 reports, 29,513 Drug entries and 711 AEs (Fig. 1b).

### Mode of action

The ATC ontology, used as a primary means of identifying compounds for selection, based on their mode of action, was also derived from the UMLS 2018AB distribution.

### Search strategy and selection criteria

#### Terms related to MCAEs

The list of MCAEs was created by selecting Mood- and Cognition-relevant MedDRA terms from Unified Medical Language System (UMLS), as presented in Fig. 1a. Noteworthy, MedDRA uses clinically validated, standardized medical terminology, which is commonly used by regulatory authorities.

#### Selection of pharmaceuticals related to MCAEs

Drug-MCAE combinations were generated from the FAERS database (FDA Adverse Event Reporting System, https://www.fda.gov) (FDA 2018). A subset of this database was then filtered with AEs that were descendants of the MedDRA Psychiatric/Psychological (P/P) disorders class. Drug names in FAERS (as opposed to AEs) are not based on a controlled vocabulary. This requires significant consolidation and normalization before they can be used in any downstream analysis such as the one presented here. To address the problem of drug name incoherence in FAERS we employed Biovista’s internal COSS database (Deftereos et al. 2011) that contains synonyms for medicines based on a multitude of knowledge sources, including FDA approved drugs, MeSH Chemicals and Drugs, RxNorm and internal curation efforts.

Drug-MCAEs combinations in the P/P subset of FAERS were further filtered based on a number of criteria. First, Drug-AEs combinations where the Drug entry was not considered as “Suspect” in FAERS for a particular AE, were discarded. Drug-MCAEs combinations where the name of the AE was the same with the name of the indication were also discarded as they were considered as a data entry error (Maciejewski et al. 2017). Other FAERS entries (i.e. Drug-AEs combinations) with errors due to data entry (e.g. abnormal patient ages, duplicated content, etc.) were also discarded.

Drug-MCAE combinations for patients over 90 years of age were not included for further analysis not only due to their very small contribution to the overall number of reports but also because they included invalid age entries (data not shown). Similarly, Drugs-MCAEs combinations from children up to 12 years of age were not considered for further analysis due to the highly variable and unreliable AE assessment in these patients. Finally, FAERS contains numerous records (> 38.3% of the P/P reports) without patient age information. These rows were still kept for further analysis due to their significant contribution to the P/P subset.

#### Disproportionality analysis and information component methods

Disproportionality methods and Information Component (IC) (Lindquist et al. 2000) analyses are standard approaches of identifying statistical associations between Drugs and AEs reported in PV databases (such as FAERS) (Duggirala et al. 2018). In this respect, we sought to calculate the Proportional Reporting Ratio (PRR) (Bate and Evans 2009; Evans et al. 2001) and the IC in order to calculate the association between the Drugs in the P/P FAERS subset and Mood/Cognitive MCAEs.

Two separate two-by-two contingency tables were constructed, one for Mood AEs and one for Cognitive AEs, using the MedDRA AEs from Fig. 1a (Duggirala et al. 2018; Montastruc et al. 2011; Sakaeda et al. 2013).

Two-by-two contingency table used for Disproportionality analysis for a Drug X against the combination of Mood or Cognitive AEs.

**Table:** 

	M/C AE	All other AEs	
Drug X	a	b	a + b
All other products	c	d	c + d
	a + c	b + d	Total

The PRR was then calculated as follows:

1 $${\text{PRR}}\, = \,\left[ {{\text{a}}/\left( {{\text{a}}\, + \,{\text{b}}} \right)\left] {{\text{ }}/{\text{ }}} \right[{\text{c}}/\left( {{\text{c}}\, + \,{\text{d}}} \right)} \right]$$

The Information Component was calculated based on the methodology outlined by the Uppsala Monitoring Centre in https://www.who-umc.org/vigibase/vigilyze/analytics-in-vigilyze/:

2 $${\text{IC}}\, = \,{\text{log2}}\left( {\left( {{\text{Nobserved}}\, + \,0.{\text{5}}} \right)/\left( {{\text{Nexpected}}\, + \,0.{\text{5}}} \right)} \right)$$

where Nexpected = (Nsubst * Nreaction) / Ntotal.

Nexpected: the number of reports expected for the drug-AE combination.

Nobserved: the actual number of reports for the drug-AE combination.

Nsubst: the number of reports for the drug.

Nreaction: the number of reports for the effect.

### Assessment of pharmaceuticals’ targets associated with MCAEs

Biological (macro)molecules with which the pharmaceuticals interact directly (Online Resource 1), identified via the Drug Gene Interaction Database (DGIdb), were defined as candidate pathway elements (CPEs). The term “pharmaceutical(s)” herein employed comprises both small molecules and biologics. Small molecules comprise low molecular weight compounds of natural or synthetic origin that bind, and alter the function of biomolecules (e.g. proteins), thus regulating biological processes. In contrast, biologics account for molecules of biological origin, with high molecular weight (e.g. monoclonal antibodies, proteins), most of which do not readily cross cell membranes but may trigger messenger responses (Samanen 2013). The nature of association of each CPE with MCAEs was further validated in PubMed, either directly or with the help of the Biovista Vizit tool, by cross-referencing CPEs with the MCAE terms (Fig. 1a). Only targets clearly reported to have a direct association with the above-mentioned AEs were further listed in Online Resource 2. The following information was added to each CPE listed in Online Resource 2: name of the gene encoding for the CPE; name of the pathways in which the CPE participates, according to Reactome (https://reactome.org) and WikiPathways (https://www.wikipathways.org); citation(s) of the publications comprising the rationale for retaining the CPE in the curated list; sites of target expression, assessed using “The Human Protein Atlas” and “Human Protein Reference Database” databases, divided by level of protein expression whenever applicable; pharmaceuticals associated with the CPE, according to DGIdb.

### Pathway functional enrichment and cross-talk analysis

Genes encoding for the identified CPEs were mapped to known functional information in humans, using the g:GOSt functionality of g:Profiler (https://biit.cs.ut.ee/gprofiler/gost) (Raudvere et al. 2019), based on Reactome biological pathways. Significantly enriched pathways were identified. Size of functional categories were limited to minimum 3 and maximum 350. Small pathways decrease the statistical power because of excessive multiple testing while very large pathways are of very generic nature. Moreover, the intersection query/term was set to 3 in order to consider only more reliable pathways. Pathways were automatically ranked according to their adjusted p-value (statistical significance). Genes, from the list provided, associated with the respective pathways were also indicated. The interconnections of enriched Reactome pathways were visualized using the EnrichmentMap plugin from within Cytoscape (https://www.baderlab.org/Software/EnrichmentMap) (Merico et al. 2010).

### Adverse outcome pathways related to MCAEs

The published AOPs were also considered for the assessment of pathways directly associated with Mood and Cognition-related changes. AOPWiki (AOP-Wiki 2019) and AOP-Knowledge Base (AOP-KB 2019) were queried using the broad terms associated with Mood and Cognition AEs listed in Fig. 1a. The association of the query-retrieved AOPs with MCAEs was manually curated by subject experts.

### Identification of CPEs associated with immune system-related MoA

Identification of CPEs associated with an immune function response was performed by querying InnateDB and ImmPort databases for all immune system-related genes, followed by their mapping in the “Interacting Genes” list (taken from DGIdb). Further manual curation of the CPEs encoded by those genes, as well as cross-referencing of CPEs listed in Online Resource 2 that were not retrieved by InnateDB or ImmPort, was performed by carefully reading the publications retrieved by the PubMed database.

## Results

### Outline of an integrative approach to AE identification

An illustration of the integrative approach proposed by our group is provided in Fig. 1b. For the Pathway Enrichment Analysis, lists of MCAEs (MedDRA terms in Fig. 1a) were used to create a FAERS-based resource of drugs associated with either of the two categories of AEs. Drugs were ranked according to the number of reports containing such AEs, based on disproportionality analysis. Drug-gene interaction databases and tools (DGIdb, NCBI Gene and Biovista Vizit) were used to identify interacting genes and CPEs for each drug. CPEs with an immunomodulatory mechanism of action were retrieved from immunological databases (Immport, Innate DB). Pathway Enrichment Analysis (g:Profiler, Enrichment Map) resulted in enriched pathway networks associated with Mood and/or Cognition AEs. The same lists of MCAEs (Fig. 1a) were used to query AOP databases (AOP Wiki and AOP Knowledge Base) in order to identify AOPs involving these adverse events. MoA information was subsequently curated in PubMed for establishing supposedly causal rather than coincidental relationships between a CPE and each MCAE.

### Identification of pharmaceuticals associated with MCAEs in FAERS

To establish Drug-MCAEs associations we employed a disproportionality analysis. The PRR and IC values with CI 95% were calculated to establish a causative relationship between a pharmaceutical and MCAEs. A pharmaceutical was considered positively correlated with a MCAE if it met four criteria: (a) lower boundary of the CI 95% of the PRR (PRR025) greater than 1, (b) lower boundary of the CI 95% of the IC (IC025) greater than 0, (c) a PRR value greater than 2, and (d) have at least 100 reports with a Mood or Cognitive AE (columns Drug/Mood and Drug/Cognition combinations, respectively, in Online Resource 1), in the P/P subset of FAERS. The first three thresholds are commonly used in the literature (Caster et al. 2014; Poluzzi et al. 2012; Zink et al. 2013) whereas (d) was added to increase selection stringency. Based on these criteria, 29 pharmaceuticals were associated with Mood AEs and 32 pharmaceuticals with Cognition AEs (Fig. 1b). Table 1 also lists the top 10 drugs (by number of Drug/AE combinations) associated with Mood and Cognition AEs, respectively.

**Table 1:** Top 10 drugs associated with mood or cognitive adverse events

Drug name	No. of P/P FAERS reports	Drug/mood combinations	Drug/mood PRR	Drug/mood PRR025	Drug/mood IC025	Drug/cognitive combinations	Drug/cognitive PRR	Drug/cognitive PRR025	Drug/cognitive IC025
Top 10 Drugs (by number of Drug/mood combinations) associated with mood AEs									
Varenicline	36,666	12,180	2.23	2.2	1.07	3879	0.51	0.49	− 1.01
Levonorgestrel	12,232	4650	2.5	2.44	1.25	854	0.34	0.32	− 1.67
Isotretinoin	8224	4574	3.66	3.59	1.8	379	0.22	0.2	− 2.33
Drospirenone and ethinyl estradiol Combination	11,721	3860	2.15	2.1	1.04	475	0.2	0.18	− 2.49
Rofecoxib	8002	3540	2.9	2.83	1.46	1271	0.77	0.73	− 0.47
Metoclopramide	6611	2640	2.61	2.53	1.3	464	0.34	0.31	− 1.7
Alendronate	5910	2451	2.7	2.62	1.36	892	0.73	0.69	− 0.56
Apremilast	4887	1937	2.58	2.49	1.28	531	0.53	0.49	− 1.06
Aprotinin	2637	1923	4.75	4.64	2.16	36	0.07	0.05	− 4.45
Finasteride	3706	1854	3.26	3.15	1.62	1470	1.93	1.86	0.86

Drug name	No. of P/P FAERS reports	Drug/mood combinations	Drug/mood PRR	Drug/mood PRR025	Drug/mood IC025	Drug/cognitive combinations	Drug/cognitive PRR	Drug/cognitive PRR025	Drug/cognitive IC025
Top 10 Drugs (by number of Drug/cognitive combinations) associated with Cognitive AEs									
Natalizumab	24,838	2726	0.71	0.68	− 0.56	10,397	2.08	2.05	0.99
Dimethyl fumarate	11,638	1210	0.67	0.64	− 0.67	5060	2.14	2.09	1.03
Rivastigmine	3399	515	0.98	0.9	− 0.17	1690	2.43	2.35	1.19
Memantine	3029	389	0.83	0.76	− 0.44	1300	2.09	2.01	0.97
Temozolomide	1040	60	0.37	0.29	− 1.84	492	2.3	2.16	1.05
Cytarabine	1055	59	0.36	0.28	− 1.89	440	2.03	1.89	0.86
Etoposide	882	57	0.42	0.32	− 1.69	406	2.24	2.09	1
Ranolazine	875	33	0.24	0.17	− 2.6	398	2.21	2.06	0.98
PEG-interferon beta-1a	868	124	0.92	0.78	− 0.41	397	2.23	2.07	0.99
Indapamide	495	23	0.3	0.2	− 2.41	303	2.98	2.78	1.38

**Fig. 2:**
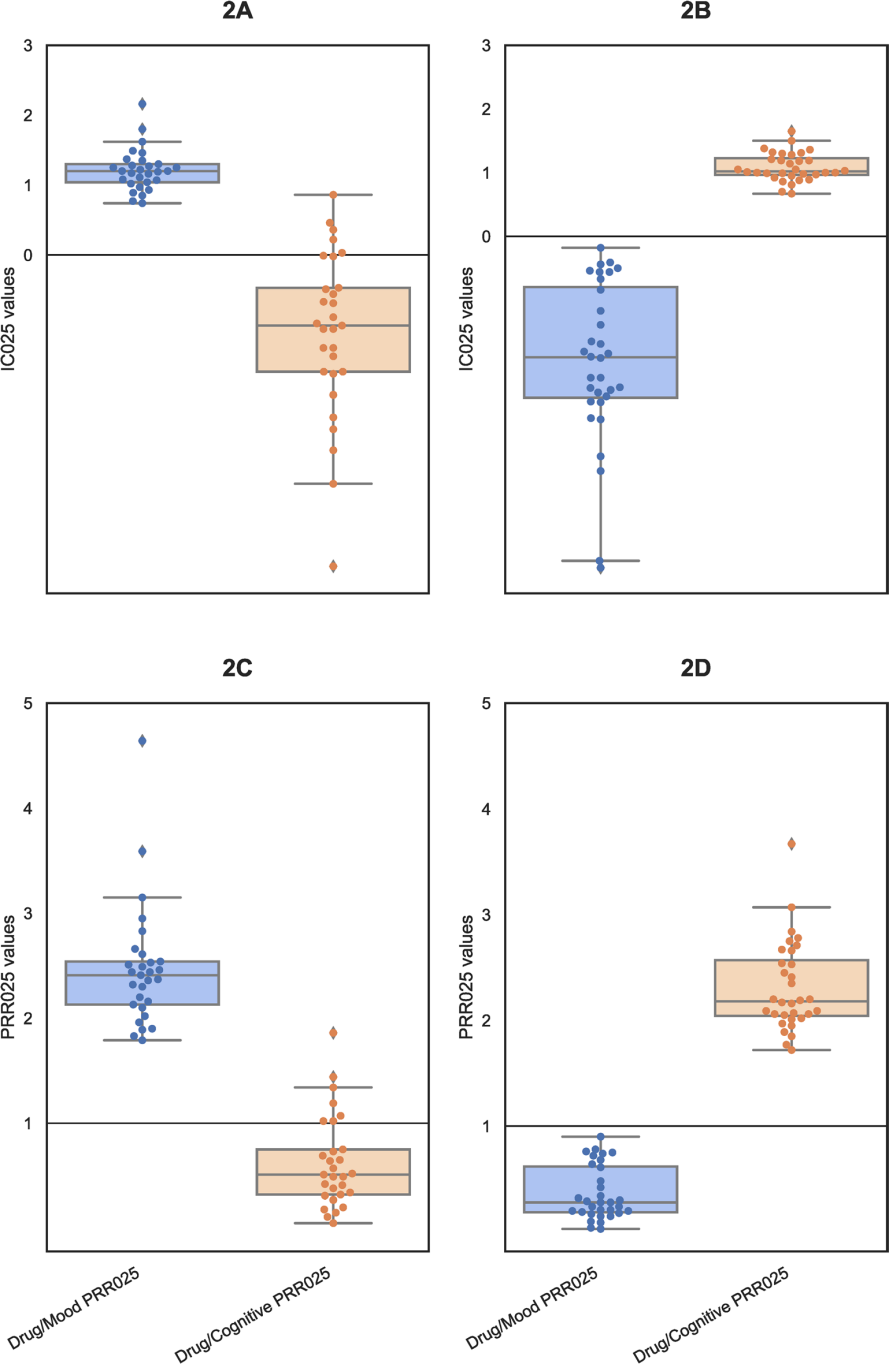
IC025 (**a**, **b**) and PRR025 (**c**, **d**) values of Drugs associated with either Mood or Cognitive AEs. IC025 represents the information component (*IC*)’s lower bound 95% Confidence Interval value. PRR025 indicates the Proportional Reporting Ratio (PRR)’s lower bound 95% confidence interval (*CI*) value. Drug—Mood AEs combinations are shown in blue. Drug—Cognitive AEs combinations are shown in orange (color figure online)

Highly interesting, pharmaceuticals conspicuously associated with Mood AEs were clearly separable from the ones associated with cognitive AEs. Specifically, when selecting IC025 values > 0 (Fig. 2a) and PRR025 values > 1 (Fig. 2c) for Drug–Mood AE combinations (in blue), the IC025 and PRR025 values for cognitive changes (in orange) were consistently below the corresponding thresholds. The inverse was also true: drugs associated with cognitive AEs (orange in Fig. 2b, d) were not associated with Mood AEs. This strong separation suggested the existence of differences in the molecular mechanisms underlying the two AE categories.

A complete list of pharmaceuticals and their disproportionality values is shown in Online Resource 1. Notably, the identified pharmaceuticals comprised different pharmaceutical classes, many of them being primarily CNS-acting drugs (e.g. antidepressants, antiepileptics), but also non-CNS-acting drugs (e.g. antineoplastic, immunomodulating agents).

### Signaling pathway analysis reveals similarities and differences in the molecular mechanisms underlying MCAEs

Following expert curation of the 232 genes associated with the FAERS-selected drugs, we identified altogether 120 CPEs directly associated with MCAEs: 58 associated with Mood- and 99 with Cognition-related AEs, while 30 CPEs were related to both Mood and Cognition AEs (listed in Online Resource 2). Those CPEs included several membrane-bound (e.g. dopamine, glutamate ionotropic, cholinergic nicotinic) and nuclear (e.g. vitamin D, progesterone, retinoic acid alpha) receptors, enzymes (e.g. phosphodiesterases, monoamine oxidase A/B), immune system-related components (e.g. interleukins, interferon-gamma), but also some other types of biomolecules. About a quarter of the identified CPEs (31 out of 120) represented receptors, and the modulation of some of them was previously identified in AOP databases as MIEs (e.g. modulation of glutamate ionotropic receptors) or KEs (e.g. regulation of serotonergic receptor 5-HTR3). Most of the identified CPEs are expressed in the CNS (83 out of 120), mainly in the cerebral cortex, cerebellum and/or hippocampus (Online Resource 2).

Pathway enrichment analysis (Online Resource 3) conducted via g:Profiler, using Reactome as its basis, revealed three classes of molecular mechanisms significantly associated with both Mood and Cognition AEs: (1) modulation of N-methyl-D-aspartate (NMDA) receptors, (2) neurotransmission, and (3) interleukin signaling. The main pathways associated with Mood AEs are listed in Online Resource 4. In addition, Cognitive AEs were significantly associated with RUNX and FOXP3 pathways, which regulate the development of T-lymphocytes, TP53 gene transcription, and are also involved in the modulation of acetylcholine nicotinic receptors (Online Resource 5).

**Fig. 3:**
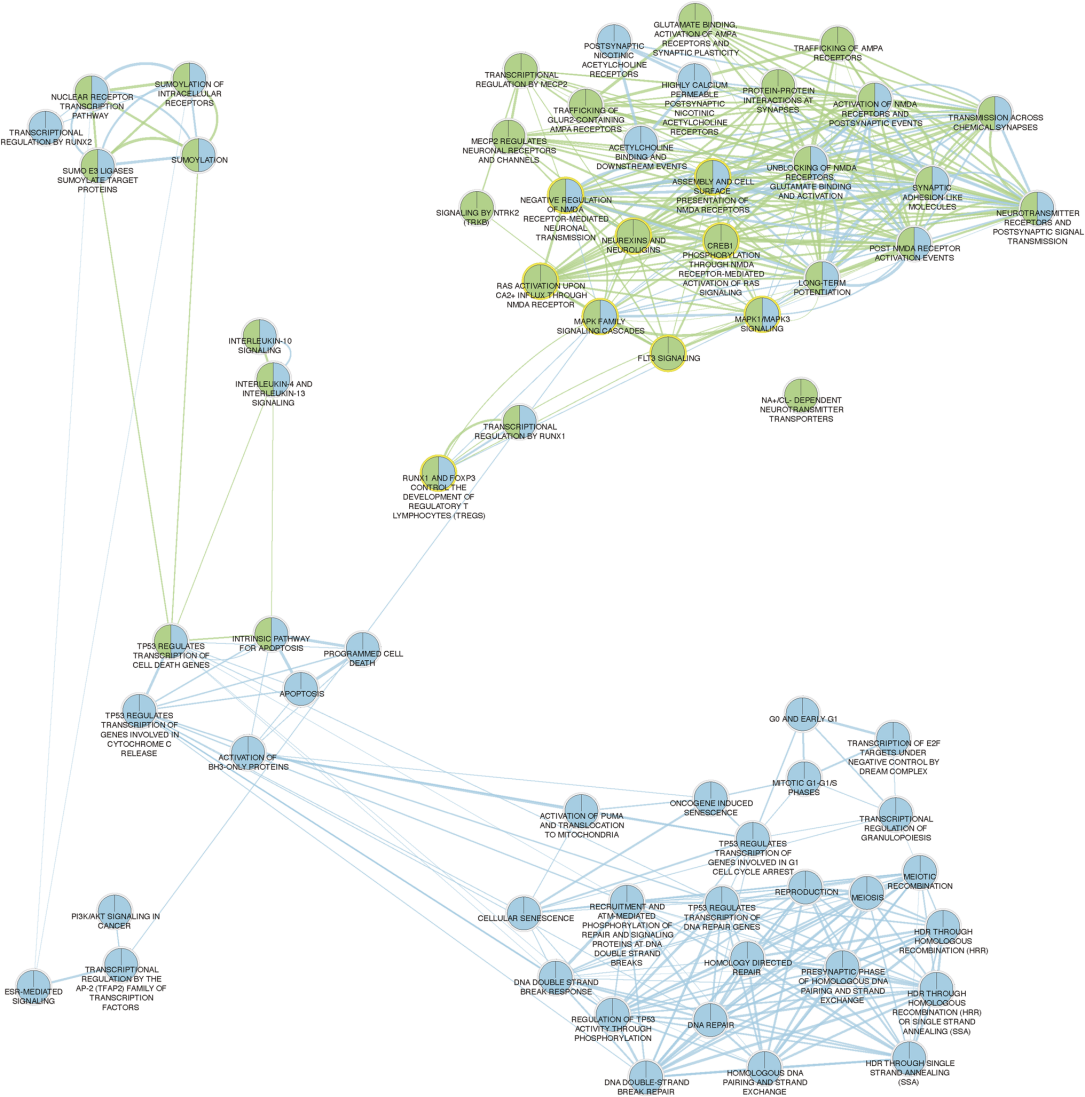
Pathway cross-talk analysis. **a** The interconnections among pathways associated with either mood- (green) or cognitive (blue)-related AEs was established using functional enrichment analysis and visualized as an enrichment map, as described in Supplemental Material Methods. Nodes common to both mood and cognitive AEs are represented in both green and blue colors. Data was further curated and displayed according to each type of AE-related pathways, namely Online Resource 7—Fig. 1: mood AEs-, and Online Resource 7—Fig. 2 cognitive AEs-related pathways (color figure online)

Pathway cross-talk analysis (Fig. 3) enabled the visualization of the commonalities and differences between Reactome pathways associated with Mood and Cognition AEs in the form of interconnected networks. Figure 3 evidences the high degree of overlap between pathways leading to MCAEs, as demonstrated by the high number of nodes simultaneously colored in green (Mood-related) and blue (Cognition-related). These common nodes were observed within two main clusters. The first one connected the modulation of NMDA receptors, MAPK-family signaling cascades, long-term potentiation and events related to synaptic neurotransmission. The second cluster connected interleukin signaling (i.e. IL-4 and -13, and IL-10) with the intrinsic apoptosis pathway, which in turn further linked to protein SUMOylation via TP53-mediated transcriptional regulation of cell death.

The analysis of pathway cross-talk for Mood AEs (Online Resource 7–Fig. 1) shows that pathways mainly grouped around the two clusters discussed above, with no apparent connection between them. However, other events seem to cluster around them. For example, modulation of trafficking and glutamate-mediated activation of α-amino-3-hydroxy-5-methyl-4-isoxazolepropionic acid (AMPA) receptors, as well as synaptic plasticity, represent additional nodes modulating neurotransmission and activation of NMDA receptors. MECP2-mediated transcriptional regulation of neuronal receptors and channels is associated with MAPK-family, as well as with the membrane-bound Neurotrophic Receptor Tyrosine Kinase 2 (NTRK2)-related signaling, both of which further associate with FLT3 signaling and transcription regulation by RUNX. Curiously, no apparent connection could be depicted between Na ^+^ ,Cl^−^-dependent neurotransmitter transporters and the other pathways.

Cognition-related pathway cross-talk analysis (Online Resource 7–Fig. 2) shows a more intricate clustering pattern, with no clearly separated clusters. This might be expected in light of the differences observed during our disproportionality analysis between the Mood and Cognition AEs (Fig. 2). Interestingly, some of the events involved in these cognition-related pathways seem to be regulated upstream of the events leading to Mood AEs. For example, some of the identified CPEs leading to cognitive deficits are involved in DNA repair-associated pathways (e.g. ABL Proto-Oncogene 1, Non-Receptor Tyrosine Kinase) and cell cycle regulation (e.g. cyclin-dependent kinases). These pathways and CPEs further connect with the activation of the intrinsic apoptotic pathway (common node for both types of AEs) by triggering cytochrome c release, activation and translocation of PUMA, and activation of BH3-only proteins at the mitochondrial level. The tumor proteins 53 (TP53) and 73 (TP73), Bcl-2, and E2F Transcription Factor 1 seem to play a major role in connecting these pathways.

The epidermal growth factor and estrogen receptors, which are involved in both estrogen receptor signaling and transcriptional regulation of the AP-2 family of transcription factors, seem to be important in connecting SUMOylation and nuclear receptor transcription pathways with the MAPK-family signaling in cognitive AEs. CPEs involved in pathways related to the post-synaptic activation of nicotinic acetylcholine receptors emerge as uniquely associated with cognitive AEs. Similarly to the regulation of NMDA receptors, the modulation of these receptors likely represents an early event in the cascade leading to cognitive deficits, further clustering with the neurotransmission-related pathways and MAPK-family signaling.

### Consolidation of MCAEs’ mechanisms via the AOP database

**Fig. 4:**
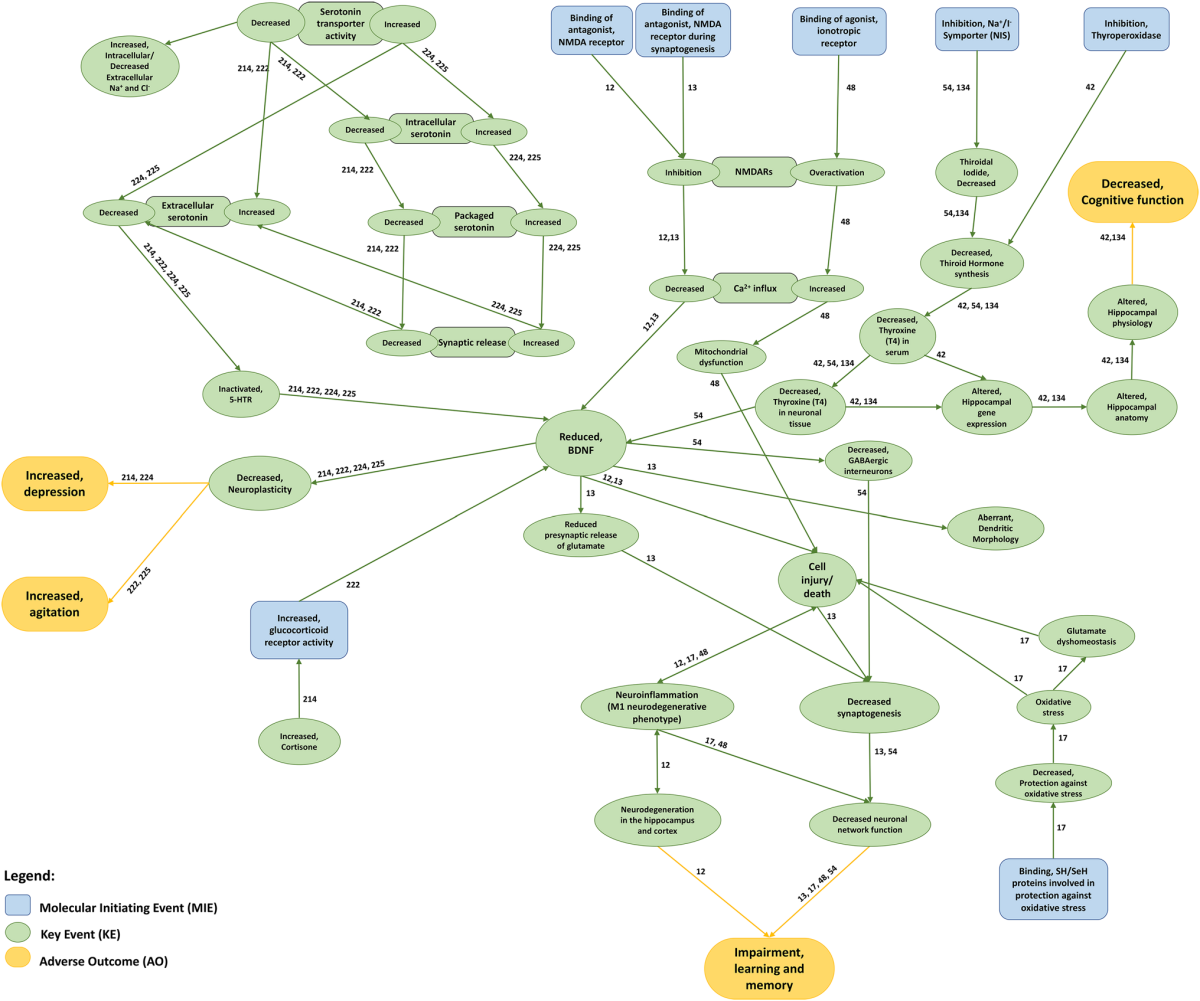
Interactions among AOPs associated with mood- and/or cognitive-related adverse outcomes. Molecular Initiating Events (blue boxes) trigger different downstream cascades of Key Events (green boxes) that ultimately lead to a mood- or cognitive-related Adverse Outcome (yellow boxes). Each AOP sequence of effects may be assessed by following its respective AOP ID next to each connecting edge. Information on MIEs, KEs and AOs herein depicted was retrieved from AOP databases (i.e. AOPWiki and AOP-KB) (color figure online)

Our query of toxicologist-curated OECD-managed databases retrieved 11 AOPs, summarized in Online Resource 6. Concerning cognitive changes, this Table shows that inhibition of NMDA receptors, binding to SH/SeH proteins involved in protection against oxidative stress, activation of ionotropic glutamate receptors or inhibition of Na ^+^ /I^−^ symporter (NIS) trigger a set of downstream events leading to learning and memory impairment (AOPs 12, 13, 17, 48 and 54). Binding of an antagonist to NMDA receptors (AOPs 12 and 13) or of an agonist to ionotropic receptors (AOP 48) were reported to modulate calcium influx, and either reduce BDNF levels or impair mitochondrial function, respectively, inducing cell death and/or decreased neurotransmitter release (Fig. 4). In fact, cell injury/death seems to be a common node to AOP 12, 13, 17 and 48. Both inhibition of NIS (AOPs 54, 134) and thyroperoxidase (AOP 42) impact thyroxine levels in neuronal tissues, specifically altering hippocampal gene expression, anatomy and physiology, resulting in cognitive function deterioration (Fig. 4).

Concerning adverse mood changes, increased serotonin transporter activity has been reported to modulate intra- and extracellular serotonin levels, resulting in either depression or agitation (AOPs 222, 224, 225). Additionally, activation of glucocorticoid receptors and increase of their activity has been proposed to initiate a sequence of events leading to depression and agitation (AOP 214). The reduction of BDNF levels seems to represent a common node to the majority of both MCAEs. This BDNF reduction is further responsible for a series of events, including decreased neuroplasticity, increased cell injury/death or decreased neurotransmitter (e.g. glutamate, GABA) release that results in decreased synaptogenesis or increased neuroinflammation, ultimately leading to learning and memory impairment (Fig. 4).

Noteworthy, AOPs 17, 134, 222, 224 and 225 are still under development. As such, information available regarding the KEs involved in those AOPs remains insufficiently understood.

### Marked contribution of immunomodulation to MCAEs

CPEs from Online Resource 2 involved in immunomodulatory functions were also identified by combining domain-specific databases (i.e. InnateDB, ImmPort) with expert-based literature curation of the database s-retrieved data. Online Resource 2 evidences that 79 out of all the identified CPEs (representing 66% of total elements) are associated with immunomodulation, emphasizing the importance of this broad mechanism to the onset of MCAEs. These CPEs mostly include cytokines (e.g. interferon-gamma, several interleukins), and interleukin receptors (e.g. IL-2 receptor). Noteworthy, some of these biomolecules (e.g. androgen receptor, IL-2 receptor) are not expressed in the CNS, or at least not to a considerable extent.

## Discussion

Our unique workflow unraveled valuable insights into the potential molecular mechanisms leading to MCAEs. As expected, several pathways identified as potentially triggering MCAEs have already been reported in existing AOPs, mostly assessed in rodent models (e.g. reduced BDNF levels, increased neuroinflammation). However, only a few well-documented AOPs causally relate MIEs and KEs to AOs in the CNS (Bal-Price et al. 2015), evidencing the scarce knowledge in the field. Specifically: (i) the identified AOPs mostly focused on brain-occurring MIEs/KEs, despite growing evidence that MCAEs may also result from the modulation of molecules outside the CNS (Capuron and Miller 2011); (ii) AOPs often fail to clearly associate a drug’s pharmacological action with a MIE, as they do not account for either targets’ subunits, site-specific regulation, nor possible off-targets; (iii) AOPs do not specify the molecules involved in a particular KE. Still, AOPs represent useful tools to determine whether a CPE modulation represents candidate early (MIE) or downstream events (KEs). For example, based on the comparison of our list of MCAE-related CPEs with identified AOPs, the modulation of the 5-HT3 receptor by metoclopramide, or glutamate ionotropic AMPA and NMDA receptors by perampanel and memantine, respectively, may be regarded as MIEs. On the other hand, the modulation of BDNF, a common KE to most of the identified AOPs (Fig. 4), by topotecan (Wick et al. 2004), or of pro-apoptotic protein Bax by interferon alpha-2 (Alvarez et al. 2002) may be regarded as late-stage events. Nonetheless, for most of the identified CPEs, it is very difficult to disentangle without the support of experimental data, whether they represent MIEs or KEs.

Beyond any doubt, some of the identified CPEs are likely to have important roles in the manifestation of MCAEs. For example, there is substantial evidence implicating glutamatergic neurotransmission (e.g. via NMDA and AMPA receptors) in the manifestation of mood disorders (Sequeira et al. 2009; Utge et al. 2010; Zarate et al. 2010), at least in part by governing synaptic plasticity and excitatory transmission in limbic pathways (Witkin et al. 2007). Changes in the glutamatergic system have been observed in the serum, cerebrospinal fluid (CSF) and brain tissue of patients with mood disorders (Hashimoto et al. 2007; Maeng and Zarate 2007). Particularly, genetic studies suggested the involvement of GRIN2B, which encodes the critical structural and functional NR2B subunit of NMDA receptors, in bipolar disorder and as a genetic predictor for treatment-resistant depression in major depressive disorder (Martucci et al. 2006; Zhang et al. 2014). Reduced levels of AMPA receptor subunits have also been reported in the striatum and prefrontal cortex of patients with mood disorders (Beneyto et al. 2007; Hashimoto et al. 2007; Meador-Woodruff et al. 2001; Scarr et al. 2003).

On the other hand, animals overexpressing the GluN1 subunit showed increased learning ability, supporting the involvement of impaired NMDA receptor function in cognitive disturbances (Tang et al. 2001). Additionally, GluN2C subunit ablation may result in relevant cognitive deficits in animals (Gupta and Chadda 2016).

Several lines of evidence support that changes in nicotinic acetylcholine (nACh) receptor signaling (identified in our study as CPE) are involved in mood regulation (Picciotto et al. 2015). By and large, an increased cholinergic neurotransmission activity, most likely mediated through overactivation of nACh receptors, has been associated with depressed mood states (Picciotto et al. 2015; Shytle et al. 2002). However, numerous studies have shown that suppression of a4b2 leads to positive effects on mood symptoms, an effect shared by the activation of a7nACh receptor signaling (reviewed by Picciotto et al. 2015), meaning that signaling modulation of different nACh receptors can have diverse outcomes on mood regulation. Additionally, single nucleotide polymorphisms in the promoter region of CHRNA7, encoding the a7nACh receptor subunit, significantly associated with schizophrenia (Stephens et al. 2009).

Apart from the nACh receptor system, the muscarinic receptor system has also been implicated in the pathophysiology of schizophrenia and depressive disorders (reviewed by Scarr 2009). For instance, animals that do not possess CHRM1 show working memory deficits, despite their normal hippocampal activity (Anagnostaras et al. 2003).

Most of the receptors identified as CPEs represented G-protein coupled receptors (GPCRs), suggesting the existence of converging downstream signaling pathways leading to MCAEs. Many GPCRs are known to activate cyclic adenosine monophosphate (cAMP) signaling and the subsequent Mitogen-Activated Protein kinases (MAPKs)-mediated cascade of events. Noteworthy, MAPKs mediate fundamental biological processes and cellular responses to external stress signals, including the regulation of synthesis of inflammatory mediators at transcription and translation levels (Kaminska 2005). Strikingly, our analysis clearly evidenced the importance of immunomodulation changes towards the onset of MCAEs.

More specifically, we showed that modulation of interleukin (i.e. IL-4, -10, and -13) signaling pathways represent common nodes in both Mood and Cognition AE-related mechanisms. They are possibly involved in these AEs by triggering intrinsic apoptotic pathways and/or affecting protein SUMOylation. Such data align with findings on the involvement of IL-4 and IL-13 production by meningeal T cells in maintaining cognitive function in mice (Brombacher et al. 2017; Derecki et al. 2010).

Surprisingly, we found no relationship between any of the interacting genes concerning four pharmaceuticals (aprotinin, ertapenem, natalizumab, and ranolazine) and MCAEs. Of these, only ranolazine is known to cross the blood–brain barrier (BBB), while the other should apparently have acted in an indirect manner (i.e. via off-target signaling). The importance of immune mediators was further strengthened by observing that some of the pharmaceuticals, for which a high occurrence of MCAEs has been reported, represent high molecular-weight biologicals (e.g. natalizumab, interferon alpha-2) that do not easily cross the BBB (Misiak et al. 2018). Indeed, immune-mediated inflammatory diseases have long been reported to co-occur with, or contribute to, mood changes, like depression (Nerurkar et al. 2019).

Immune modulation in the CNS [e.g. by affecting cytokines, chemokines, and other immune mediators or cells (e.g. microglia)] particularly represents an interesting and especially complex topic from the standpoint of anatomical microenvironment. The CNS is selectively permeable to proteins and peripheral immune cells (e.g. leukocytes) via (1) blood vessels within the BBB/blood-spinal cord barrier, (2) the blood-CSF barrier through an epithelial barrier in the choroid plexus, and (3) the meningeal lymphatic route (Louveau et al. 2017). Recently, Lutz et al. (2017) showed that circulating Th1 lymphocytes reach the CNS by using caveolae to go through the CNS blood vessel endothelial cells.

There are multiple potential scenarios with CNS barriers, peripheral immune system and CNS-resident immune cells as key players that require investigation, including: (i) direct regulation of drug transport function at CNS barriers by cytokines; (ii) facilitation of cytokines transport across CNS barriers into the brain by a drug; (iii) modulation of peripheral immune cells extravasation across CNS barriers by an (immunoactive) drug. The role of various endogenous and exogenous cytokines in BBB modulation has been explored broadly, mainly in vitro (Bauer et al. 2005; Hartz et al. 2006; Poller et al. 2010). Among well-studied mechanisms is modulation of P-glycoprotein (P-gp) by tumor necrosis factor-alpha which leads to a rapid decrease in P-gp mediated transport activity with no change in transporter protein expression, potentially affecting CNS exposure of a P-gp substrate drug that otherwise would have limited BBB transport (Erickson and Banks 2018). The rate and extent of cytokines transport across the CNS barriers have also been explored, with suggestions that their transport is saturable, and perhaps even specific for a certain cytokine/family of cytokines (Erickson and Banks 2018; Pan et al. 1997; Pan and Kastin 2002; Patel et al. 2012). However, the intricate mechanisms on how circulating factors, including drugs, may affect the transport of cytokines across the CNS barriers are not yet elucidated.

## Limitations

Our findings represent a cornerstone in the identification of candidate targets and pathways leading to MCAEs. Nevertheless, some limitations of our analysis, which do not compromise the overall findings, should be noted. First of all, it is highly possible that CPEs (e.g. receptors) with different binding sites may be site-specific regulated and/or that allosteric vs orthosteric binding may produce distinct outcomes. Moreover, as the analysis relies on both completeness and accuracy of the available sources, it is possible that a substantial number of potential binding sites of various drugs have not yet been identified.

It has been noted that pharmaceuticals with very few FAERS reports are frequently associated with higher values of PRR and IC. To alleviate this issue, we decided to add a minimum number of reports (100) as an extra filtering criterion, as well as a PRR value > 2 which increased stringency to the expense of missing potentially relevant drugs. In the future we plan to explore additional filtering methods like the Empirical Bayesian Geometric Mean (EGBM) scoring (https://www.fda.gov/science-research/data-mining/data-mining-fda-white-paper).

Although pathway cross-talk analysis provides interesting insights into CPEs and pathways that could be used as biomarkers to predict MCAEs at early stages of drug R&D, most of this information will need additional experimental validation (e.g. addressing how a particular drug administration regimen, i.e. acute vs chronic, impacts AEs). Also, novel and unexpected “hits” that resulted from our procedure should be carefully assessed experimentally to ascertain whether they are false positives or novel relevant elements of MCAEs’ underlying biology. Finally, we did not take into account the influence of external factors (e.g. environmental stress, diseases, drugs) on MCAEs via epigenetic modifications, due to scarce relevant data. Nevertheless, the impact of such modifications should not be disregarded, due to the growing evidence linking them to adverse mood changes (Labonte and Turecki 2012; Tsai et al. 2011).

## Conclusions

This work combined the power of statistical analysis of pharmacovigilance data with the refinement of expert literature curation to propose a set of CPEs consistent with a causal relationship to the onset of MCAEs, many of which are of considerable clinical importance. Pathway enrichment and cross-talk analyses, based on genes encoding for the identified CPEs, suggested a number of pathways that represent strong candidates for the observed AEs reported in FAERS that also overlap with existing AOPs. Comparison of this data with well-documented AOPs further allowed determining whether some of those targets could be considered MIEs, or how downstream a particular CPE modulation may occur in the molecular cascade of events. Additionally, we evidenced the key role played by immune modulation, particularly interleukin signaling, in MCAEs.

Our findings represent a valuable tool to improve experimental design (by tackling specific targets), and refine drug safety prediction at preclinical stages of drug R&D, considering the anticipation of potential neurotoxic AEs, namely those related to mood changes and cognitive deficits. The experimental data, which the NeuroDeRisk consortium aims to generate, will be of utmost importance to validate these findings and pave the way to the early detection of MCAEs. Considering the substantial overlap between molecular KEs occurring at later stages in the event cascade, we anticipate that the type of AE (i.e. Mood or Cognition) needs to be defined at an early stage of the pathway network.

## Electronic supplementary material

Below is the link to the electronic supplementary material.

Supplementary file1 (DOCX 64 kb)

Supplementary file2 (DOCX 90 kb)

Supplementary file3 (DOCX 26 kb)

Supplementary file4 (DOCX 25 kb)

Supplementary file5 (DOCX 28 kb)

Supplementary file6 (DOCX 24 kb)

Supplementary file7 (DOCX 439 kb)

## Abbreviations

AE: Adverse event
AO: Adverse outcome
AOP: Adverse outcome pathways
BBB: Blood–brain barrier
BCSFB: Blood-cerebrospinal fluid barrier
CNS: Central nervous system
CPE: Candidate pathway element
FAERS: FDA adverse event reporting system
KE: Key Event
MCAE: Mood and/or cognition adverse event
MIE: Molecular initiating event
MoA: Mechanism of action
PV: Pharmacovigilance
R&D: Research & development
