# Supplementary material for: Molecular basis of mood and cognitive adverse events elucidated via a combination of pharmacovigilance data mining and functional enrichment analysis
Source: Arch Toxicol. 2020 Jun 5;94(8):2829–45. doi: 10.1007/s00204-020-02788-1 (PMC7395038; doi:10.1007/s00204-020-02788-1)
Supplement: Supplementary file 1 — Supplementary file1 (DOCX 64 kb) [file 204_2020_2788_MOESM1_ESM.docx]

Online Resource 1

**Molecular basis of mood and cognitive adverse events elucidated via a combination of pharmacovigilance data mining and functional enrichment analysis**

Christos Andronis^1,*^, João Pedro Silva^2,*^, Eftychia Lekka^1^, Vassilis Virvilis^1^, Helena Carmo^2^, Konstantina Bampali^3^, Margot Ernst^3^, Yang Hu^4^, Irena Loryan^4^, Jacques Richard^5^, Félix Carvalho^2,#^, Miroslav M. Savić^6,#^

^1^Biovista, 34 Rodopoleos Street, 16777 Athens, Greece

^2^UCIBIO, REQUIMTE, Laboratory of Toxicology, Department of Biological Sciences, Faculty of Pharmacy, University of Porto, 4050-313, Porto, Portugal

^3^Department of Molecular Neurosciences, Medical University of Vienna, Spitalgasse 4, A-1090 Vienna, Austria

^4^Translational PKPD group, Department of Pharmaceutical Biosciences, Associate member of SciLifeLab, Uppsala University, Sweden

^5^Sanofi R&D, 371 avenue Professeur Blayac, Montpellier, 34000 France

^6^Department of Pharmacology, Faculty of Pharmacy, University of Belgrade, Vojvode Stepe 450, 11000 Belgrade, Serbia

*The authors contributed equally to the manuscript.

#Corresponding authors:

Félix Carvalho, UCIBIO, REQUIMTE, Laboratory of Toxicology, Faculty of Pharmacy, University of Porto, Portugal, Tel. +351 220428600, E-mail: felixdc@ff.up.pt; Miroslav Savić, Faculty of Pharmacy, University of Belgrade, Serbia, Tel. +381 113951280, E-mail: miroslav@pharmacy.bg.ac.rs

**Supplementary Table 1** – Pharmaceuticals with highest occurrences of mood and cognitive adverse events, selected following disproportionality analysis.

| **MOOD AEs-RELATED DRUGS** | | | | | | | | | | | | | | | | | |
| --- | --- | --- | --- | --- | --- | --- | --- | --- | --- | --- | --- | --- | --- | --- | --- | --- | --- |
| **Drug name** | **ATC**  **path(s)** | **Interacting**  **Genes** | **P/P FAERS reports** | **Years in FAERS** | **PP FAERS reports /year** | **Mood AEs** | **Drug/Mood combinations** | **Drug/Mood PRR** | **Drug/Mood PRR025** | **Drug/Mood IC** | **Drug/Mood IC025** | **Cognitive AEs** | **Drug/Cognitive**  **combinations** | **Drug/Cognitive PRR** | **Drug/Cognitive PRR025** | **Drug/Cognitive IC** | **Drug/Cognitive IC025** |
| **VARENICLINE** | NERVOUS SYSTEM DRUGS/OTHER NERVOUS SYSTEM DRUGS in ATC/DRUGS USED IN ADDICTIVE DISORDERS/Drugs used in nicotine dependence' | 'CHRNA4', 'CHRNB2', 'CHRNB4', 'CHRNA3', 'CHRNA7' | 36666 | 13 | 2820.46 | 'AGGRESSION', 'AGITATED DEPRESSION', 'ANHEDONIA', 'DEPRESSION', 'DEPRESSION SUICIDAL', 'DEPRESSIVE SYMPTOM', 'IMPULSIVE BEHAVIOUR', 'MAJOR DEPRESSION', 'SUICIDAL BEHAVIOUR', 'SUICIDAL IDEATION' | 12180 | 2.23 | 2.2 | 1.10 | 1.07 | 'AMNESIA', 'COGNITIVE DISORDER', 'CONFUSIONAL STATE', 'DELIRIUM', 'DEMENTIA', "DEMENTIA ALZHEIMER'S TYPE", 'DISORIENTATION', 'DISTURBANCE IN ATTENTION', 'MEMORY IMPAIRMENT', 'MENTAL STATUS CHANGES', 'SEDATION', 'TRANSIENT GLOBAL AMNESIA', 'FRONTOTEMPORAL DEMENTIA' | 3879 | 0.51 | 0.49 | -0.95 | -1.01 |
| **LEVONORGESTREL** | GENITO URINARY SYSTEM AND SEX HORMONES/SEX HORMONES AND MODULATORS OF THE GENITAL SYSTEM/HORMONAL CONTRACEPTIVES FOR SYSTEMIC USE/Progestogen systemic hormonal contraceptives', 'GENITO URINARY SYSTEM AND SEX HORMONES/SEX HORMONES AND MODULATORS OF THE GENITAL SYSTEM/HORMONAL CONTRACEPTIVES FOR SYSTEMIC USE/Emergency contraceptive drugs' | 'PGR', 'SRD5A1', 'ESR1', 'PGR' | 12232 | 15 | 815.47 | 'AGGRESSION', 'ANHEDONIA', 'DEPRESSION', 'DEPRESSION POSTOPERATIVE', 'DEPRESSION SUICIDAL', 'DEPRESSIVE DELUSION', 'DEPRESSIVE SYMPTOM', 'IMPULSIVE BEHAVIOUR', 'MAJOR DEPRESSION', 'PERSISTENT DEPRESSIVE DISORDER', 'SUICIDAL BEHAVIOUR', 'SUICIDAL IDEATION' | 4650 | 2.5 | 2.44 | 1.29 | 1.25 | 'AMNESIA', 'COGNITIVE DISORDER', 'CONFUSIONAL STATE', 'DELIRIUM', "DEMENTIA ALZHEIMER'S TYPE", 'DISORIENTATION', 'DISTURBANCE IN ATTENTION', 'MEMORY IMPAIRMENT', 'MENTAL STATUS CHANGES', 'SEDATION', 'TRANSIENT GLOBAL AMNESIA' | 854 | 0.34 | 0.32 | -1.55 | -1.67 |
| **ISOTRETINOIN** | DERMATOLOGICALS/ANTI-ACNE PREPARATIONS/ANTI-ACNE PREPARATIONS FOR TOPICAL USE/Retinoids for topical use in acne', 'DERMATOLOGICALS/ANTI-ACNE PREPARATIONS/ANTI-ACNE PREPARATIONS FOR SYSTEMIC USE/Retinoids for treatment of acne' | 'STAT2', 'RARG', 'RARA', 'TG', 'CA3', 'RARB', 'TGFB1', 'TGM2', 'BCL2', 'IL2RA', 'LPL' | 8224 | 15 | 547.33 | 'AGGRESSION', 'ANHEDONIA', 'DEPRESSION', 'DEPRESSION SUICIDAL', 'DEPRESSIVE SYMPTOM', 'IMPULSIVE BEHAVIOUR', 'MAJOR DEPRESSION', 'SUICIDAL BEHAVIOUR', 'SUICIDAL IDEATION' | 4574 | 3.66 | 3.59 | 1.84 | 1.8 | 'AMNESIA', 'COGNITIVE DISORDER', 'CONFUSIONAL STATE', 'DELIRIUM', 'DISORIENTATION', 'DISTURBANCE IN ATTENTION', 'MEMORY IMPAIRMENT', 'MENTAL STATUS CHANGES', 'SEDATION', "DEMENTIA ALZHEIMER'S TYPE" | 379 | 0.22 | 0.20 | -2.15 | -2.33 |
| **DROSPIRENONE AND ETHINYL ESTRADIOL COMBINATION** |  |  | 11721 | 15 | 781.07 | 'AGGRESSION', 'ANHEDONIA', 'DEPRESSION', 'DEPRESSION POSTOPERATIVE', 'DEPRESSION SUICIDAL', 'DEPRESSIVE SYMPTOM', 'IMPULSIVE BEHAVIOUR', 'MAJOR DEPRESSION', 'SUICIDAL BEHAVIOUR', 'SUICIDAL IDEATION' | 3860 | 2.15 | 2.1 | 1.09 | 1.04 | 'AMNESIA', 'COGNITIVE DISORDER', 'CONFUSIONAL STATE', 'DISORIENTATION', 'DISTURBANCE IN ATTENTION', 'MEMORY IMPAIRMENT', 'MENTAL STATUS CHANGES', 'SEDATION', 'DEMENTIA', 'FRONTOTEMPORAL DEMENTIA' | 475 | 0.2 | 0.18 | -2.34 | -2.49 |
| **ROFECOXIB** | MUSCULO-SKELETAL SYSTEM DRUGS/ANTIINFLAMMATORY AND ANTIRHEUMATIC PRODUCTS/ANTIINFLAMMATORY AND ANTIRHEUMATIC PRODUCTS, NON-STEROIDS/Coxibs' | 'GHRL', 'IL10', 'BIRC5', 'CPT2', 'PTGS2', 'BAX', 'HSPB1' | 8002 | 15 | 533.47 | 'DEPRESSION', 'AGGRESSION', 'MAJOR DEPRESSION', 'SUICIDAL IDEATION', 'ANHEDONIA', 'DEPRESSION POSTOPERATIVE', 'DEPRESSION SUICIDAL', 'DEPRESSIVE SYMPTOM' | 3540 | 2.9 | 2.83 | 1.51 | 1.46 | 'AMNESIA', 'DISORIENTATION', 'COGNITIVE DISORDER', 'CONFUSIONAL STATE', 'DELIRIUM', "DEMENTIA ALZHEIMER'S TYPE", 'MEMORY IMPAIRMENT', 'MENTAL STATUS CHANGES', 'SEDATION', 'DEMENTIA', 'DISTURBANCE IN ATTENTION' | 1271 | 0.77 | 0.73 | -0.37 | -0.47 |
| **METOCLOPRAMIDE** | ALIMENTARY TRACT AND METABOLISM DRUGS/DRUGS FOR FUNCTIONAL GASTROINTESTINAL DISORDERS/PROPULSIVES/Propulsives' | 'CHRM1', 'HTR3B', 'CYP1A2', 'IL2', 'G6PD', 'DRD2', 'CYP11B2' | 6611 | 16 | 413.06 | 'AGGRESSION', 'DEPRESSION', 'DEPRESSIVE SYMPTOM', 'IMPULSIVE BEHAVIOUR', 'MAJOR DEPRESSION', 'SUICIDAL IDEATION', 'ANHEDONIA', 'DEPRESSION SUICIDAL', 'SCHIZOAFFECTIVE DISORDER DEPRESSIVE TYPE', 'SUICIDAL BEHAVIOUR' | 2640 | 2.61 | 2.53 | 1.36 | 1.30 | 'MENTAL STATUS CHANGES', 'AMNESIA', 'COGNITIVE DISORDER', 'CONFUSIONAL STATE', 'DELIRIUM', 'DISORIENTATION', 'DISTURBANCE IN ATTENTION', 'MEMORY IMPAIRMENT', 'SEDATION', 'DEMENTIA', "DEMENTIA ALZHEIMER'S TYPE" | 464 | 0.34 | 0.31 | -1.7 | -1.70 |
| **ALENDRONATE** | MUSCULO-SKELETAL SYSTEM DRUGS/DRUGS FOR TREATMENT OF BONE DISEASES/DRUGS AFFECTING BONE STRUCTURE AND MINERALIZATION/Bisphosphonate drugs affecting bone structure and mineralization' | 'PLAU', 'IL11', 'FDPS', 'PTPRS', 'VDR', 'CYP19A1', 'PTPN4' | 5910 | 15 | 393.87 | 'AGGRESSION', 'AGITATED DEPRESSION', 'ANHEDONIA', 'DEPRESSION', 'DEPRESSION SUICIDAL', 'DEPRESSIVE SYMPTOM', 'IMPULSIVE BEHAVIOUR', 'MAJOR DEPRESSION', 'PERSISTENT DEPRESSIVE DISORDER', 'SUICIDAL IDEATION' | 2451 | 2.70 | 2.62 | 1.42 | 1.36 | 'AMNESIA', 'COGNITIVE DISORDER', 'CONFUSIONAL STATE', 'DELIRIUM', 'DEMENTIA', "DEMENTIA ALZHEIMER'S TYPE", 'DISORIENTATION', 'DISTURBANCE IN ATTENTION', 'FRONTOTEMPORAL DEMENTIA', 'MEMORY IMPAIRMENT', 'MENTAL STATUS CHANGES', 'SEDATION', 'TRANSIENT GLOBAL AMNESIA' | 892 | 0.73 | 0.69 | -0.44 | -0.56 |
| **APREMILAST** | ANTINEOPLASTIC AND IMMUNOMODULATING AGENTS/IMMUNOSUPPRESSANTS/IMMUNOSUPPRESSANTS/Selective immunosuppressants' | 'PDE4A', 'PDE4D', 'CDK6', 'CDK2', 'PDE4B', 'CDK4', 'PDE4C' | 4887 | 6 | 814.33 | 'AGGRESSION', 'ANHEDONIA', 'DEPRESSION', 'DEPRESSION SUICIDAL', 'DEPRESSIVE SYMPTOM', 'MAJOR DEPRESSION', 'SUICIDAL BEHAVIOUR', 'SUICIDAL IDEATION' | 1937 | 2.58 | 2.49 | 1.35 | 1.28 | 'AMNESIA', 'COGNITIVE DISORDER', 'CONFUSIONAL STATE', 'DELIRIUM', 'DEMENTIA', "DEMENTIA ALZHEIMER'S TYPE", 'DISORIENTATION', 'DISTURBANCE IN ATTENTION', 'MEMORY IMPAIRMENT', 'MENTAL STATUS CHANGES' | 531 | 0.53 | 0.49 | -0.92 | -1.06 |
| **APROTININ** | BLOOD AND BLOOD FORMING ORGAN DRUGS/ANTIHEMORRHAGICS/ANTIFIBRINOLYTICS/Proteinase inhibitors, antifibrinolytic' | 'KLK3', 'PLG' | 2637 | 9 | 292.89 | 'ANHEDONIA', 'DEPRESSION' | 1923 | 4.75 | 4.64 | 2.23 | 2.16 | 'CONFUSIONAL STATE', 'MENTAL STATUS CHANGES', 'AMNESIA', 'COGNITIVE DISORDER', 'DEMENTIA', 'DISTURBANCE IN ATTENTION', 'MEMORY IMPAIRMENT' | 36 | 0.07 | 0.05 | -3.89 | -4.45 |
| **FINASTERIDE** | DERMATOLOGICALS/OTHER DERMATOLOGICAL PREPARATIONS in ATC/OTHER DERMATOLOGICAL PREPARATIONS in ATC/OTHER DERMATOLOGICAL PREPARATIONS IN ATC', 'GENITO URINARY SYSTEM AND SEX HORMONES/UROLOGICALS/DRUGS USED IN BENIGN PROSTATIC HYPERTROPHY/Testosterone-5-alpha reductase inhibitors for benign prostatic hypertrophy' | 'GALK2', 'FGFR1', 'SRD5A2', 'CFLAR', 'SRD5A1', 'TGM2', 'AKR1D1' | 3706 | 15 | 247.07 | 'AGGRESSION', 'ANHEDONIA', 'DEPRESSION', 'DEPRESSION SUICIDAL', 'DEPRESSIVE SYMPTOM', 'IMPULSIVE BEHAVIOUR', 'MAJOR DEPRESSION', 'PERSISTENT DEPRESSIVE DISORDER', 'SUICIDAL BEHAVIOUR', 'SUICIDAL IDEATION' | 1854 | 3.26 | 3.15 | 1.69 | 1.62 | 'AMNESIA', 'COGNITIVE DISORDER', 'CONFUSIONAL STATE', 'DELIRIUM', 'DEMENTIA', "DEMENTIA ALZHEIMER'S TYPE", 'DISORIENTATION', 'DISTURBANCE IN ATTENTION', 'MEMORY IMPAIRMENT', 'MENTAL STATUS CHANGES', 'SEDATION' | 1470 | 1.93 | 1.86 | 0.94 | 0.86 |
| **MONTELUKAST** | RESPIRATORY SYSTEM DRUGS/DRUGS FOR OBSTRUCTIVE AIRWAY DISEASES/OTHER SYSTEMIC DRUGS FOR OBSTRUCTIVE AIRWAY DISEASES in ATC/Leukotriene receptor antagonists for obstructive airway diseases' | 'CYSLTR1', 'S1PR4', 'S1PR3', 'S1PR1' | 3673 | 15 | 244.87 | 'AGGRESSION', 'AGITATED DEPRESSION', 'ANHEDONIA', 'DEPRESSION', 'DEPRESSION SUICIDAL', 'DEPRESSIVE SYMPTOM', 'IMPULSIVE BEHAVIOUR', 'MAJOR DEPRESSION', 'SUICIDAL BEHAVIOUR', 'SUICIDAL IDEATION' | 1537 | 2.72 | 2.61 | 1.43 | 1.35 | 'AMNESIA', 'COGNITIVE DISORDER', 'CONFUSIONAL STATE', 'DELIRIUM', 'DEMENTIA', "DEMENTIA ALZHEIMER'S TYPE", 'DISORIENTATION', 'DISTURBANCE IN ATTENTION', 'MEMORY IMPAIRMENT', 'MENTAL STATUS CHANGES', 'SEDATION' | 345 | 0.45 | 0.41 | -1.13 | -1.30 |
| **PEGINTERFERON ALFA-2B** | ANTINEOPLASTIC AND IMMUNOMODULATING AGENTS/IMMUNOSTIMULANTS/IMMUNOSTIMULANTS/Interferons' | 'IFNL3', 'IFNAR2', 'IFNAR2', 'IFNAR1' | 3860 | 15 | 257.33 | 'AGGRESSION', 'AGITATED DEPRESSION', 'ANHEDONIA', 'DEPRESSION', 'DEPRESSION SUICIDAL', 'DEPRESSIVE SYMPTOM', 'IMPULSIVE BEHAVIOUR', 'MAJOR DEPRESSION', 'SUICIDAL BEHAVIOUR', 'SUICIDAL IDEATION' | 1344 | 2.26 | 2.16 | 1.17 | 1.08 | 'AMNESIA', 'COGNITIVE DISORDER', 'CONFUSIONAL STATE', 'DELIRIUM', 'DEMENTIA', "DEMENTIA ALZHEIMER'S TYPE", 'DISORIENTATION', 'DISTURBANCE IN ATTENTION', 'MEMORY IMPAIRMENT', 'MENTAL STATUS CHANGES', 'SEDATION' | 994 | 1.25 | 1.18 | 0.32 | 0.21 |
| **INTERFERON BETA-1B** | ANTINEOPLASTIC AND IMMUNOMODULATING AGENTS/IMMUNOSTIMULANTS/IMMUNOSTIMULANTS/Interferons' | 'IL2RA', 'IFNAR1', 'IFNAR2', 'IFNAR2', 'IL2' | 3256 | 15 | 217.07 | 'AGGRESSION', 'DEPRESSION', 'MAJOR DEPRESSION', 'SUICIDAL IDEATION', 'ANHEDONIA', 'DEPRESSION SUICIDAL', 'DEPRESSIVE DELUSION', 'DEPRESSIVE SYMPTOM', 'SUICIDAL BEHAVIOUR' | 1004 | 2.00 | 1.89 | 0.99 | 0.89 | 'AMNESIA', 'COGNITIVE DISORDER', 'CONFUSIONAL STATE', 'DELIRIUM', "DEMENTIA ALZHEIMER'S TYPE", 'DISORIENTATION', 'DISTURBANCE IN ATTENTION', 'MEMORY IMPAIRMENT', 'DEMENTIA', 'MENTAL STATUS CHANGES', 'SEDATION', 'TRANSIENT GLOBAL AMNESIA' | 726 | 1.08 | 1.01 | 0.11 | -0.01 |
| **ETHINYL ESTRADIOL; ETONOGESTREL** |  |  | 2122 | 15 | 141.47 | 'AGGRESSION', 'AGITATED DEPRESSION', 'ANHEDONIA', 'DEPRESSION', 'DEPRESSION SUICIDAL', 'DEPRESSIVE SYMPTOM', 'IMPULSIVE BEHAVIOUR', 'MAJOR DEPRESSION', 'PERSISTENT DEPRESSIVE DISORDER', 'SCHIZOAFFECTIVE DISORDER DEPRESSIVE TYPE', 'SUICIDAL BEHAVIOUR', 'SUICIDAL IDEATION' | 797 | 2.43 | 2.30 | 1.27 | 1.16 | 'AMNESIA', 'COGNITIVE DISORDER', 'CONFUSIONAL STATE', 'DISORIENTATION', 'DISTURBANCE IN ATTENTION', 'MEMORY IMPAIRMENT', 'MENTAL STATUS CHANGES', 'SEDATION' | 188 | 0.43 | 0.37 | -1.21 | -1.45 |
| **ETONOGESTREL** | GENITO URINARY SYSTEM AND SEX HORMONES/SEX HORMONES AND MODULATORS OF THE GENITAL SYSTEM/HORMONAL CONTRACEPTIVES FOR SYSTEMIC USE/Progestogen systemic hormonal contraceptives' | 'PGR', 'ESR1', 'PGR' | 1970 | 13 | 151.54 | 'AGGRESSION', 'AGITATED DEPRESSION', 'ANHEDONIA', 'DEPRESSION', 'DEPRESSION SUICIDAL', 'MAJOR DEPRESSION', 'SUICIDAL BEHAVIOUR', 'SUICIDAL IDEATION' | 653 | 2.14 | 2.01 | 1.09 | 0.97 | 'AMNESIA', 'COGNITIVE DISORDER', 'CONFUSIONAL STATE', 'DISORIENTATION', 'DISTURBANCE IN ATTENTION', 'MEMORY IMPAIRMENT', 'MENTAL STATUS CHANGES' | 76 | 0.18 | 0.15 | -2.40 | -2.78 |
| **MEDROXYPROGESTERONE ACETATE** | GENITO URINARY SYSTEM AND SEX HORMONES/SEX HORMONES AND MODULATORS OF THE GENITAL SYSTEM/HORMONAL CONTRACEPTIVES FOR SYSTEMIC USE/Progestogen systemic hormonal contraceptives', 'GENITO URINARY SYSTEM AND SEX HORMONES/SEX HORMONES AND MODULATORS OF THE GENITAL SYSTEM/PROGESTOGEN SEX HORMONES AND MODULATORS OF THE GENITAL SYSTEM/Pregnen (4) derivatives, progestogens', 'ANTINEOPLASTIC AND IMMUNOMODULATING AGENTS/ENDOCRINE THERAPY ANTINEOPLASTIC AND IMMUNOMODULATING AGENTS/HORMONES AND RELATED AGENTS/Progestogens, endocrine therapy drugs' | 'PGR', 'BAX', 'LSS', 'CXCL8', 'MMP1', 'CYP1B1' | 1456 | 15 | 97.07 | 'ANHEDONIA', 'DEPRESSION', 'SUICIDAL IDEATION', 'AGGRESSION', 'DEPRESSION POSTOPERATIVE', 'DEPRESSION SUICIDAL', 'IMPULSIVE BEHAVIOUR', 'MAJOR DEPRESSION', 'SUICIDAL BEHAVIOUR', 'DEPRESSIVE SYMPTOM' | 566 | 2.51 | 2.36 | 1.32 | 1.18 | 'AMNESIA', 'COGNITIVE DISORDER', 'DEMENTIA', 'DISTURBANCE IN ATTENTION', 'MEMORY IMPAIRMENT', 'CONFUSIONAL STATE', 'DELIRIUM', "DEMENTIA ALZHEIMER'S TYPE", 'DISORIENTATION', 'MENTAL STATUS CHANGES' | 180 | 0.60 | 0.52 | -0.73 | -0.98 |
| **VALDECOXIB** | MUSCULO-SKELETAL SYSTEM DRUGS/ANTIINFLAMMATORY AND ANTIRHEUMATIC PRODUCTS/ANTIINFLAMMATORY AND ANTIRHEUMATIC PRODUCTS, NON-STEROIDS/Coxibs' | 'CA12', 'CPT2', 'PTGS2', 'BIRC5' | 1383 | 12 | 115.25 | 'AGGRESSION', 'ANHEDONIA', 'DEPRESSION', 'DEPRESSIVE SYMPTOM', 'MAJOR DEPRESSION', 'SUICIDAL BEHAVIOUR', 'SUICIDAL IDEATION' | 550 | 2.57 | 2.41 | 1.36 | 1.22 | 'AMNESIA', 'COGNITIVE DISORDER', 'CONFUSIONAL STATE', 'DELIRIUM', 'DEMENTIA', "DEMENTIA ALZHEIMER'S TYPE", 'DISORIENTATION', 'DISTURBANCE IN ATTENTION', 'MEMORY IMPAIRMENT', 'MENTAL STATUS CHANGES', 'SEDATION' | 318 | 1.11 | 1.01 | 0.15 | -0.02 |
| **TETRABENAZINE** | NERVOUS SYSTEM DRUGS/OTHER NERVOUS SYSTEM DRUGS in ATC/OTHER NERVOUS SYSTEM DRUGS in ATC/Other nervous system drugs in ATC' | 'SLC18A2', 'SLC18A2', 'CYP2D6', 'SLC18A1' | 1540 | 12 | 128.33 | 'DEPRESSION', 'AGGRESSION', 'AGITATED DEPRESSION', 'ANHEDONIA', 'DEPRESSION SUICIDAL', 'DEPRESSIVE SYMPTOM', 'IMPULSIVE BEHAVIOUR', 'MAJOR DEPRESSION', 'SUICIDAL BEHAVIOUR', 'SUICIDAL IDEATION' | 503 | 2.11 | 1.96 | 1.07 | 0.93 | 'AMNESIA', 'COGNITIVE DISORDER', 'CONFUSIONAL STATE', 'DELIRIUM', 'DEMENTIA', "DEMENTIA ALZHEIMER'S TYPE", 'DISORIENTATION', 'DISTURBANCE IN ATTENTION', 'MEMORY IMPAIRMENT', 'MENTAL STATUS CHANGES', 'SEDATION' | 229 | 0.72 | 0.64 | -0.46 | -0.68 |
| **ALENDRONATE SODIUM; CHOLECALCIFEROL** |  |  | 876 | 13 | 67.38 | 'ANHEDONIA', 'DEPRESSION', 'MAJOR DEPRESSION', 'SUICIDAL IDEATION', 'AGGRESSION', 'AGITATED DEPRESSION', 'DEPRESSION SUICIDAL', 'DEPRESSIVE SYMPTOM' | 429 | 3.17 | 2.96 | 1.66 | 1.50 | 'AMNESIA', 'CONFUSIONAL STATE', 'DELIRIUM', 'DEMENTIA', "DEMENTIA ALZHEIMER'S TYPE", 'DISORIENTATION', 'DISTURBANCE IN ATTENTION', 'MEMORY IMPAIRMENT', 'MENTAL STATUS CHANGES', 'SEDATION', 'TRANSIENT GLOBAL AMNESIA', 'COGNITIVE DISORDER' | 137 | 0.75 | 0.65 | -0.39 | -0.67 |
| **MEFLOQUINE** | ANTIPARASITIC PRODUCTS, INSECTICIDES AND REPELLENTS/ANTIPROTOZOALS/ANTIMALARIALS/Methanolquinolines, antimalarial' | 'P2RY11', 'BDKRB1' | 678 | 15 | 45.2 | 'AGGRESSION', 'ANHEDONIA', 'DEPRESSION', 'IMPULSIVE BEHAVIOUR', 'MAJOR DEPRESSION', 'SUICIDAL IDEATION', 'SUICIDAL BEHAVIOUR', 'DEPRESSIVE SYMPTOM' | 290 | 2.77 | 2.53 | 1.46 | 1.27 | 'AMNESIA', 'COGNITIVE DISORDER', 'CONFUSIONAL STATE', 'DELIRIUM', 'DEMENTIA', 'DISORIENTATION', 'DISTURBANCE IN ATTENTION', 'MEMORY IMPAIRMENT', 'MENTAL STATUS CHANGES', 'SEDATION' | 225 | 1.61 | 1.44 | 0.68 | 0.46 |
| **PAMIDRONATE** | MUSCULO-SKELETAL SYSTEM DRUGS/DRUGS FOR TREATMENT OF BONE DISEASES/DRUGS AFFECTING BONE STRUCTURE AND MINERALIZATION/Bisphosphonate drugs affecting bone structure and mineralization' | 'CD86', 'SLC19A1', 'FDPS', 'PTH', 'CASR', 'CXCL8' | 620 | 15 | 41.33 | 'AGGRESSION', 'ANHEDONIA', 'DEPRESSION', 'DEPRESSIVE SYMPTOM', 'MAJOR DEPRESSION', 'SUICIDAL IDEATION', 'SUICIDAL BEHAVIOUR' | 264 | 2.75 | 2.51 | 1.45 | 1.25 | 'AMNESIA', 'COGNITIVE DISORDER', 'CONFUSIONAL STATE', 'DELIRIUM', 'DEMENTIA', "DEMENTIA ALZHEIMER'S TYPE", 'DISORIENTATION', 'DISTURBANCE IN ATTENTION', 'MEMORY IMPAIRMENT', 'MENTAL STATUS CHANGES', 'SEDATION' | 156 | 1.22 | 1.06 | 0.28 | 0.02 |
| **ESTROGENS, CONJUGATED; MEDROXYPROGESTERONE ACETATE** |  |  | 764 | 15 | 50.93 | 'AGGRESSION', 'ANHEDONIA', 'DEPRESSION', 'DEPRESSION SUICIDAL', 'MAJOR DEPRESSION', 'SUICIDAL BEHAVIOUR', 'SUICIDAL IDEATION' | 247 | 2.09 | 1.88 | 1.06 | 0.85 | 'AMNESIA', 'COGNITIVE DISORDER', 'CONFUSIONAL STATE', 'DEMENTIA', 'DISORIENTATION', 'DISTURBANCE IN ATTENTION', 'MEMORY IMPAIRMENT', 'MENTAL STATUS CHANGES', 'SEDATION' | 106 | 0.67 | 0.56 | -0.56 | -0.88 |
| **INTERFERON ALPHA-2** | ANTINEOPLASTIC AND IMMUNOMODULATING AGENTS/IMMUNOSTIMULANTS/IMMUNOSTIMULANTS/Interferons' | 'IL6', 'IFNAR2', 'EIF2AK2', 'FGFR2', 'TG', 'BAX', 'TGFB1', 'IFNG', 'IFNAR1', 'TP53', 'CSF2', 'NAT2' | 669 | 15 | 44.6 | 'DEPRESSION', 'MAJOR DEPRESSION', 'AGGRESSION', 'DEPRESSIVE SYMPTOM', 'SUICIDAL IDEATION', 'ANHEDONIA' | 243 | 2.35 | 2.12 | 1.22 | 1.01 | 'AMNESIA', 'COGNITIVE DISORDER', 'CONFUSIONAL STATE', 'DEMENTIA', 'DISORIENTATION', 'DISTURBANCE IN ATTENTION', 'MEMORY IMPAIRMENT', 'MENTAL STATUS CHANGES', 'DELIRIUM', "DEMENTIA ALZHEIMER'S TYPE", 'SEDATION' | 207 | 1.50 | 1.34 | 0.58 | 0.35 |
| **PERAMPANEL** | NERVOUS SYSTEM DRUGS/ANTIEPILEPTICS/ANTIEPILEPTICS/Other antiepileptics in ATC' | 'GRIA2', 'GRIA4', 'GRIA3', 'GRIA1' | 576 | 7 | 82.29 | 'AGGRESSION', 'DEPRESSION', 'DEPRESSION SUICIDAL', 'IMPULSIVE BEHAVIOUR', 'MAJOR DEPRESSION', 'SUICIDAL BEHAVIOUR', 'SUICIDAL IDEATION' | 233 | 2.61 | 2.37 | 1.38 | 1.16 | 'AMNESIA', 'COGNITIVE DISORDER', 'CONFUSIONAL STATE', 'DELIRIUM', "DEMENTIA ALZHEIMER'S TYPE", 'DISORIENTATION', 'DISTURBANCE IN ATTENTION', 'MEMORY IMPAIRMENT', 'MENTAL STATUS CHANGES', 'SEDATION' | 73 | 0.61 | 0.49 | -0.69 | -1.08 |
| **GADODIAMIDE** | VARIOUS DRUG CLASSES IN ATC/CONTRAST MEDIA/MAGNETIC RESONANCE IMAGING CONTRAST MEDIA/Paramagnetic contrast media' |  | 483 | 14 | 34.5 | 'AGGRESSION', 'ANHEDONIA', 'DEPRESSION', 'MAJOR DEPRESSION', 'SUICIDAL IDEATION' | 202 | 2.70 | 2.43 | 1.43 | 1.19 | 'AMNESIA', 'COGNITIVE DISORDER', 'CONFUSIONAL STATE', 'DISORIENTATION', 'DISTURBANCE IN ATTENTION', 'MEMORY IMPAIRMENT', 'MENTAL STATUS CHANGES' | 54 | 0.54 | 0.42 | -0.87 | -1.32 |
| **GADOVERSETAMIDE** | VARIOUS DRUG CLASSES IN ATC/CONTRAST MEDIA/MAGNETIC RESONANCE IMAGING CONTRAST MEDIA/Paramagnetic contrast media' |  | 406 | 11 | 36.91 | 'ANHEDONIA', 'DEPRESSION', 'MAJOR DEPRESSION', 'SUICIDAL IDEATION' | 173 | 2.75 | 2.46 | 1.45 | 1.20 | 'AMNESIA', 'COGNITIVE DISORDER', 'CONFUSIONAL STATE', 'DISTURBANCE IN ATTENTION', 'MEMORY IMPAIRMENT', 'MENTAL STATUS CHANGES', 'DELIRIUM', 'DISORIENTATION' | 38 | 0.45 | 0.33 | -1.12 | -1.66 |
| **GADOTERIDOL** | VARIOUS DRUG CLASSES IN ATC/CONTRAST MEDIA/MAGNETIC RESONANCE IMAGING CONTRAST MEDIA/Paramagnetic contrast media' |  | 341 | 14 | 24.36 | 'ANHEDONIA', 'DEPRESSION', 'MAJOR DEPRESSION', 'SUICIDAL IDEATION' | 139 | 2.63 | 2.32 | 1.39 | 1.11 | 'AMNESIA', 'COGNITIVE DISORDER', 'CONFUSIONAL STATE', 'DISORIENTATION', 'DISTURBANCE IN ATTENTION', 'MEMORY IMPAIRMENT', 'MENTAL STATUS CHANGES' | 27 | 0.38 | 0.26 | -1.36 | -2.00 |
| **PHENELZINE** | NERVOUS SYSTEM DRUGS/PSYCHOANALEPTICS/ANTIDEPRESSANTS/Monoamine oxidase inhibitors, non-selective' | 'SLC6A2', 'MAOA', 'SUCNR1', 'AOC3', 'MAOB', 'CYP2C8', 'SLC6A4', 'SLC6A3' | 412 | 15 | 27.47 | 'AGGRESSION', 'DEPRESSION', 'SUICIDAL IDEATION', 'DEPRESSION SUICIDAL', 'MAJOR DEPRESSION' | 131 | 2.05 | 1.78 | 1.03 | 0.74 | 'CONFUSIONAL STATE', 'MENTAL STATUS CHANGES', 'AMNESIA', 'COGNITIVE DISORDER', 'DEMENTIA', 'DISORIENTATION', 'DISTURBANCE IN ATTENTION', 'MEMORY IMPAIRMENT', 'SEDATION' | 78 | 0.91 | 0.75 | -0.11 | -0.49 |
| **BEYAZ** |  |  | 327 | 9 | 36.33 | 'ANHEDONIA', 'DEPRESSION', 'DEPRESSIVE SYMPTOM', 'SUICIDAL IDEATION' | 108 | 2.13 | 1.83 | 1.08 | 0.76 | 'AMNESIA', 'COGNITIVE DISORDER', 'CONFUSIONAL STATE', 'DISORIENTATION', 'MEMORY IMPAIRMENT', 'MENTAL STATUS CHANGES', 'DISTURBANCE IN ATTENTION' | 13 | 0.19 | 0.11 | -2.32 | -3.26 |
| **COGNITIVE AEs-RELATED DRUGS** | | | | | | | | | | | | | | | | | |
| **Drug name** | **ATC**  **path(s)** | **Interacting**  **Genes** | **P/P FAERS reports** | **Years in FAERS** | **PP FAERS reports /year** | **Mood AEs** | **Drug/Mood combinations** | **Drug/Mood PRR** | **Drug/Mood PRR025** | **Drug/Mood IC** | **Drug/Mood IC025** | **Cognitive AEs** | **Drug/Cognitive**  **combinations** | **Drug/Cognitive PRR** | **Drug/Cognitive PRR025** | **Drug/Cognitive IC** | **Drug/Cognitive IC025** |
| **NATALIZUMAB** | ANTINEOPLASTIC AND IMMUNOMODULATING AGENTS/IMMUNOSUPPRESSANTS/IMMUNOSUPPRESSANTS/Selective immunosuppressants' | 'ITGB1', 'ITGA4', 'ITGB7', 'ITGB1', 'ITGA4' | 24838 | 14 | 1774.14 | 'AGGRESSION', 'ANHEDONIA', 'DEPRESSION', 'DEPRESSION POSTOPERATIVE', 'DEPRESSION SUICIDAL', 'DEPRESSIVE SYMPTOM', 'IMPULSIVE BEHAVIOUR', 'MAJOR DEPRESSION', 'PERSISTENT DEPRESSIVE DISORDER', 'SUICIDAL BEHAVIOUR', 'SUICIDAL IDEATION' | 2726 | 0.71 | 0.68 | -0.49 | -0.56 | 'AMNESIA', 'COGNITIVE DISORDER', 'CONFUSIONAL STATE', 'DELIRIUM', 'DEMENTIA', "DEMENTIA ALZHEIMER'S TYPE", 'DISORIENTATION', 'DISTURBANCE IN ATTENTION', 'FRONTOTEMPORAL DEMENTIA', 'MEMORY IMPAIRMENT', 'MENTAL STATUS CHANGES', 'SEDATION', 'TRANSIENT GLOBAL AMNESIA' | 10397 | 2.08 | 2.05 | 1.02 | 0.99 |
| **DIMETHYL FUMARATE** | NERVOUS SYSTEM DRUGS/OTHER NERVOUS SYSTEM DRUGS in ATC/OTHER NERVOUS SYSTEM DRUGS in ATC/Other nervous system drugs in ATC' | 'KEAP1' | 11638 | 7 | 1662.57 | 'MAJOR DEPRESSION', 'AGGRESSION', 'AGITATED DEPRESSION', 'ANHEDONIA', 'DEPRESSION', 'DEPRESSION SUICIDAL', 'DEPRESSIVE SYMPTOM', 'IMPULSIVE BEHAVIOUR', 'PERSISTENT DEPRESSIVE DISORDER', 'SUICIDAL BEHAVIOUR', 'SUICIDAL IDEATION' | 1210 | 0.67 | 0.64 | -0.57 | -0.67 | 'AMNESIA', 'COGNITIVE DISORDER', 'CONFUSIONAL STATE', 'DELIRIUM', 'DEMENTIA', "DEMENTIA ALZHEIMER'S TYPE", 'DISORIENTATION', 'DISTURBANCE IN ATTENTION', 'FRONTOTEMPORAL DEMENTIA', 'MEMORY IMPAIRMENT', 'MENTAL STATUS CHANGES', 'SEDATION', 'TRANSIENT GLOBAL AMNESIA' | 5060 | 2.14 | 2.09 | 1.07 | 1.03 |
| **RIVASTIGMINE** | NERVOUS SYSTEM DRUGS/PSYCHOANALEPTICS/ANTI-DEMENTIA DRUGS/Anticholinesterase anti-dementia drugs' | 'BCHE', 'ACHE' | 3399 | 15 | 226.4 | 'AGGRESSION', 'DEPRESSION', 'DEPRESSIVE SYMPTOM', 'IMPULSIVE BEHAVIOUR', 'MAJOR DEPRESSION', 'SUICIDAL IDEATION', 'DEPRESSION SUICIDAL' | 515 | 0.98 | 0.90 | -0.03 | -0.17 | 'AMNESIA', 'COGNITIVE DISORDER', 'CONFUSIONAL STATE', 'DELIRIUM', 'DEMENTIA', "DEMENTIA ALZHEIMER'S TYPE", 'DISORIENTATION', 'DISTURBANCE IN ATTENTION', 'FRONTOTEMPORAL DEMENTIA', 'MEMORY IMPAIRMENT', 'MENTAL STATUS CHANGES', 'SEDATION' | 1690 | 2.43 | 2.35 | 1.27 | 1.19 |
| **MEMANTINE** | NERVOUS SYSTEM DRUGS/PSYCHOANALEPTICS/ANTI-DEMENTIA DRUGS/Other anti-dementia drugs in ATC' | 'GRIN2A', 'GRIN3A', 'GRIN1', 'GRIN3B', 'GRIN2D', 'GRIN2B', 'GRIN2C' | 3029 | 15 | 201.93 | 'AGGRESSION', 'DEPRESSION', 'SUICIDAL IDEATION', 'DEPRESSIVE SYMPTOM', 'IMPULSIVE BEHAVIOUR', 'MAJOR DEPRESSION', 'SUICIDAL BEHAVIOUR', 'ANHEDONIA' | 389 | 0.83 | 0.76 | -0.26 | -0.44 | 'CONFUSIONAL STATE', 'DELIRIUM', 'COGNITIVE DISORDER', "DEMENTIA ALZHEIMER'S TYPE", 'DISORIENTATION', 'DISTURBANCE IN ATTENTION', 'AMNESIA', 'DEMENTIA', 'FRONTOTEMPORAL DEMENTIA', 'MEMORY IMPAIRMENT', 'MENTAL STATUS CHANGES', 'SEDATION', 'TRANSIENT GLOBAL AMNESIA' | 1300 | 2.09 | 2.01 | 1.05 | 0.97 |
| **TEMOZOLOMIDE** | ANTINEOPLASTIC AND IMMUNOMODULATING AGENTS/ANTINEOPLASTIC AGENTS/ALKYLATING AGENTS/Other alkylating agents in ATC' | 'ATM', 'MSH6', 'TP53', 'H2AFX', 'ATRX', 'IDH1', 'MGMT', 'BRCA1', 'CSF2', 'PTEN', 'STAG2', 'EGFR', 'MYC', 'ATR', 'PRL', 'NOTCH1' | 1040 | 15 | 69.13 | 'AGGRESSION', 'DEPRESSION', 'DEPRESSIVE SYMPTOM', 'SUICIDAL IDEATION' | 60 | 0.37 | 0.29 | -1.41 | -1.84 | 'AMNESIA', 'COGNITIVE DISORDER', 'CONFUSIONAL STATE', 'DELIRIUM', 'DEMENTIA', "DEMENTIA ALZHEIMER'S TYPE", 'DISORIENTATION', 'DISTURBANCE IN ATTENTION', 'MEMORY IMPAIRMENT', 'MENTAL STATUS CHANGES', 'FRONTOTEMPORAL DEMENTIA', 'SEDATION' | 492 | 2.30 | 2.16 | 1.20 | 1.05 |
| **CYTARABINE** | ANTINEOPLASTIC AND IMMUNOMODULATING AGENTS/ANTINEOPLASTIC AGENTS/ANTIMETABOLITES/Pyrimidine analogues, antineoplastic antimetabolites | 'MAGEA1', 'WT1', 'BIRC5', 'TP53', 'VDR', 'FLT3', 'CDA', 'IL1B', 'HSPA4', 'CSF2', 'SLC29A1', 'RUNX1', 'CCND1', 'POLE', 'KMT2A', 'FCGR3B', 'XIAP', 'GATA1', 'NRAS', 'POLB', 'NRG1', 'KIT', 'ITGAV', 'POLA1', 'FCGR3A', 'GSTP1', 'NTRK1', 'POLD1', 'TP73' | 1055 | 15 | 70.33 | 'DEPRESSION', 'AGGRESSION', 'ANHEDONIA', 'MAJOR DEPRESSION', 'SUICIDAL IDEATION' | 59 | 0.36 | 0.28 | -1.46 | -1.89 | 'COGNITIVE DISORDER', 'CONFUSIONAL STATE', 'DELIRIUM', 'DEMENTIA', 'DISORIENTATION', 'DISTURBANCE IN ATTENTION', 'AMNESIA', 'FRONTOTEMPORAL DEMENTIA', 'MEMORY IMPAIRMENT', 'MENTAL STATUS CHANGES', 'SEDATION' | 440 | 2.03 | 1.89 | 1.02 | 0.86 |
| **ETOPOSIDE** | ANTINEOPLASTIC AND IMMUNOMODULATING AGENTS/ANTINEOPLASTIC AGENTS/PLANT ALKALOIDS AND OTHER NATURAL PRODUCTS, ANTINEOPLASTIC DRUGS/Podophyllotoxin derivatives, antineoplastic drugs' | 'MYCN', 'BRCA2', 'EIF4E', 'PGP', 'BAX', 'ABCC1', 'TOP2A', 'AFP', 'ITGAL', 'KLK3', 'EGFR', 'IGF2', 'TOP2B', 'UGT1A10', 'GDF15', 'XIAP', 'PLA2G1B', 'FGFR1', 'BTF3P11', 'E2F1', 'TNFSF13B', 'DDIT3', 'TP53', 'SLC2A4', 'BCL2', 'ACTR2', 'TGFB1', 'PAPOLA', 'NR4A1', 'HSPB2', 'MUC16', 'TBP' | 882 | 15 | 58.13 | 'AGGRESSION', 'DEPRESSION', 'DEPRESSIVE SYMPTOM', 'SUICIDAL BEHAVIOUR', 'SUICIDAL IDEATION' | 57 | 0.42 | 0.32 | -1.25 | -1.69 | 'AMNESIA', 'COGNITIVE DISORDER', 'CONFUSIONAL STATE', 'DELIRIUM', 'DEMENTIA', 'DISORIENTATION', 'DISTURBANCE IN ATTENTION', 'MEMORY IMPAIRMENT', 'MENTAL STATUS CHANGES', 'SEDATION' | 406 | 2.24 | 2.09 | 1.15 | 1 |
| **RANOLAZINE** | CARDIOVASCULAR SYSTEM DRUGS/CARDIAC THERAPY DRUGS/OTHER CARDIAC PREPARATIONS in ATC/Other plain cardiac preparations in ATC' | 'SCN9A', 'SCN10A', 'SCN5A', 'SCN4A' | 875 | 13 | 67.31 | 'AGGRESSION', 'DEPRESSION', 'MAJOR DEPRESSION', 'SUICIDAL IDEATION' | 33 | 0.24 | 0.17 | -2.01 | -2.6 | 'AMNESIA', 'COGNITIVE DISORDER', 'CONFUSIONAL STATE', 'DELIRIUM', 'DEMENTIA', "DEMENTIA ALZHEIMER'S TYPE", 'DISORIENTATION', 'DISTURBANCE IN ATTENTION', 'MEMORY IMPAIRMENT', 'MENTAL STATUS CHANGES', 'SEDATION' | 398 | 2.21 | 2.06 | 1.14 | 0.98 |
| **PEGINTERFERON BETA-1A** | ANTINEOPLASTIC AND IMMUNOMODULATING AGENTS/IMMUNOSTIMULANTS/IMMUNOSTIMULANTS/Interferons' | 'IFNAR2', 'IFNAR1' | 868 | 5 | 173.6 | 'AGGRESSION', 'DEPRESSION', 'MAJOR DEPRESSION', 'SUICIDAL IDEATION' | 124 | 0.92 | 0.78 | -0.11 | -0.41 | 'AMNESIA', 'COGNITIVE DISORDER', 'CONFUSIONAL STATE', 'DELIRIUM', 'DEMENTIA', "DEMENTIA ALZHEIMER'S TYPE", 'DISORIENTATION', 'DISTURBANCE IN ATTENTION', 'MEMORY IMPAIRMENT', 'MENTAL STATUS CHANGES', 'TRANSIENT GLOBAL AMNESIA' | 397 | 2.23 | 2.07 | 1.14 | 0.99 |
| **INDAPAMIDE** | CARDIOVASCULAR SYSTEM DRUGS/DIURETICS/LOW-CEILING DIURETICS, EXCL. THIAZIDES/sulfonamides, low-ceiling diuretics, plain' | 'KCNE1', 'KCNQ1', 'SLC12A3' | 495 | 15 | 33 | 'AGGRESSION', 'DEPRESSION', 'DEPRESSIVE SYMPTOM', 'SUICIDAL IDEATION' | 23 | 0.30 | 0.20 | -1.71 | -2.41 | 'AMNESIA', 'COGNITIVE DISORDER', 'CONFUSIONAL STATE', 'DELIRIUM', 'DEMENTIA', 'DISORIENTATION', 'DISTURBANCE IN ATTENTION', 'MEMORY IMPAIRMENT', 'MENTAL STATUS CHANGES' | 303 | 2.98 | 2.78 | 1.56 | 1.38 |
| **ZICONOTIDE** | NERVOUS SYSTEM DRUGS/ANALGESICS/OTHER ANALGESICS AND ANTIPYRETICS in ATC/Other analgesics and antipyretics in ATC' | 'CACNA1B' | 681 | 16 | 42.56 | 'AGGRESSION', 'DEPRESSION', 'SUICIDAL IDEATION', 'DEPRESSIVE SYMPTOM', 'IMPULSIVE BEHAVIOUR', 'SUICIDAL BEHAVIOUR' | 94 | 0,893037186 | 0,740179229 | -0,162302654 | -0,503947144 | 'COGNITIVE DISORDER', 'CONFUSIONAL STATE', 'MENTAL STATUS CHANGES', 'DISORIENTATION', 'MEMORY IMPAIRMENT', 'AMNESIA', 'DELIRIUM', 'DISTURBANCE IN ATTENTION', 'SEDATION', 'DEMENTIA', "DEMENTIA ALZHEIMER'S TYPE", 'FRONTOTEMPORAL DEMENTIA' | 297 | 2.12 | 1.94 | 1.08 | 0.88 |
| **VANCOMYCIN** | ALIMENTARY TRACT AND METABOLISM DRUGS/ANTIDIARRHEALS, INTESTINAL ANTIINFLAMMATORY/ANTIINFECTIVE AGENTS/INTESTINAL ANTIINFECTIVES/antibiotics, intestinal', 'ANTIINFECTIVES FOR SYSTEMIC USE/ANTIBACTERIALS FOR SYSTEMIC USE/OTHER ANTIBACTERIALS in ATC/Glycopeptide antibacterials' | 'D-Ala-D-Ala moiety of NAM/NAG peptide subunits of peptidoglycan' | 710 | 15 | 47.33 | 'DEPRESSION', 'IMPULSIVE BEHAVIOUR', 'SUICIDAL BEHAVIOUR', 'SUICIDAL IDEATION', 'AGGRESSION', 'ANHEDONIA', 'DEPRESSIVE SYMPTOM' | 44 | 0,400823868 | 0,301060757 | -1,308697238 | -1,810125517 | 'MENTAL STATUS CHANGES', 'AMNESIA', 'COGNITIVE DISORDER', 'CONFUSIONAL STATE', 'DELIRIUM', 'DISORIENTATION', 'DISTURBANCE IN ATTENTION', 'MEMORY IMPAIRMENT', 'SEDATION', "DEMENTIA ALZHEIMER'S TYPE" | 294 | 2.01 | 1.84 | 1.00 | 0.81 |
| **RIFAXIMIN** | ALIMENTARY TRACT AND METABOLISM DRUGS/ANTIDIARRHEALS, INTESTINAL ANTIINFLAMMATORY/ANTIINFECTIVE AGENTS/INTESTINAL ANTIINFECTIVES/antibiotics, intestinal', 'DERMATOLOGICALS/ANTIBIOTICS AND CHEMOTHERAPEUTICS FOR DERMATOLOGICAL USE/ANTIBIOTICS FOR TOPICAL USE/Other antibiotics for topical use in ATC' | 'NR1I2' | 531 | 15 | 35.4 | 'AGGRESSION', 'DEPRESSION', 'SUICIDAL IDEATION' | 19 | 0.23 | 0.14 | -2.08 | -2.85 | 'AMNESIA', 'COGNITIVE DISORDER', 'CONFUSIONAL STATE', 'DELIRIUM', 'DEMENTIA', 'DISORIENTATION', 'DISTURBANCE IN ATTENTION', 'MEMORY IMPAIRMENT', 'MENTAL STATUS CHANGES', 'SEDATION', 'TRANSIENT GLOBAL AMNESIA' | 285 | 2.60 | 2.41 | 1.37 | 1.18 |
| **IFOSFAMIDE** | ANTINEOPLASTIC AND IMMUNOMODULATING AGENTS/ANTINEOPLASTIC AGENTS/ALKYLATING AGENTS/Nitrogen mustard analogues' | 'DNMT1', 'TP53', 'IL6', 'MGMT', 'GSTP1', 'TERC', 'HSPA4' | 441 | 15 | 29.4 | 'AGGRESSION', 'DEPRESSION', 'IMPULSIVE BEHAVIOUR' | 12 | 0.17 | 0.10 | -2.45 | -3.43 | 'COGNITIVE DISORDER', 'CONFUSIONAL STATE', 'DISORIENTATION', 'AMNESIA', 'DELIRIUM', 'DEMENTIA', 'DISTURBANCE IN ATTENTION', 'MEMORY IMPAIRMENT', 'MENTAL STATUS CHANGES', 'SEDATION', 'TRANSIENT GLOBAL AMNESIA' | 259 | 2.85 | 2.64 | 1.50 | 1.30 |
| **ERTAPENEM** | ANTIINFECTIVES FOR SYSTEMIC USE/ANTIBACTERIALS FOR SYSTEMIC USE/OTHER BETA-LACTAM ANTIBACTERIALS in ATC/Carbapenems' | 'mrdA', 'ftsI', 'dacB', 'mrcB', 'dacC' | 385 | 15 | 25.67 | 'AGGRESSION', 'DEPRESSION', 'SUICIDAL IDEATION' | 17 | 0.28 | 0.17 | -1.77 | -2.59 | 'AMNESIA', 'CONFUSIONAL STATE', 'DELIRIUM', 'DISORIENTATION', 'MENTAL STATUS CHANGES', 'COGNITIVE DISORDER', 'DEMENTIA', 'DISTURBANCE IN ATTENTION', 'MEMORY IMPAIRMENT', 'SEDATION' | 260 | 3.28 | 3.06 | 1.70 | 1.50 |
| **FLUDARABINE** | ANTINEOPLASTIC AND IMMUNOMODULATING AGENTS/ANTINEOPLASTIC AGENTS/ANTIMETABOLITES/Purine analogs, antimetabolites antineoplastic' | 'ATM', 'MPO', 'BAX', 'POLA1', 'PIK3CG', 'RRM1', 'RRM2', 'DCK', 'XIAP', 'ADA', 'CD40' | 461 | 15 | 30.73 | 'AGGRESSION', 'DEPRESSION', 'SUICIDAL IDEATION', 'MAJOR DEPRESSION' | 29 | 0.40 | 0.28 | -1.28 | -1.90 | 'COGNITIVE DISORDER', 'AMNESIA', 'CONFUSIONAL STATE', 'DELIRIUM', 'DEMENTIA', "DEMENTIA ALZHEIMER'S TYPE", 'DISORIENTATION', 'DISTURBANCE IN ATTENTION', 'FRONTOTEMPORAL DEMENTIA', 'MEMORY IMPAIRMENT', 'MENTAL STATUS CHANGES', 'SEDATION' | 230 | 2.42 | 2.21 | 1.27 | 1.05 |
| **PIPERACILLIN, TAZOBACTAM DRUG COMBINATION** |  |  | 467 | 15 | 31.13 | 'AGGRESSION', 'DEPRESSION', 'SUICIDAL IDEATION' | 17 | 0.23 | 0.14 | -2.05 | -2.87 | 'CONFUSIONAL STATE', 'DELIRIUM', 'AMNESIA', 'DISTURBANCE IN ATTENTION', 'COGNITIVE DISORDER', 'DEMENTIA', 'DISORIENTATION', 'MEMORY IMPAIRMENT', 'MENTAL STATUS CHANGES', 'SEDATION', "DEMENTIA ALZHEIMER'S TYPE" | 213 | 2.21 | 2.00 | 1.14 | 0.91 |
| **CEFEPIME** | ANTIINFECTIVES FOR SYSTEMIC USE/ANTIBACTERIALS FOR SYSTEMIC USE/OTHER BETA-LACTAM ANTIBACTERIALS in ATC/Fourth-generation cephalosporins | 'IL2' | 355 | 15 | 23.67 | 'AGGRESSION', 'DEPRESSION', 'IMPULSIVE BEHAVIOUR' | 9 | 0.16 | 0.09 | -2.54 | -3.68 | 'AMNESIA', 'COGNITIVE DISORDER', 'CONFUSIONAL STATE', 'DELIRIUM', 'DEMENTIA', 'DISORIENTATION', 'DISTURBANCE IN ATTENTION', 'MEMORY IMPAIRMENT', 'MENTAL STATUS CHANGES', 'SEDATION' | 212 | 2.91 | 2.67 | 1.53 | 1.3 |
| **MELPHALAN** | ANTINEOPLASTIC AND IMMUNOMODULATING AGENTS/ANTINEOPLASTIC AGENTS/ALKYLATING AGENTS/Nitrogen mustard analogues' | 'FANCC', 'PLAT', 'OPLAH', 'CDKN1A', 'MGMT', 'GSTP1', 'IFNG', 'ABCC1', 'ABL1' | 402 | 15 | 26.8 | 'AGGRESSION', 'DEPRESSION', 'MAJOR DEPRESSION', 'SUICIDAL BEHAVIOUR' | 30 | 0.48 | 0.34 | -1.03 | -1.64 | 'AMNESIA', 'COGNITIVE DISORDER', 'CONFUSIONAL STATE', 'DELIRIUM', 'DEMENTIA', 'DISORIENTATION', 'DISTURBANCE IN ATTENTION', 'MEMORY IMPAIRMENT', 'MENTAL STATUS CHANGES', 'SEDATION' | 189 | 2.28 | 2.06 | 1.18 | 0.94 |
| **BICALUTAMIDE** | ANTINEOPLASTIC AND IMMUNOMODULATING AGENTS/ENDOCRINE THERAPY ANTINEOPLASTIC AND IMMUNOMODULATING AGENTS/HORMONE ANTAGONISTS AND RELATED AGENTS/Anti-androgen hormone antagonists and related agents | 'BAX', 'KMT2D', 'VDR', 'CYP2B6', 'FST', 'CDH1', 'AR', 'KLK3', 'CFLAR' | 377 | 15 | 25.13 | 'AGGRESSION', 'DEPRESSION', 'DEPRESSIVE SYMPTOM', 'SUICIDAL IDEATION' | 54 | 0.93 | 0.72 | -0.11 | -0.56 | 'AMNESIA', 'COGNITIVE DISORDER', 'CONFUSIONAL STATE', 'DELIRIUM', 'DEMENTIA', "DEMENTIA ALZHEIMER'S TYPE", 'DISORIENTATION', 'DISTURBANCE IN ATTENTION', 'MEMORY IMPAIRMENT', 'MENTAL STATUS CHANGES', 'SEDATION' | 171 | 2.21 | 1.97 | 1.14 | 0.88 |
| **AZACITIDINE** | ANTINEOPLASTIC AND IMMUNOMODULATING AGENTS/ANTINEOPLASTIC AGENTS/ANTIMETABOLITES/Pyrimidine analogues, antineoplastic antimetabolites | 'GATA2', 'GSTP1', 'TET2', 'IL11', 'TAGLN', 'TG', 'GGT1', 'MGMT', 'ASXL1', 'CD247', 'FLT3', 'MYC', 'TRIT1', 'PLAU', 'AFP', 'DNMT1', 'LIF', 'DNMT3A', 'ABL1', 'IDH1' | 311 | 15 | 20.73 | 'AGGRESSION', 'DEPRESSION', 'DEPRESSIVE SYMPTOM', 'SUICIDAL BEHAVIOUR', 'SUICIDAL IDEATION' | 32 | 0.67 | 0.48 | -0.58 | -1.17 | 'AMNESIA', 'COGNITIVE DISORDER', 'CONFUSIONAL STATE', 'DELIRIUM', 'DEMENTIA', "DEMENTIA ALZHEIMER'S TYPE", 'DISORIENTATION', 'FRONTOTEMPORAL DEMENTIA', 'MEMORY IMPAIRMENT', 'MENTAL STATUS CHANGES' | 155 | 2.42 | 2.17 | 1.27 | 1 |
| **MEGESTROL** | GENITO URINARY SYSTEM AND SEX HORMONES/SEX HORMONES AND MODULATORS OF THE GENITAL SYSTEM/HORMONAL CONTRACEPTIVES FOR SYSTEMIC USE/Progestogen systemic hormonal contraceptives', 'GENITO URINARY SYSTEM AND SEX HORMONES/SEX HORMONES AND MODULATORS OF THE GENITAL SYSTEM/PROGESTOGEN SEX HORMONES AND MODULATORS OF THE GENITAL SYSTEM/Pregnadien derivatives, progestogens', 'ANTINEOPLASTIC AND IMMUNOMODULATING AGENTS/ENDOCRINE THERAPY ANTINEOPLASTIC AND IMMUNOMODULATING AGENTS/HORMONES AND RELATED AGENTS/Progestogens, endocrine therapy drugs' | 'PGR' | 229 | 15 | 15.27 | 'AGGRESSION', 'ANHEDONIA', 'DEPRESSION' | 12 | 0.33 | 0.19 | -1.52 | -2.50 | 'CONFUSIONAL STATE', "DEMENTIA ALZHEIMER'S TYPE", 'AMNESIA', 'COGNITIVE DISORDER', 'DELIRIUM', 'DEMENTIA', 'DISORIENTATION', 'DISTURBANCE IN ATTENTION', 'MEMORY IMPAIRMENT', 'MENTAL STATUS CHANGES', 'SEDATION' | 148 | 3.14 | 2.85 | 1.64 | 1.36 |
| **LACTULOSE** | ALIMENTARY TRACT AND METABOLISM DRUGS/DRUGS FOR CONSTIPATION/DRUGS FOR CONSTIPATION/Osmotically acting laxatives' | 'CSF2', 'TNF', 'FCGR3A', 'FCGR3B' | 234 | 15 | 15.6 | 'AGGRESSION', 'DEPRESSION', 'DEPRESSION SUICIDAL' | 13 | 0.35 | 0.21 | -1.44 | -2.37 | 'CONFUSIONAL STATE', 'AMNESIA', 'COGNITIVE DISORDER', 'DELIRIUM', 'DEMENTIA', 'DISORIENTATION', 'DISTURBANCE IN ATTENTION', 'MEMORY IMPAIRMENT', 'MENTAL STATUS CHANGES', 'SEDATION' | 146 | 3.03 | 2.74 | 1.59 | 1.31 |
| **BENDROFLUMETHIAZIDE** | CARDIOVASCULAR SYSTEM DRUGS/DIURETICS/LOW-CEILING DIURETICS, THIAZIDES/Thiazides, plain | 'KCNN2', 'SLC12A3', 'KCNN1', 'SLC12A1', 'SLC12A3' | 246 | 15 | 16.4 | ['AGGRESSION', 'DEPRESSION', 'DEPRESSION SUICIDAL', 'SUICIDAL IDEATION'] | 13 | 0.34 | 0.2 | -1.51 | -2.45 | ['CONFUSIONAL STATE', 'DISORIENTATION', 'AMNESIA', 'DELIRIUM', 'DEMENTIA', 'DISTURBANCE IN ATTENTION', 'MEMORY IMPAIRMENT', 'SEDATION'] | 143 | 2.83 | 2.54 | 1.49 | 1.21 |
| **DAUNORUBICIN** | ANTINEOPLASTIC AND IMMUNOMODULATING AGENTS/ANTINEOPLASTIC AGENTS/CYTOTOXIC ANTIBIOTICS AND RELATED SUBSTANCES/Anthracyclines and related substances' | 'GSTP1', 'KMT2A', 'GATA1', 'CYP1A1', 'TYMS', 'WT1', 'FLT3', 'DNMT3A', 'TOP2B', 'ABCB1', 'FAS', 'RPSA', 'NPM1', 'ANXA5', 'TP53', 'ABCC1', 'CDK2', 'APP', 'TOP2A' | 243 | 15 | 16.2 | 'DEPRESSION', 'AGGRESSION' | 11 | 0.29 | 0.16 | -1.72 | -2.75 | 'CONFUSIONAL STATE', 'MENTAL STATUS CHANGES', 'COGNITIVE DISORDER', 'DELIRIUM', 'DISORIENTATION', 'DISTURBANCE IN ATTENTION', 'MEMORY IMPAIRMENT', 'AMNESIA', 'SEDATION' | 124 | 2.48 | 2.19 | 1.30 | 1.00 |
| **BLINATUMOMAB** | ANTINEOPLASTIC AND IMMUNOMODULATING AGENTS/ANTINEOPLASTIC AGENTS/OTHER ANTINEOPLASTIC AGENTS in ATC/Monoclonal antibodies, antineoplastic | 'CD3E', 'CD3D', 'CD3G', 'BCR', 'CD19', 'ABL1', 'CD247' | 232 | 5 | 46.4 | 'AGGRESSION', 'DEPRESSION', 'DEPRESSIVE SYMPTOM', 'MAJOR DEPRESSION' | 13 | 0.36 | 0.21 | -1.43 | -2.37 | 'AMNESIA', 'COGNITIVE DISORDER', 'CONFUSIONAL STATE', 'DELIRIUM', 'DISORIENTATION', 'DISTURBANCE IN ATTENTION', 'MEMORY IMPAIRMENT', 'MENTAL STATUS CHANGES', 'SEDATION' | 119 | 2.49 | 2.2 | 1.31 | 1.01 |
| **CLOFARABINE** | ANTINEOPLASTIC AND IMMUNOMODULATING AGENTS/ANTINEOPLASTIC AGENTS/ANTIMETABOLITES/Purine analogs, antimetabolites antineoplastic | 'RRM2', 'POLE', 'RRM2B', 'POLD1', 'POLA1', 'RRM1', 'FLT3' | 195 | 13 | 15 | 'DEPRESSION', 'AGGRESSION' | 3 | 0.1 | 0.03 | -3.13 | -5.2 | 'AMNESIA', 'COGNITIVE DISORDER', 'CONFUSIONAL STATE', 'DELIRIUM', 'DEMENTIA', 'DISORIENTATION', 'FRONTOTEMPORAL DEMENTIA', 'MEMORY IMPAIRMENT', 'MENTAL STATUS CHANGES', 'SEDATION' | 114 | 2.84 | 2.53 | 1.5 | 1.19 |
| **TRIPTORELIN PAMOATE** | ANTINEOPLASTIC AND IMMUNOMODULATING AGENTS/ENDOCRINE THERAPY ANTINEOPLASTIC AND IMMUNOMODULATING AGENTS/HORMONES AND RELATED AGENTS/Gonadotropin releasing hormone analogues, endocrine therapy drugs' | 'GNRHR', 'GNRHR2', 'STS' | 183 | 15 | 12.2 | 'DEPRESSION', 'SUICIDAL IDEATION', 'DEPRESSION SUICIDAL', 'ANHEDONIA', 'MAJOR DEPRESSION' | 25 | 0.88 | 0.61 | -0.17 | -0.84 | 'AMNESIA', 'COGNITIVE DISORDER', 'CONFUSIONAL STATE', 'DEMENTIA', "DEMENTIA ALZHEIMER'S TYPE", 'MEMORY IMPAIRMENT', 'DISTURBANCE IN ATTENTION' | 114 | 3.02 | 2.70 | 1.58 | 1.27 |
| **CYPROTERONE** | GENITO URINARY SYSTEM AND SEX HORMONES/SEX HORMONES AND MODULATORS OF THE GENITAL SYSTEM/ANTIANDROGEN SEX HORMONES AND MODULATORS OF THE GENITAL SYSTEM/Antiandrogens, plain | 'AR' | 127 | 9 | 14.11 | 'DEPRESSION', 'SUICIDAL IDEATION', 'AGGRESSION' | 9 | 0.46 | 0.24 | -1.08 | -2.22 | 'DEMENTIA ALZHEIMER'S TYPE', 'AMNESIA', 'FRONTOTEMPORAL DEMENTIA', 'CONFUSIONAL STATE' | 104 | 3.98 | 3.67 | 1.97 | 1.65 |
| **MESNA** | RESPIRATORY SYSTEM DRUGS/COUGH AND COLD PREPARATIONS/EXPECTORANTS, EXCL. COMBINATIONS WITH COUGH SUPPRESSANTS/Mucolytics', 'VARIOUS DRUG CLASSES IN ATC/ALL OTHER THERAPEUTIC PRODUCTS/ALL OTHER THERAPEUTIC PRODUCTS/Detoxifying agents for antineoplastic treatment' |  | 178 | 15 | 11.87 | 'DEPRESSION' | 3 | 0.10 | 0.03 | -3.00 | -5.06 | 'AMNESIA', 'COGNITIVE DISORDER', 'CONFUSIONAL STATE', 'DELIRIUM', 'DISORIENTATION', 'DISTURBANCE IN ATTENTION', 'MENTAL STATUS CHANGES' | 101 | 2.75 | 2.42 | 1.45 | 1.12 |
| **ASPIRIN, DIPYRIDAMOLE DRUG COMBINATION** |  |  | 242 | 15 | 16.13 | 'DEPRESSION', 'SUICIDAL IDEATION', 'AGGRESSION' | 23 | 0.61 | 0.42 | -0.69 | -1.39 | 'DEMENTIA', 'AMNESIA', 'COGNITIVE DISORDER', "DEMENTIA ALZHEIMER'S TYPE", 'DISORIENTATION', 'MEMORY IMPAIRMENT', 'SEDATION', 'CONFUSIONAL STATE', 'DELIRIUM', 'DISTURBANCE IN ATTENTION', 'MENTAL STATUS CHANGES' | 102 | 2.05 | 1.77 | 1.03 | 0.7 |
| **TOPOTECAN** | ANTINEOPLASTIC AND IMMUNOMODULATING AGENTS/ANTINEOPLASTIC AGENTS/OTHER ANTINEOPLASTIC AGENTS in ATC/Other antineoplastic agents in ATC' | 'TOP1', 'ALK', 'BDNF', 'ATRX', 'TYMS', 'MUC16', 'TOP1MT', 'PTEN', 'RB1', 'TP53', 'PIK3CA' | 203 | 15 | 13.53 | 'AGGRESSION', 'ANHEDONIA', 'DEPRESSION' | 10 | 0.31 | 0.17 | -1.60 | -2.67 | 'AMNESIA', 'COGNITIVE DISORDER', 'CONFUSIONAL STATE', 'DELIRIUM', 'DISORIENTATION', 'DISTURBANCE IN ATTENTION', 'MEMORY IMPAIRMENT', 'MENTAL STATUS CHANGES', 'SEDATION' | 100 | 2.39 | 2.08 | 1.24 | 0.91 |
