# Supplementary material for: Molecular basis of mood and cognitive adverse events elucidated via a combination of pharmacovigilance data mining and functional enrichment analysis
Source: Arch Toxicol. 2020 Jun 5;94(8):2829–45. doi: 10.1007/s00204-020-02788-1 (PMC7395038; doi:10.1007/s00204-020-02788-1)
Supplement: Supplementary file 2 — Supplementary file2 (DOCX 90 kb) [file 204_2020_2788_MOESM2_ESM.docx]

Online Resource 2

**Molecular basis of mood and cognitive adverse events elucidated via a combination of pharmacovigilance data mining and functional enrichment analysis**

Christos Andronis^1,*^, João Pedro Silva^2,*^, Eftychia Lekka^1^, Vassilis Virvilis^1^, Helena Carmo^2^, Konstantina Bampali^3^, Margot Ernst^3^, Yang Hu^4^, Irena Loryan^4^, Jacques Richard^5^, Félix Carvalho^2,#^, Miroslav M. Savić^6,#^

^1^Biovista, 34 Rodopoleos Street, 16777 Athens, Greece

^2^UCIBIO, REQUIMTE, Laboratory of Toxicology, Department of Biological Sciences, Faculty of Pharmacy, University of Porto, 4050-313, Porto, Portugal

^3^Department of Molecular Neurosciences, Medical University of Vienna, Spitalgasse 4, A-1090 Vienna, Austria

^4^Translational PKPD group, Department of Pharmaceutical Biosciences, Associate member of SciLifeLab, Uppsala University, Sweden

^5^Sanofi R&D, 371 avenue Professeur Blayac, Montpellier, 34000 France

^6^Department of Pharmacology, Faculty of Pharmacy, University of Belgrade, Vojvode Stepe 450, 11000 Belgrade, Serbia

*The authors contributed equally to the manuscript.

#Corresponding authors:

Félix Carvalho, UCIBIO, REQUIMTE, Laboratory of Toxicology, Faculty of Pharmacy, University of Porto, Portugal, Tel. +351 220428600, E-mail: felixdc@ff.up.pt; Miroslav Savić, Faculty of Pharmacy, University of Belgrade, Serbia, Tel. +381 113951280, E-mail: miroslav@pharmacy.bg.ac.rs

**Supplementary Table 2** – Candidate pathway elements (CPEs) associated with the pharmaceuticals with higher occurrence of mood and cognitive AEs. For each CPE, their respective encoding genes, pathways in which they are involved, sites of expression, and associated pharmaceuticals are provided. In addition, the targets related to immune-modulation were highlighted.

| **Candidate pathway element (CPE)** | **Encoding gene** | **Pathways in which CPE is involved** | **References** | **Sites of CPE (protein) expression (divided by score)** | | | | **Pharmaceuticals that modulate this CPE (examples)** | **CPE association with immune function** |
| --- | --- | --- | --- | --- | --- | --- | --- | --- | --- |
|  |  |  |  | **Low** | **Medium** | **High** | **Unknown score** |  |  |
| ABL Proto-Oncogene 1, Non-Receptor Tyrosine Kinase | ABL1 | Myogenesis, TP53 Network, DNA IR-Double Strand Breaks (DSBs) and cellular response via ATM, Retinoblastoma Gene in Cancer, Signaling by ROBO receptors, DNA Damage Response, DNA Damage Response (only ATM dependent), miRNAs involved in DNA damage response, miRNA Regulation of DNA Damage Response, ErbB Signaling Pathway, Ras Signaling, Cell Cycle, RHO GTPases Activate WASPs and WAVEs, EGF/EGFR Signaling Pathway, DNA Double Strand Break Response , Fcgamma receptor (FCGR) dependent phagocytosis | 1 | Cerebral cortex, Hippocampus, Caudate, Cerebellum, Parathyroid gland, Appendix, Bone marrow, Lymph node, Smooth muscle, Pancreas, Oral mucosa, Esophagus, Prostate, Ovary, Soft tissue, Skin | Thyroid gland, Adrenal gland, Tonsil, Spleen, Heart muscle, Skeletal muscle, Lung, Nasopharynx, Bronchus, Liver, Gallbladder, Salivary gland, Stomach, Duodenum, Small intestine, Colon, Rectum, Kidney, Urinary bladder, Testis, Epididymis, Seminal vesicle, Fallopian tube, Breast, Vagina, Cervix (uterine), Endometrium, Placenta | .. | .. | Azacitidine, Melphalan, Blinatumomab | YES |
| Acetylcholinesterase | ACHE | Acetylcholine Synthesis, Neurotransmitter clearance, Biogenic Amine Synthesis, Monoamine Transport, Melatonin metabolism and effects, Synthesis, secretion, and deacylation of Ghrelin, Glycerophospholipid biosynthesis | 2 | .. | .. | .. | B Cell, Blood, Brain, Liver, Muscle, Osteoblast, Retina, Thalamus | Rivastigimine | YES |
| Amyloid Beta Precursor Protein | APP | Amyloid fiber formation, Advanced glycosylation endproduct receptor signaling, TAK1 activates NFKB by phosphorylation and activation of IKKs complex, Copper homeostasis, Apoptosis-related network due to altered Notch3 in ovarian cancer, Deregulated CDK5 triggers multiple neurodegenerative pathways in Alzheimer's disease models, trans-Golgi network vesicle budding, Post-translational protein phosphorylation, Alzheimers disease, nucleotide-binding domain, leucine rich repeat containing receptor (NLR) signaling pathways, Response to elevated platelet cytosolic Ca2+, Regulation of insulin-like growth factor (IGF) transport and uptake by insulin-like growth factor binding proteins (IGFBPs), Fragile X syndrome, Endoderm differentiation, TGF-beta signaling | 3 | Duodenum, Colon, Rectum, Gallbladder, Pancreas, Testis, Epididymis, Seminal vesicle, Prostate, Endometrium, Placenta, Breast, Appendix | Cerebral cortex, Cerebellum, Hippocampus, Caudate | .. | .. | Daunorubicin | YES |
| Anaplastic Lymphoma Kinase | ALK | MET in type 1 papillary renal cell carcinoma; Non-small cell lung cancer; Differentiation Pathway | 4 | Thyroid gland, Parathyroid gland, Adrenal gland, Nasopharynx, Bronchus, Lung, Oral mucosa, Colon, Rectum, Gallbladder, Urinary bladder, Testis, Epididymis, Seminal vesicle, Vagina, Ovary, Fallopian tube, Endometrium, Uterine Cervix, Placenta, Heart muscle, Skeletal muscle, Lymph node, Bone marrow, Soft tissue | Caudate, Skin | Cerebral cortex, Cerebellum, Hippocampus | .. | Topotecan | YES |
| Androgen receptor | AR | Transcriptional regulation by RUNX2; Activated PKN1 stimulates transcription of AR (androgen receptor) regulated genes KLK2 and KLK3; HSP90 chaperone cycle for steroid hormone receptors (SHR); Generic Transcription Pathway; SUMOylation of intracellular receptors; Deubiquitination | 5-7 | Uterine cervix | Testis, Fallopian tube, Breast, Endometrium | Epididymis, Seminal vesicle | .. | Bicalutamide, Cyproterone | YES |
| ATM serine/threonine kinase | ATM | DNA Double Strand Break Response; DNA Damage/Telomere Stress Induced Senescence; TP53 Regulates Transcription of DNA Repair Genes; Meiotic recombination; Cellular response to heat stress; mTOR signaling; Alanine and aspartate metabolism; G1 to S cell cycle control; Integrated Breast Cancer Pathway, ATM Signaling Pathway | 8 | Caudate, ovary | Cerebral cortex, Hippocampus, cerebellum, apendix, heart, liver, salivary gland, vagina, soft tissue | Endocrine tissues, immune system tissues (except tonsil), smooth muscle, lung, bronchus, gallbladder, pancreas, gastrointestinal tract, kidney, urinary bladder, female tissues, skin | .. | Fludarabine, Temozolomide | YES |
| ATP Binding Cassette Subfamily C Member 1 | ABCC1 | Metabolism of water-soluble vitamins and cofactors, ABC-family proteins mediated transport, Arachidonic acid metabolism, Irinotecan Pathway | 9 | Bone marrow, Lung, Nasopharynx, Gallbladder, Pancreas, Fallopian tube, Skin, Cerebral cortex, Colon | Appendix, Tonsil, Duodenum, Small intestine, Rectum, Kidney, Testis, Endometrium, Ovary, Soft tissue | .. | .. | Etoposide, Daunorubicin, Melphalan | YES |
| ATR Serine/Threonine Kinase | ATR | Cell Cycle Checkpoints; HDR through Homologous Recombination (HRR) or Single Strand Annealing (SSA); Fanconi Anemia Pathway; TP53 Regulates Transcription of DNA Repair Genes; Meiotic synapsis; Cellular response to heat stress; Retinoid metabolism and transport; Wax biosynthesis; Visual phototransduction; Integrated Breast Cancer Pathway; Triglyceride metabolism | 10 | .. | .. | .. | Brain, colon, heart, leukocyte, liver, lung, ovary, pancreas, placenta, prostate, skeletal muscle, small intestine, spleen, testis | Temozolomide | YES |
| ATRX Chromatin Remodeler | ATRX | Pathways Affected in Adenoid Cystic Carcinoma, miR-targeted genes in squamous cell - TarBase, miR-targeted genes in epithelium - TarBase, miR-targeted genes in muscle cell - TarBase, miR-targeted genes in lymphocytes - TarBase | 11 | Parathyroid gland, Liver | Lymph node, Prostate, Seminal vesicle, Cervix (uterine), Endometrium | Cerebral cortex, Hippocampus, Caudate, Cerebellum, Thyroid gland, Adrenal gland, Appendix, Bone marrow, Tonsil, Spleen, Heart muscle, Skeletal muscle, Smooth muscle, Lung, Nasopharynx, Bronchus, Gallbladder, Pancreas, Salivary gland, Oral mucosa, Esophagus, Stomach, Duodenum, Small intestine, Colon, Rectum, Kidney, Urinary bladder, Testis, Epididymis, Fallopian tube, Breast, Vagina, Ovary, Placenta, Soft tissue, Skin | .. | Temozolomide | .. |
| B-cell Activating Factor a.k.a TNF Superfamily Member 13b | BAFF (a.k.a. TNFSF13B) | TNFR2 non-canonical NF-kB pathway | 12 | Liver, Duodenum, Colon | Cerebral cortex, Hippocampus, Caudate ,Cerebellum, Thyroid gland, Parathyroid gland, Adrenal gland, Heart muscle, Skeletal muscle, Smooth muscle, Lung, Nasopharynx, Bronchus, Gallbladder, Pancreas, Salivary gland, Oral mucosa, Esophagus, Stomach, Small intestine, Rectum, Kidney, Urinary bladder, Prostate, Epididymis, Seminal vesicle, Fallopian tube, Breast, Vagina, Cervix (uterine), Endometrium, Ovary, Placenta, Soft tissue, Skin | Appendix, Bone marrow, Lymph node, Tonsil, Spleen, Testis | .. | Etoposide | .. |
| B-cell lymphoma-2 (Bcl-2) | BCL2 | Intrinsic Pathway for Apoptosis, Nucleotide-binding domain, leucine rich repeat containing receptor (NLR) signaling pathways, Non-genomic estrogen signaling, ESR-mediated signaling | 13 | Gallbladder, pancreas, esophagus, duodenum, Small Intestine | Parathyroid gland, Smooth muscle, Colon, Rectum, Kidney, Urinary bladder, Breast, Vagina, Uterine Cervix, Soft tissue | Thyroid gland, Appendix, Bone marrow, Lymph node, Tonsil, Spleen, lung, Prostate, epididymis, seminal vesicle, Fallopian tube, endometrium, ovary, placenta, skin | .. | Etoposide, Isotretinoin | YES |
| Bcl-2-associated X (Bax) | BAX | TP53 Regulates Transcription of Cell Death Genes, Intrinsic Pathway for Apoptosis, TP53 Regulates Transcription of Cell Cycle Genes, Transcriptional regulation by RUNX2, Signaling by NTRK3 (TRKC) | 14 | Skeletal muscle, smooth muscle, vagina, ovary, soft tissue | Cerebral cortex, hippocampus, caudate, cerebellum, Bone marrow, Lymph node, Tonsil, Spleen, heart muscle, liver, salivary gland, oral mucosa, Esophagus, epididymis, Seminal Vesicle, fallopian Tube | Thyroid gland, Parathyroid gland, Adrenal gland, appendix, Lung, Gallbladder, Pancreas, Gastrointestinal tract, kidney, urinary bladder, testis, prostate, Breast, Uterine cervix, endometrium, placenta, skin | .. | Rofecoxib, Interferon alpha-2, medroxyprogesterone acetate, Bicalutamide, Etoposide, Fludarabine | YES |
| Bradykinin Receptor B1 | BDKRB1 | ACE inhibitor pathway, Dengue-2 interactions with complement and coagulation cascades, Peptide GPCRs, Complement and coagulation cascades, Vitamin D receptor pathway, Regulation of actin cytoskeleton, G alpha (q) signaling events, G alpha (i) signaling events, GPCRs, Class A rhodopsin-like, Class A/1 (rhodopsin-like receptors) | 15 | Bronchus, Spleen, Lymph node | Cerebellum, Hippocampus, Thyroid gland, Adrenal gland, Oral mucosa, Stomach, Duodenum, Kidney, Testis, Epididymi, Seminal vesicle, Prostate, Ovary, Fallopian tube, Endometrium, Cervix, uterine, Adipose tissue, Skin, Tonsil | Cerebral cortex, Caudate, Parathyroid gland, Nasopharynx, Lung, Salivary gland, Small intestine, Colon, Rectum, Liver, Gallbladder, Pancreas, Urinary bladder, Placenta, Breast, Appendix | Soft tissue not detected | Meflloquine | yes |
| Brain-derived neurotrophic factor | BDNF | Signaling by NTRK2 (TRKB), Transcriptional Regulation by MECP2, BDNF-TrkB Signaling, Neural Crest Cell Migration during Development, HDAC6 interactions, Synaptic signaling pathways associated with autism spectrum disorder, MAPK Signaling Pathway, PI3K-Akt Signaling Pathway | 16 | Thyroid gland, Parathyroid gland, Adrenal gland, Testis, Fallopian tube | Cerebral cortex, Hippocampus, Caudate, Cerebellum | .. | .. | Topotecan | YES |
| Breakpoint cluster region | BCR | Signaling by the B Cell Receptor (BCR), Overview of leukocyte-intrinsic Hippo pathway functions, Signaling by Rho GTPases, DNA Damage Response, Signaling by FGFR1, PI3K-Akt Signaling Pathway, PIP3 activates AKT signaling, RAF/MAPK cascade | 17 | Bone marrow, Lymph node, Skeletal muscle, Ovary | Cerebral cortex, Hippocampus, Caudate, Parathyroid gland, Adrenal gland, Appendix, Tonsil, Heart muscle, Lung, Liver, Gallbladder, Pancreas, Salivary gland, Oral mucosa, Stomach, Duodenum, Small intestine, Colon, Rectum, Kidney, Testis, Prostate, Epididymis, Seminal vesicle, Fallopian tube, Vagina, Cervix (uterine), Endometrium, Soft tissue, Skin | .. | .. | Blinatumomab | .. |
| Breast cancer type 1 susceptibility potein | BRCA1 | DNA Double Strand Break Response, Deubiquitination, TP53 Regulates Transcription of DNA Repair Genes, Meiotic synapsis, Meiotic recombination, Transcriptional Regulation by E2F6, SUMOylation of DNA damage response and repair proteins, Signaling Pathways in Glioblastoma, ATM Signaling Pathway, Androgen receptor signaling pathway, PI3K-Akt Signaling Pathway, DNA IR-Double Strand Breaks (DSBs) and cellular response via ATM, DNA IR-damage and cellular response via ATR | 18 | Hippocampus, spleen, Heart muscle, Liver, Salivary Gland, Soft tissue | Cerebral cortex, caudate, cerebellum, Thyroid, Adrenal gland, Appendix, Bone marrow, Tonsil, Lung, Nasopharynx, Bronchus, Gallbladder, Pancreas, Oral Mucosa, Esophagus, Stomach, Duodenum, Small Intestine, Colon, Rectum, Kidney, Urinary bladder, testis, epididymis, prostate, seminal vesicle, Fallopian tube, ovary, endometrium, placenta, breast, uterine cervix, vagina | Lymph Node, Skin | .. | Temozolomide | YES |
| Breast cancer type 2 susceptibility potein | BRCA2 | HDR through Homologous Recombination (HRR) or Single Strand Annealing (SSA), Meiotic recombination, Homologous recombination, Signaling Pathways in Glioblastoma, DNA IR-Double Strand Breaks (DSBs) and cellular response via ATM, DNA IR-damage and cellular response via ATR | 19 | .. | .. | .. | Mammary gland, serum | Etoposide | YES |
| Butyrylcholinesterase | BCHE | Neurotransmitter clearance, Synthesis, secretion, and deacylation of Ghrelin, Glycerophospholipid biosynthesis, Irinotecan Pathway | 20, 21 | Hippocampus, Thyroid gland, Parathyroid gland, Adrenal gland, Appendix, Bone marrow, Tonsil, Spleen, Heart muscle, Skeletal muscle, Smooth muscle, Lung, Nasopharynx, Bronchus, Gallbladder, Pancreas, Salivary gland, Oral mucosa, Esophagus, Stomach, Duodenum, Small intestine, Colon, Rectum, Kidney, Urinary bladder, Testis, Seminal vesicle, Breast, Vagina, Cervix (uterine), Ovary, Placenta, Soft tissue | Cerebral cortex, Caudate, Cerebellum, Lymph node, Liver, Prostate, Epididymis, Fallopian tube, Endometrium, Skin | .. | .. | Rivastigimine | .. |
| c-MYC proto-oncogene | MYC | PI3K/AKT/mTOR - VitD3 Signalling, let-7 inhibition of ES cell reprogramming, Regulation of Apoptosis by Parathyroid Hormone-related Protein, Metastatic brain tumor, p38 MAPK Signaling Pathway, TP53 Network, Insulin-like Growth Factor-2 mRNA Binding Proteins (IGF2BPs/IMPs/VICKZs) bind RNA, PPAR Alpha Pathway, Structural Pathway of Interleukin 1 (IL-1), ESR-mediated signaling, Transcriptional regulation by the AP-2 (TFAP2) family of transcription factors, MAPK6/MAPK4 signaling, RAC1/PAK1/p38/MMP2 Pathway, DNA Damage Response (only ATM dependent), miRNAs involved in DNA damage response, ErbB Signaling Pathway, Degradation of beta-catenin by the destruction complex, Transcriptional regulation by RUNX3, Association Between Physico-Chemical Features and Toxicity Associated Pathways, Notch Signaling Pathway, Apoptosis, Wnt Signaling Pathway and Pluripotency, Neural Crest Differentiation, Wnt Signaling Pathway, IL-5 Signaling Pathway, G1 to S cell cycle control, IL-7 Signaling Pathway, Hippo-Merlin Signaling Dysregulation, Thymic Stromal LymphoPoietin (TSLP) Signaling Pathway, TCF dependent signaling in response to WNT, TGF-beta Signaling Pathway, Vitamin D Receptor Pathway, Wnt Signaling, DNA Damage Response, Aryl Hydrocarbon Receptor, Prolactin Signaling Pathway, Human Thyroid Stimulating Hormone (TSH) signaling pathway, IL-2 Signaling Pathway, miRNA Regulation of DNA Damage Response, Cell Cycle, Transcriptional activity of SMAD2/SMAD3:SMAD4 heterotrimer, S Phase, Retinoblastoma Gene in Cancer, JAK/STAT, ESC Pluripotency Pathways, Beta-catenin independent WNT signaling, Mitotic G1-G1/S phases, MAPK Signaling Pathway, Signaling by NOTCH1, Interleukin-4 and Interleukin-13 signaling, PI3K-Akt Signaling Pathway, Deubiquitination, Nuclear Receptors Meta-Pathway | 22 | Thyroid gland, Parathyroid gland, Bone marrow, Smooth muscle, Salivary gland, Colon, Fallopian tube, Ovary, Soft tissue | Cerebral cortex, Adrenal gland, Appendix, Lymph node, Tonsil, Spleen, Heart muscle, Skeletal muscle, Nasopharynx, Bronchus, Liver, Gallbladder, Pancreas, Oral mucosa, Esophagus, Stomach, Duodenum, Small intestine, Rectum, Kidney, Urinary bladder, Prostate, Epididymis, Seminal vesicle, Breast, Vagina, Endometrium, Placenta, Skin | Hippocampus, Caudate, Cerebellum, Lung, Testis, Cervix (uterine) | .. | Temozolomide, Azacitidine | YES |
| Cadherin-1 (a.k.a. CAM 120/80 or epithelial cadherin (E-cadherin)) | CDH1 | Integrin cell surface interactions, Tetrahydrobiopterin (BH4) synthesis, recycling, salvage and regulation, Metabolism of nitric oxide, Melatonin metabolism and effects, Common Pathways Underlying Drug Addiction, Calcium Regulation in the Cardiac Cell, Endothelin Pathways, Beta-catenin independent WNT signaling, Signaling by VEGF, C-type lectin receptors, Fcgamma receptor (FCGR) dependent phagocytosis, Cell surface interactions at the vascular wall, Fc epsilon receptor (FCERI) signaling, Complement cascade, Opioid Signalling, Binding and Uptake of Ligands by Scavenger Receptors, | 23 | .. | BronchusLiver | Thyroid gland, Parathyroid gland, Appendix, Tonsil, Lung, Nasopharynx, Gallbladder, Pancreas, Salivary gland, Oral mucosa, Esophagus, Stomach, Duodenum, Small intestine, Colon, Rectum, Kidney, Urinary bladder, Prostate, Epididymis, Seminal vesicle, Fallopian tube, Breast, Vagina, Cervix (uterine), Endometrium, Placenta, Skin | .. | Bicalutamide | YES |
| Cholinergic Receptors (Muscarinic) | CHRM1 | G alpha (q) signalling events, Class A/1 (Rhodopsin-like receptors) | 24 | Kidney, testis, colon | Cerebral cortex, hippocampus, cerebellum | .. | .. | Metoclopramide | YES |
| Cholinergic Receptors (Nicotinic) | CHRNA4 | Acetylcholine binding and downstream events; Nicotine Activity on Dopaminergic Neurons | 25 | .. | .. | .. | Nervous system | Varenicicline, Scopolamine | YES |
| Cholinergic Receptors (Nicotinic) | CHRNA7 | Acetylcholine binding and downstream events, Phosphodiesterases in neuronal function | 26 | Bone marrow, Spleen, Smooth muscle, Liver, Gallbladder, Pancreas, Oral mucosa, Urinary bladder, Prostate, Vagina | Cerebral cortex, Hippocampus, Caudate, Cerebellum, Thyroid gland, Appendix, Lymph node, Tonsil, Heart muscle, Skeletal muscle, Nasopharynx, Bronchus, Salivary gland, Esophagus, Stomach, Duodenum, Small intestine, Colon, Rectum, Kidney, Testis, Epididymis, Seminal vesicle, Fallopian tube, Breast, Cervix (uterine), Endometrium, Placenta, Skin | Parathyroid gland, Adrenal gland, Ovary | .. | Varenicline | YES |
| Cholinergic Receptors (Nicotinic) | CHRNB2 | Acetylcholine binding and downstream events; Nicotine Activity on Dopaminergic Neurons, circadian rhythm signaling, sleep regulation | 25 | .. | .. | .. | Nervous system | Varenicline | YES |
| Cluster of Differentiation 40 (a.k.a. Tumor Necrosis Factor Receptor Superfamily Member 5) | CD40 | Platelet-mediated interactions with vascular and circulating cells, Inflammatory response pathway, TNFR2 non-canonical NfkappaB pathway, Non-genomic actions of 1,25 dihydroxyvitamin D3, Allogrft rejection, Vitamin D receptor pathway, Toll-like receptor signaling pathway, Human complement system, Regulation of toll-like receptor signaling pathway, Immunoreglatory interactions between a Lumphoid and a non-lymphoid cell | 27, 28 | Lung, Appendix | .. | Spleen, Lymph node, Tonsil | .. | Fludarabine | yes |
| Cluster of Differentiation 86 | CD86 | \| Macrophage markers, Control of immune tolerance by vasoactive intestinal peptide, PI3K/AKT/mTOR - VitD3 Signalling, Inflammatory Response Pathway, Costimulation by the CD28 family, Interactions between immune cells and microRNAs in tumor microenvironment, IL-3 Signaling Pathway, Interleukin-10 signaling, Toll-like Receptor Signaling Pathway, Allograft Rejection, Regulation of toll-like receptor signaling pathway, PIP3 activates AKT signaling, PIP3 activates AKT signaling, Constitutive Signaling by Aberrant PI3K in Cancer, Costimulation by the CD28 family \| \| --- \| | 29 | Thyroid gland, Bone marrow, Oral mucosa, Testis, Prostate, Vagina, Cervix (uterine) | Cerebral cortex, Hippocampus, Caudate, Cerebellum, Appendix, Tonsil, Spleen, Lung, Bronchus, Liver, Gallbladder, Pancreas, Salivary gland, Esophagus, Stomach, Duodenum, Small intestine, Colon, Rectum, Urinary bladder, Seminal vesicle, Fallopian tube, Breast, Endometrium, Placenta, Skin | Lymph node | .. | Pamidronate | YES |
| Cyclin-dependent kinase 2 | CDK2 | S Phase, TP53 Regulates transpription of cell cycle genes, G1 to S cell cycle control, DNA damage/Telomere stree induced senescence, Mitotic G1-G1/S phases,Signaling by PTK6, ATM signaling Pathway, Senescence and Autophagy in Cancer, ID signaling pathway, Cell Cycle checkpoints, DNA IR-damage and cellular response via ATR, cell cycle, Human thyroid Stimulating Hormone (TSH) signaling pathway, M/G1 transition, DNA replication Pre-initiation, Small cell lung cancer, Signaling pathways in glioblastoma, Had and neck squamous cell carcinoma, Meiotic recombination, Integrated Breast cancer pathway, Factors involved in megakaryocyte development and platelet production, Regulation of DNA replication, Vitamin D Receptor Pathway, Oncostatin M signaling pathway, transcriptional regulation of granulopoiesis | 30 | Hippocampus, Caudate, Thyroid gland, Adrenal gland, Colon, Gallbladder, Kidney, Urinary bladder, Testis, Epididymis, Prostate, Vagina, Fallopian tube, Cervix, uterine, Heart muscleSkin | Oral mucosa, Esophagus, Stomach, Duodenum, Small intestine, Rectum, Placenta, Appendix, Lymph node, Tonsil, Bone marrow | .. | .. | Apremilast, Daunorubicin | .. |
| Cyclin-dependent kinase 4 | CDK4 | H19 action Rb-E2F1 signaling and CDK-Beta-catenin activity, G1 to S cell cycle control, S Phase, Tumor supressor activity of SMARCB1, Ovarian Infertility Genes, Signaling by 0PTK6, Meiotic recombination, PPAR Alpha Pathway, T-cell antigen receptor (TCR) pathway during Staphylococcus aureus infection, Oncogene Induced Senescence, Human 0thyroid stimulating hormone (TSH) signaling pathway, Mitotic G1-G1/S phases, Non-small cell lung cancer, Melanoma, Bladder Cancer, Small cell lung cancer, Senescence and 0Autophagy in cancer, Signalling Pathway in glioblastoma, Head and Neck squamous cell carcinoma, chromatin organization, Transcriptional regulation of white adipocyte 0differentiation, Pancreatic adenocarcinoma pathway, Cell cycle, miRNA Regulation of DNA damage response, transcriptional regulation by RUNX2, transcriptional regulation of whyte adipocyte differentiation, transcriptional regulation of granulopoiesis | 31 | Thyroid gland, Bronchus, Oral mucosa, Salivary gland, Esophagus, Small intestine, RectumGallbladder, Pancreas, Kidney, Urinary bladder, Testis, Epididymis, Vagina, Cervix, uterine, Breast, Smooth muscle, Soft tissue, Skin, Appendix, Spleen, Lymph node, Tonsil | Adrenal gland, Duodenum, Endometrium, Heart muscle | Ovary, Placenta | .. | Apremilast | .. |
| Cyclin-dependent kinase 6 | CDK6 | G1 to S cell cycle control, Metastatic brain tumor, Tumor suppressor activity of SMARCB1, Regulation of RUNX1 expression and activity, miR-targeted genes leucocytes-TarBase, Oncogene induced senescence, Non-small cell lung cancer, Melanoma, LncRNA involvement in canonical Wnt signaling and colorectal cancer, Small cell lung cancer, Senescence and autophagy in cancer, Signaling pathways in glioblastoma, Head and Neck squamous cell carcinoma, Wnt Signaling Pathway (Netpath), miR-targeted genes in epithilium - TarBase, Pancreatic adenocarcinoma pathway, miRNAs involved in DNA damage, Cell cycle, miRNA regulation of DNA damage response, DNA damage Response | 32 | Cerebral cortex, Cerebellum, Thyroid gland, Adrenal gland, Nasopharynx, Bronchus, Oral mucosa, Salivary gland, Esophagus, Colon, Pancreas, Kidney, Urinary bladder, Epididymis, Seminal vesicle, Prostate, Vagina, Ovary, Fallopian tube, Endometrium, Cervix, uterine, Smooth muscle, Skeletal muscle, Adipose tissue, Skin, Spleen, | Parathyroid gland, Lung, Stomach, Duodenum, Small intestine, Rectum, Gallbladder, Testis, Placenta, Breast, Lymph node, Tonsil, Bone marrow | .. | .. | Apremilast | Yes |
| Cysteinyl leukotriene receptor 1 | CYSLTR1 | G alpha (q) signalling events, Class A/1 (Rhodopsin-like receptors) | 33 | Appendix, Tonsil, Gallbladder, Pancreas, Oral mucosa, Colon, Rectum, Urinary bladder, Vagina, Uterine cervix | Bone Marrow, Spleen, Esophagus, Stomach, Skin | Lung | .. | Montelukast | YES |
| Cytochrome P450 Family 1 Subfamily B Member 1 | CYP1B1 | Sulindac Metabolic pathway, Estrogen receptor pathway, Benzo(a)pyrene metabolism, miR-targeted genes in adipocytes - TarBase, Aryl Hydrocarbon receptor pathway, Estrogen metabolism, Tamoxifen metabolism, Melatonin metabolism and effects, Oxidation by cytochrom P450, Arachidonic acid metabolism, Aryl hydrocarbon receptor netpath, Liver steatosis AOP, Tryptophan metabolism, Metapathway biotransformation Phase I and II, miR-targeted genes in ephithelium - TarBase, Nuclear receptors Meta-pathway, miR-targeted genes in muscle cell - TarBase, miR-targeted genes in lymphocytes - TarBase, Phase I - Functionalization of compounds | 34 | Nasopharynx,Bronchus, Salivary gland, Liver, Gallbladder, Vagina, Ovary, Fallopian tube, Endometrium, Cervix, uterine, Placenta, Soft tissue, Skin, Bone marrow | Cerebellum, Hippocampus, Caudate, Parathyroid gland, Adrenal gland, Lung, Esophagus, Stomach, Small intestine, Rectum, Pancreas, Urinary bladder, Testis, Epididymis, Seminal vesicle, Prostate, Breast, Heart muscle, Smooth muscle, Adipose tissue, Appendix | Cerebral cortex, Kidney, Skeletal muscle | Colon not detected | Medroxyprogesterone acetate | yes |
| Cytochrome P450 family 19 subfamily A member 1 | CYP19A1 | Steroidogenic pathway, Oxidation by Cytochrome P450, Relationship between inflammation, COX-2 and EGFR, Follicle Stimulating Hormone (FSH) signaling pathway, Metabolism of steroid hormones, Metapathway biotransformation Phase I and II, Integrated Breast Cancer Pathway, Tryptophan metabolism, Phase I - Functionalization of compounds, | 35 | .. | .. | Placenta | Adipose tissue, amygdala, artery, bone, brain, fetus, hippocampus, hypothalamus, liver, mammary gland, ovary, prostate, spermatozoa, testis, uterus, vascular system | Alendronate, Mefloquine | YES |
| Cytochrome P450 Family 2 Subfamily D Member 6 | CYP2D6 | Phase I - Functionalization of compounds, Oxidation by Cytochrome P450, Fatty Acid Omega Oxidation, Vitamin D Receptor Pathway, Aripiprazole Metabolic Pathway, Tamoxifen metabolism, Codeine and Morphine Metabolism, Melatonin metabolism and effects, Metapathway biotransformation Phase I and II, Biosynthesis of DHA-derived SPMs | 36, 37 | Soft tissue | .. | Liver, Duodenum, Small intestine | .. | Tetrabenazine | .. |
| DNA Damage Inducible Transcript 3 (a.k.a C/EBP homologous protein | DDIT3 | FOXO-mediated transcription of cell death genes, ATF4 activates genes in response to endoplasmic reticulum stress, ATF6 (ATF6-alpha) activates chaperone genes, Photodynamic therapy-induced unfolded protein response, Transcriptional cascade regulating adipogenesis, White fat cell differentiation, p38 MAPK Signaling Pathway, Adipogenesis, Preimplantation Embryo, Nonalcoholic fatty liver disease, MAPK Signaling Pathway | 38 | Thyroid gland, Parathyroid gland, Appendix, Lymph node, Tonsil, Spleen, Heart muscle, Skeletal muscle, Smooth muscle, Liver, Pancreas, Salivary gland, Esophagus, Prostate, Epididymis, Seminal vesicle, Breast, Vagina, Cervix (uterine), Endometrium, Placenta, Soft tissue | Hippocampus, Caudate, Cerebellum, Adrenal gland, Bone marrow, Lung, Nasopharynx, Bronchus, Gallbladder, Oral mucosa, Stomach, Duodenum, Small intestine, Colon, Rectum, Kidney, Urinary bladder, Testis, Fallopian tube, Ovary, Skin | Cerebral cortex | .. | Etoposide | .. |
| DNA gyrase (a.k.a DNA topoisomerase II alpha) | TOP2A | SUMOylation of DNA replication proteins, Circadian rhythm related genes, Mitotic G1-G1/S phases | 39 | Parathyroid gland, Adrenal gland, Lung, Nasopharynx, Bronchus, Gallbladder, Seminal vesicle, Fallopian tube, Breast | Appendix, Spleen, Oral mucosa, Esophagus, Stomach, Duodenum, Small intestine, Colon, Rectum, Urinary bladder, Prostate, Vagina, Uterine Cervix, Endometrium, Placenta, Skin | Bone marrow, Lymph node, Tonsil, Testis | .. | Etoposide, Daunorubicin | YES |
| DNA methyltransferase 1 activity | DNMT1 | DNA methylation, NoRC negatively regulates rRNA expression, PRC2 methylates histones and DNA, SUMOylation of DNA methylation proteins | 40-42 | Nasopharynx, Bronchus, Oral mucosa, Esophagus, Stomach, Duodenum, Small intestine, Colon, Rectum, vagina, skin | Appendix | Bone Marrow, Lymph Node, Tonsil, testis, placenta | Cerebral cortex | Ifosfamide, Azacitidine | .. |
| DNA Methyltransferase 3 Alpha | DNMT3A | SUMOylation of DNA methylation proteins, Hematopoietic Stem Cell Gene Regulation by GABP alpha/beta Complex, DNA methylation, PRC2 methylates histones and DNA , One Carbon Metabolism, Trans-sulfuration pathway, Trans-sulfuration and one carbon metabolism, One carbon metabolism and related pathways, MTHFR deficiency, Chromatin organization | 43, 44 | Bone marrow | Cerebral cortex, Hippocampus, Caudate, Spleen, Heart muscle, Prostate, Soft tissue | Cerebellum, Thyroid gland, Parathyroid gland, Adrenal gland, Appendix, Lymph node, Tonsil, Skeletal muscle, Smooth muscle, Lung, Nasopharynx, Bronchus, Liver, Gallbladder, Pancreas, Salivary gland, Oral mucosa, Esophagus, Stomach, Duodenum, Small intestine, Colon, Rectum, Kidney, Urinary bladder, Testis, Epididymis, Seminal vesicle, Fallopian tube, Breast, Vagina, Cervix (uterine), Endometrium, Ovary, Placenta, Skin | .. | Azacitidine, Daunorubicin | .. |
| DNA polymerase alpha 1, catalytic subunit | POLA1 | [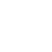](https://www.sciencedirect.com/science/journal/00029297)   \| Synthesis of DNA; Telomere Maintenance; Pyrimidine metabolism; Mitotic G1-G1/S phases; M/G1 Transition; DNA Replication Pre-Initiation; Retinoblastoma Gene in Cancer \| \| --- \| | 45 | Cerebellum, Thyroid gland, Adrenal gland, Liver, Seminal vesicle, Prostate, Ovary, Fallopian tube, Breast, Smooth muscle | Cerebral cortex, Hippocampus, Caudate, Parathyroid gland, Nasopharynx, Bronchus, Lung, Salivary gland, Gallbladder, Pancreas, Kidney, Urinary bladder, Testis, Epididymis, Endometrium, Skin, Spleen | Oral mucosa, Esophagus, Stomach, Duodenum, Small intestine, Colon, Rectum, Vagina, Uterine Cervix, Placenta, Heart muscle, Appendix, Lymph node, Tonsil, Bone marrow | .. | Clofarabine, Cytarabine, Fludarabine | YES |
| DNA polymerase | POLB | Resolution of Abasic Sites (AP sites), Nucleotide Metabolism, DNA IR-damage and cellular response via ATR, DNA IR-damage and cellular response via ATR | 46 | Hippocampus, Caudate, Appendix, Bone marrow, Lung, Duodenum, Small intestine, Vagina, Soft tissue | Cerebral cortex, Thyroid gland, Parathyroid gland, Adrenal gland, Lymph node, Spleen, Skeletal muscle, Nasopharynx, Bronchus, Liver, Pancreas, Oral mucosa, Esophagus, Stomach, Colon, Rectum, Kidney, Prostate, Epididymis, Seminal vesicle, Fallopian tube, Cervix (uterine), Endometrium, Ovary, Placenta, Skin | Cerebellum, Tonsil, Gallbladder, Salivary gland, Urinary bladder, Testis, Breast | .. | Cytarabine | YES |
| DNA polymerase | POLD1 | Telomere Maintenance, Synthesis of DNA, DNA Mismatch Repair, HDR through Homologous Recombination (HRR) or Single Strand Annealing (SSA), Resolution of Abasic Sites (AP sites), DNA Damage Bypass, Nucleotide Excision Repair, Cytosolic iron-sulfur cluster assembly, Nucleotide Metabolism, Telomere Maintenance, Resolution of Abasic Sites, Pyrimidine metabolism, DNA Replication | 47 | Liver | Cerebral cortex, Hippocampus, Caudate, Parathyroid gland, Adrenal gland, Bone marrow, Spleen, Heart muscle, Skeletal muscle, Smooth muscle, Bronchus, Gallbladder, Prostate, Epididymis, Seminal vesicle, Breast, Vagina, Endometrium, Soft tissue | Cerebellum, Thyroid gland, Appendix, Lymph node, Tonsil, Lung, Nasopharynx, Pancreas, Salivary gland, Oral mucosa, Esophagus, Stomach, Duodenum, Small intestine, Colon, Rectum, Kidney, Urinary bladder, Testis, Fallopian tube, Cervix (uterine), Ovary, Placenta, Skin | .. | Clofarabine, Cytarabine | .. |
| DNA Topoisomerase 1 | TOP1 | SUMOylation of DNA replication proteins; Ebola Virus Pathway on Host; Circadian rhythm related genes | 48 | Spleen, Heart muscle, Smooth muscle, Skeletal muscle, Adipose tissue | Parathyroid gland, Lung, Salivary gland, Liver, Pancreas, Kidney, Testis, Seminal vesicle, Prostate, Fallopian tube, Uterine Cervix, Breast, Soft tissue | Cerebral cortex, Cerebellum, Hippocampus, Caudate, Thyroid gland, Adrenal gland, Nasopharynx, Bronchus, Oral mucosa, Esophagus ,Stomach, Duodenum, Small intestine, Colon, Rectum, Gallbladder, Urinary bladder, Epididymis, Vagina, Ovary, Endometrium, Placenta, Skin, Appendix, Lymph node, Tonsil, Bone marrow | .. | Topotecan | YES |
| Dopamine receptor D2 | DRD2 | G alpha (i) signalling events, Class A/1 (Rhodopsin-like receptors), Nicotine Activity on Dopaminergic Neurons, Monoamine GPCRs, Phosphodiesterases in neuronal function, Common Pathways Underlying Drug Addiction | 49-51 | .. | .. | Cerebral cortex, hippocampus, caudate | .. | Metoclopramide | YES |
| E2F Transcription Factor 1 | E2F1 | H19 action Rb-E2F1 signaling and CDK-Beta-catenin activity, Mammary gland development pathway - Involution (Stage 4 of 4), Transcriptional Regulation by E2F6,Retinoblastoma Gene in Cancer, Mitotic G1-G1/S phases, Intrinsic Pathway for Apoptosis, G1 to S cell cycle control, TP53 Regulates Transcription of Cell Cycle Genes, The effect of progerin on the involved genes in Hutchinson-Gilford Progeria Syndrome, DNA Damage Response, miRNAs involved in DNA damage response, Oncogene Induced Senescence, miRNA Regulation of DNA Damage Response, Prader-Willi and Angelman Syndrome, Adipogenesis, Signaling Pathways in Glioblastoma, Senescence and Autophagy in Cancer, Oxidative Stress Induced Senescence, DNA IR-Double Strand Breaks (DSBs) and cellular response via ATM, Non-small cell lung cancer, Bladder Cancer, Integrated Breast Cancer Pathway, DNA IR-damage and cellular response via ATR, Aryl Hydrocarbon Receptor, Human Thyroid Stimulating Hormone (TSH) signaling pathway, Cell Cycle, MECP2 and Associated Rett Syndrome, Pancreatic adenocarcinoma pathway, miR-targeted genes in muscle cell - TarBase, miR-targeted genes in lymphocytes - TarBase, S Phase, Breast cancer pathway, EGF/EGFR Signaling Pathway, miR-targeted genes in epithelium - TarBase, Regulation of DNA replication, Spinal Cord Injury, Pre-NOTCH Expression and Processing, M/G1 Transition, DNA Replication Pre-Initiation, Mitotic G2-G2/M phases, Transcriptional regulation of granulopoiesis | 52 | Thyroid gland, Heart muscle, Liver | Smooth muscle, Lung, Bronchus, Gallbladder, Salivary gland, Oral mucosa, Stomach, Duodenum, Small intestine, Urinary bladder, Prostate, Epididymis, Seminal vesicle, Fallopian tube, Breast, Cervix (uterine), Endometrium, Ovary, Soft tissue | Cerebral cortex, Hippocampus, Cerebellum, Parathyroid gland, Adrenal gland, Appendix, Bone marrow, Lymph node, Tonsil, Spleen, Nasopharynx, Esophagus, Colon, Rectum, Testis, Vagina, Placenta, Skin | .. | Etoposide | YES |
| Epidermal Growth Factor Receptor | EGFR | Signaling by EGFR, EGF/EGFR Signaling Pathway, Relationship between inflammation, COX-2 and EGFR, ERK Pathway in Huntington's Disease, Signaling by PTK6, Aryl Hydrocarbon Receptor Pathway, Extracellular vesicle-mediated signaling in recipient cells, Photodynamic therapy-induced AP-1 survival signaling, Signaling by ERBB2, RAC1/PAK1/p38/MMP2 Pathway, Gastrin-CREB signalling pathway via PKC and MAPK, Transcriptional regulation by the AP-2 (TFAP2) family of transcription factors, Aryl Hydrocarbon Receptor, Signaling Pathways in Glioblastoma, Pathways Regulating Hippo Signaling, Signaling by ERBB4, Hippo-Merlin Signaling Dysregulation, JAK/STAT, miR-targeted genes, Leptin signaling pathway, Androgen receptor signaling pathway, ErbB Signaling Pathway, Ras Signaling, Regulation of Actin Cytoskeleton, Regulatory circuits of the STAT3 signaling pathway, PIP3 activates AKT signaling, Focal Adhesion, AGE/RAGE pathway, ESC Pluripotency Pathways, Focal Adhesion-PI3K-Akt-mTOR-signaling pathway, MAPK Signaling Pathway, L1CAM interactions, Nuclear Receptors Meta-Pathway, PI3K-Akt Signaling Pathway, RAF/MAP kinase cascade | 53 | .. | Tonsil, Skeletal muscle, Liver, Urinary bladder, Testis, Vagina, Skin | Placenta | .. | Temozolomide, Etoposide | YES |
| Estrogen receptor | ESR1 | Estrogen Receptor Pathway, ESR-mediated signaling, Relationship between inflammation, COX-2 and EGFR, Mammary gland development pathway - Puberty (Stage 2 of 4), Leptin signaling pathway, Breast cancer pathway, Transcriptional regulation by the AP-2 (TFAP2) family of transcription factors, Signaling by ERBB4, JAK/STAT, Mammary gland development pathway - Pregnancy and lactation (Stage 3 of 4), Transcriptional regulation by RUNX1, Regulation of RUNX2 expression and activity, Nuclear receptors, Nuclear receptors meta-pathway, SUMOylation of intracellular receptors, Integrated Breast Cancer Pathway, Generic Transcription Pathway, PIP3 activates AKT signaling, Deubiquitination | 54 | Smooth muscle | Breast | Fallopian tube, Vagina, Uterine Cervix, Endometrium | .. | Levonorgestrel, Etonogestrel | YES |
| Eukaryotic Translation Initiation Factor 2 Alpha Kinase 2 | EIF2AK2 (a.k.a. PKR) | Host Interactions with Influenza Factors, Translation Factors, Type II interferon signaling (IFNG), ISG15 antiviral mechanism, Measles virus infection, Ebola Virus Pathway on Host | 55 | Cerebellum, Thyroid gland, Parathyroid gland, Adrenal gland, Oral mucosa, Salivary gland, Colon, Pancreas, Kidney, Testis, Epididymis, Seminal vesicle, Prostate, Vagina, Fallopian tube, Endometrium, Cervix, uterine, Breast, Skin, Bone marrow | Caudate, Nasopharynx, Bronchus, Lung, Stomach, Duodenum, Small intestine, Rectum, Placenta, Appendix, Lymph node, Tonsil | Cerebral cortex, Hippocampus, Urinary bladder | .. | Interferon alpha-2 | YES |
| Eukaryotic translation initiation factor 4E | EIF4E | mTOR signalling, Eukaryotic Translation Initiation, Deadenylation-dependent mRNA decay, ISG15 antiviral mechanism, Processing of Capped Intron-Containing Pre-Mrna, Translation inhibitors in chronically activated PDGFRA cells, Hypertrophy Model, Deadenylation-dependent mRNA decayHypertrophy Model, mTOR signalling, Mammary gland development pathway - Pregnancy and lactation, Translation Factors,4-hydroxytamoxifen, Dexamethasone, and Retinoic Acids Regulation of p27 Expression, Interferon type, Leptin signaling pathway, Insulin Signaling, Angiopoietin Like Protein 8 Regulatory Pathway, JAK/STAT, Focal Adhesion-PI3K-Akt-mTOR-signaling pathway, Fragile X Syndrome, PI3K-Akt Signaling Pathway, miR-targeted genes in muscle cell - TarBase, miR-targeted genes in lymphocytes - TarBase, Brain-Derived Neurotrophic Factor (BDNF) signaling pathway, VEGFA-VEGFR2 Signaling Pathway | 56, 57 | Caudate, Spleen, Heart muscle, Skeletal muscle, Smooth muscle, Liver, Soft tissue | Hippocampus, Cerebellum, Thyroid gland, Parathyroid gland, Adrenal gland, Appendix, Bone marrow, Tonsil, Lung, Nasopharynx, Bronchus, Gallbladder, Salivary gland, Oral mucosa, Esophagus, Stomach, Duodenum, Small intestine, Colon, Rectum, Kidney, Urinary bladder, Testis, Prostate, Epididymis, Seminal vesicle, Fallopian tube, Breast, Vagina, Cervix (uterine), Endometrium, Ovary, Placenta, Skin | Cerebral cortex, Lymph node, Pancreas | .. | Etoposide | .. |
| Fibroblast growth factor receptor 1 | FGFR1 | Signaling by FGFR1, PIP3 activates AKT signaling, Constitutive Signaling by Aberrant PI3K in Cancer, RAF/MAP kinase cascade, Signaling Pathways in Glioblastoma, Pathways Regulating Hippo Signaling, Neural Crest Differentiation, Hippo-Merlin Signaling Dysregulation, Angiogenesis, Thermogenesis, Mesodermal Commitment Pathway, Ras Signaling, Regulation of Actin Cytoskeleton, ESC Pluripotency Pathways, Focal Adhesion-PI3K-Akt-mTOR-signaling pathway, MAPK Signaling Pathway, PI3K-Akt Signaling Pathway, L1CAM interactions, RAF/MAP kinase cascade | 58, 59 | Parathyroid gland, Lymph node, Spleen, Skeletal muscle, Liver, Soft tissue | Cerebral cortex, Hippocampus, Caudate, Cerebellum, Thyroid gland, Adrenal gland, Appendix, Bone marrow, Tonsil, Heart muscle, Smooth muscle, Lung, Nasopharynx, Bronchus, Pancreas, Salivary gland, Oral mucosa, Stomach, Duodenum, Small intestine, Colon, Rectum, Kidney, Urinary bladder, Testis, Prostate, Epididymis, Seminal vesicle, Breast, Vagina, Cervix (uterine), Endometrium, Ovary, Skin | Gallbladder, Esophagus, Fallopian tube, Placenta | .. | Etoposide, Finasteride | YES |
| Fibroblast growth factor receptor 2 | FGFR2 | Signaling by FGFR2, BMP signaling pathway in eyelid development, Hair follicle development: organogenesis (part 2 of 3), Angiogenesis, Hair follicle development: induction (part 1 of 3), Patways regulating Hippo Signaling, Hippo-Merlin signaling Dysregulation, Neural crest differentiation, Endometrial cancer, Signaling pathways in glioblastoma, 22q11.2 deletion syndrome, Ectoderm differentiation, Regulation of actin cytoskeleton, Ras signaling, ESC pluripotency pathways, Focal adhesion-PI3K-Akt-mTOR-signaling pathway, MicroRNA in cardiomyocyte hypertrophy, MAPK signaling pathway, PI3K-Akt signaling path, RAF/MAP kinase cascade | 60 | Parathyroid gland, Stomach, Duodenum, Small intestine, Colon, Rectum, Kidney, Appendix, Spleen, Lymph node | Cerebral cortex, Cerebellum, Hippocampus, Caudate, Thyroid gland, Adrenal gland, Lung, Oral mucosa, Liver, Gallbladder, Pancreas, Urinary bladder, Seminal vesicle, Vagina, Endometrium, Placenta, Breast, Heart muscle, Smooth muscle, Skeletal muscle, Bone marrow | Nasopharynx, Bronchus, Esophagus, Testis, Epididymis, Cervix, uterine, Skin, Tonsil | Soft tissue not detected | Interferon alpha-2 | YES |
| fms related tyrosine kinase 3 [a.k.a. Cluster of differentiation antigen 135 (CD135)] | FLT3 | Hematopoietic Stem Cell Gene Regulation by GABP alpha/beta Complex, Wnt/beta-catenin Signaling Pathway in Leukemia, Pathways Regulating Hippo Signaling, Ras Signaling, PI3K-Akt Signaling Pathway, RAF/MAP kinase cascade, Other interleukin signaling | 61 | .. | .. | .. | Bone marrow, Brain, Liver, Lymph node, Placenta, Spleen, Testis | Azacitidine, Clofarabine, Cytarabine, Daunorubicin | YES |
| GATA Binding Protein 1 | GATA1 | Hematopoietic Stem Cell Differentiation, RUNX1 regulates genes involved in megakaryocyte differentiation and platelet function, Factors involved in megakaryocyte development and platelet production, Transcriptional regulation by RUNX1 | 62 | .. | .. | Bone marrow | .. | Cytarabine, Daunorubicin | .. |
| Glutamate Ionotropic Receptor AMPA Type | GRIA1 | Cargo concentration in the ER, Fragile X Syndrome, Common Pathways Underlying Drug Addiction, Phosphodiesterases in neuronal function, Glutamate binding, activation of AMPA receptors and synaptic plasticity, COPII-mediated vesicle transport, Amyotrophic lateral sclerosis (ALS), Activation of NMDA receptors and postsynaptic events, Brain-Derived Neurotrophic Factor (BDNF) signaling pathway, Activation of NMDA receptors and postsynaptic events, Glutamate binding, activation of AMPA receptors and synaptic plasticity, Synaptic adhesion-like molecules | 63 | Hippocampus, Caudate, Soft tissue | Cerebral cortex, Cerebellum | .. | .. | Perampanel | .. |
| Glutamate Ionotropic Receptor AMPA Type | GRIA2 | Glutamate binding, activation of AMPA receptors and synaptic plasticity, Fragile X Syndrome, Common Pathways Underlying Drug Addiction, Transcriptional Regulation by MECP2, Activation of NMDA receptors and postsynaptic events, miR-targeted genes in muscle cell - TarBase, Brain-Derived Neurotrophic Factor (BDNF) signaling pathway | 64, 65 | .. | Hippocampus, Caudate, Cerebellum | Cerebral cortex | .. | Perampanel | .. |
| Glutamate Ionotropic Receptor AMPA Type | GRIA3 | Glutamate binding, activation of AMPA receptors and synaptic plasticity, Common Pathways Underlying Drug Addiction, Activation of NMDA receptors and postsynaptic events, Brain-Derived Neurotrophic Factor (BDNF) signaling pathway, activation of AMPA receptors and synaptic plasticity, Synaptic adhesion-like molecules | 66 | Cerebral cortex, Hippocampus, Caudate, Cerebellum | .. | .. | .. | Perampanel | .. |
| Glutamate Ionotropic Receptor AMPA Type | GRIA4 | Glutamate binding, activation of AMPA receptors and synaptic plasticity, Common Pathways Underlying Drug Addiction, Activation of NMDA receptors and postsynaptic events, Synaptic adhesion-like molecules | 67 | .. | .. | .. | Brain | Perampanel | .. |
| Glutamate Ionotropic Receptor NMDA Type | GRIN1 | RAF/MAPK cascade, Activation of NMDA receptors and postsynaptic events, Assembly and cell surface presentation of NMDA receptors, EPH-Ephrin signaling, Synaptic adhesion-like molecules, Neurexins and neuroligins; Common Pathways Underlying Drug Addiction, Phosphodiesterases in neuronal function, NO/cGMP/PKG mediated Neuroprotection | 68 | Parathyroid gland, Salivary gland, prostate, epididymis, ovary, soft tissue | Thyroid gland, adrenal gland, bone marrow, appendix, tonsil, lymph node, spleen, lung, nasopharynx, bronchus, gallbladder, pancreas, oral mucosa, stomach, esophagus, duodenum, small intestine, colon, rectum, kidney, urinary bladder, testis, breast, vagina, endometrium, ovary, placenta, uterine cervix, skin | Cerebral cortex, hippocampus, caudate, cerebellum | .. | Memantine | .. |
| Glutamate Ionotropic Receptor NMDA Type | GRIN2A | Activation of NMDA receptors and postsynaptic events, Assembly and cell surface presentation of NMDA receptors, Neurexins and neuroligins; Synaptic adhesion-like molecules, Transcriptional Regulation by MECP2 | 68 | Fallopian tube | Cerebral cortex | Cerebellum | .. | Memantine | YES |
| Glutamate Ionotropic Receptor NMDA Type | GRIN2B | RAF/MAPK cascade, Activation of NMDA receptors and postsynaptic events, Assembly and cell surface presentation of NMDA receptors, EPH-Ephrin signaling, Synaptic adhesion-like molecules, Neurexins and neuroligins; Transcriptional Regulation by MECP2; Signaling by NTRK2 (TRKB) | 68 | .. | .. | Cerebellum | .. | Memantine | .. |
| Glutamate Ionotropic Receptor NMDA Type Subunit 2C | GRIN2C (a.k.a. GluN2C) | SALM protein interactions at the synapse, Synaptic adhesion-like molecules, Phosphodiesterases in neuronal function, Synaptic signaling pathways associated with autism spectrum disorder, Neurexins and neuroligins, NO/cGMP/PKG mediated Neuroprotection, Vitamin D-sensitive calcium signaling in depression, Alzeimers disease | 69 | .. | .. | .. | Amygdala, Caudate nucleus, Corpus callosum, Heart, Hippocampus, Hypothalamus, Pancreas, skeletal muscle, Substantia nigra, Subthalamic nucleus, thalamus | Memantine | no |
| Glutamate Ionotropic Receptor NMDA Type | GRIN2D | RAF/MAPK cascade, Activation of NMDA receptors and postsynaptic events, Assembly and cell surface presentation of NMDA receptors, EPH-Ephrin signaling, Synaptic adhesion-like molecules, Neurexins and neuroligins | 70 | .. | .. | .. | Cerebral cortex, cerebellum, cerebellar cortex, fetus, granular cell, megakaryocyte, Purkinje cell | Memantine | .. |
| Glutamate Ionotropic Receptor NMDA Type | GRIN3A | Assembly and cell surface presentation of NMDA receptors | 71 | .. | .. | .. | .. | Memantine | .. |
| Gonadotropin-releasing hormone receptor | GNRHR | Peptide GPCRs, GPCRs, G alpha (q) signalling events, Class A/1 (Rhodopsin-like receptors) | 72 | Adrenal gland | Pituitary gland | .. | .. | Triptorelin | YES |
| Growth Differentiation Factor 15 | GDF15 | Epithelial to mesenchymal transition in colorectal cancer | 73 | Appendix, Pancreas, Stomach, Duodenum, Small intestine, Colon, Rectum, Kidney | Urinary bladder, Prostate | Placenta | .. | Etoposide | YES |
| H2A Histone Family Member X | H2AFX | DNA methylation, ERCC6 (CSB) and EHMT2 (G9a) positively regulate rRNA expression, PRC2 methylates histones and DNA, Activation of anterior HOX genes in hindbrain development during early embryogenesis, NoRC negatively regulates rRNA expression, SIRT1 negatively regulates rRNA expression, Meiotic synapsis, Activated PKN1 stimulates transcription of AR (androgen receptor) regulated genes KLK2 and KLK3, Nucleosome assembly, Meiotic recombination, B-WICH complex positively regulates rRNA expression, Mitotic Prophase, RNA Polymerase I Transcription, ESR-mediated signaling, Proteasome Degradation, Senescence-Associated Secretory Phenotype (SASP), Oxidative Stress Induced Senescence, Amyloid fiber formation, DNA Damage Response, miRNAs involved in DNA damage response, TCF dependent signaling in response to WNT, Telomere Maintenance, Pre-NOTCH Expression and Processing, Transcriptional regulation by RUNX1, DNA Damage/Telomere Stress Induced Senescence, Gene Silencing by RNA, Chromatin organization, DNA Double Strand Break Response, Base-Excision Repair, AP Site Formation | 74 | .. | Tarathyroid gland, Adrenal gland, Appendix, Lymph node, Tonsil, Spleen, Heart muscle, Skeletal muscle, Smooth muscle, Nasopharynx, Bronchus, Liver, Gallbladder, Salivary gland, Oral mucosa, Esophagus, Small intestine, Colon, Rectum, Kidney, Urinary bladder, Prostate, Epididymis, Seminal vesicle, Breast, Vagina, Cervix (uterine), Endometrium, Ovary, Soft tissue, Skin | Cerebral cortex, Hippocampus, Caudate, Cerebellum, Thyroid gland, Bone marrow, Lung, Pancreas, Stomach, Duodenum, Testis, Fallopian tube, Placenta | .. | Temozolomide | .. |
| Insulin Growth Factor 2 | IGF2 | Signaling by Type 1 Insulin-like Growth Factor 1 Receptor (IGF1R), Regulation of Insulin-like Growth Factor (IGF) transport and uptake by Insulin-like Growth Factor Binding Proteins (IGFBPs), Response to elevated platelet cytosolic Ca2+,Cardiac Progenitor Differentiation, Apoptosis, Endochondral Ossification, MECP2 and Associated Rett Syndrome, JAK/STAT, PI3K-Akt Signaling Pathway | 75, 76 | .. | .. | Placenta | .. | Etoposide | YES |
| Interferon gamma | IFNG | Type II interferon signaling, RUNX1 and FOXP3 control the development of regulatory T lymphocytes, Development and heterogeneity of the ILC family, Selective expression of chemokine receptors during T-cell polarization, Interferon gamma signaling, Cytokines and Inflammatory Response, Hypertrophy Model, Inflammatory Response Pathway , Control of immune tolerance by vasoactive intestinal peptide, Cancer immunotherapy by PD-1 blockade, Gene and protein expression by JAK-STAT signaling after Interleukin-12 stimulation, Aryl Hydrocarbon Receptor Pathway, Photodynamic therapy-induced AP-1 survival signaling, TGF-beta Receptor Signaling, Structural Pathway of Interleukin 1 (IL-1), Allograft Rejection, socs1, Iinterferons-mediated signaling pathway, Proteasome Degradation, Senescence and Autophagy in Cancer, Folate Metabolism, Selenium Micronutrient Network, Nuclear Receptors Meta-Pathway | 77 | Hippocampus, Parathyroid gland, Adrenal gland, Appendix, Tonsil, Heart muscle, Skeletal muscle, Smooth muscle, Lung, Nasopharynx, Gallbladder, Oral mucosa, Stomach, Duodenum, Small intestine, Rectum, Kidney, Urinary bladder, Testis, Epididymis, Seminal vesicle, Cervix (uterine), Endometrium, Soft tissue, Skin | Bronchus, Salivary gland, Colon, Prostate, Placenta | .. | .. | Interferon alpha-2, Melphalan | YES |
| Interferon-alpha/beta receptor | IFNAR1 | Interferon alpha/beta signaling (IFNAR1,2); Interleukin-27,-35,-9, -20, -2, -4, -7 signaling;Regulation of RUNX2 expression and activity, HATs acetylate histones, Constitutive Signaling by NOTCH1 PEST, HD+PEST Domain Mutants, Regulation of FOXO transcriptional activity by acetylation, OTCH1 Intracellular Domain Regulates Transcription, Estrogen-dependent gene expression, SUMOylation of transcription cofactors, Activation of the TFAP2 (AP-2) family of transcription factors, Notch-HLH transcription pathway, Activation of anterior HOX genes in hindbrain development during early embryogenesis, RUNX1 regulates transcription of genes involved in differentiation of myeloid cells, TP53 Regulates Transcription of Genes Involved in Cytochrome C Release, Regulation of TNFR1 signaling | 78, 79 | Thyroid gland, Parathyroid gland, Adrenal gland, Tonsil, Spleen, Lung, Liver, Esophagus, Small intestine, Colon, Rectum, Kidney, Breast, Vagina, uterine cervix, Ovary | Cerebral cortex, Hippocampus, Caudate, Cerebellum, Appendix, Bone marrow, Lymph node, Nasopharynx, Bronchus, Gallbladder, Pancreas, Salivary gland, Oral mucosa, Stomach, Duodenum, Urinary bladder, Testis, Prostate, Epididymis, Seminal vesicle, Fallopian tube, Endometrium, Placenta, Skin | .. | .. | Interferon alpha-2, PEGInterferon alpha-2B; PEGInterferon beta-1A, Interferon-beta-1B | YES |
| Interleukin-1 | IL1B | Interleukin-1 processing, Interleukin-1 family signaling, Interleukin-4 and Interleukin-13 signaling, Interleukin-10 signaling | 80 | .. | .. | .. | Lung, macrophage, skin | Cytarabine | YES |
| Interleukin-10 | IL10 | Interleukin-4,10,13 signaling, Folate Metabolism, Allograft Rejection, Selenium Micronutrient Network, Sudden Infant Death Syndrome (SIDS) Susceptibility Pathways, Vitamin B12 Metabolism, Gene and protein expression by JAK-STAT signaling after Interleukin-12 stimulation, miRNAs involvement in the immune response in sepsis, PI3K/AKT/mTOR - VitD3 Signalling, Cytokines and Inflammatory Response, Control of immune tolerance by vasoactive intestinal peptide, | 81, 82 | Caudate, Thyroid gland, Parathyroid gland, Bone marrow, Spleen, Heart muscle, Lung, Liver, Esophagus, Stomach, Urinary bladder, Colon, Soft tissue | Cerebral cortex, Hippocampus, Cerebellum, Adrenal gland, Lymph node, Tonsil, Nasopharynx, Bronchus, Gallbladder, Pancreas, Duodenum, Kidney, Testis, Epididymis, Seminal vesicle, Fallopian tube, Breast, Cervix (uterine), Endometrium, Ovary | .. | .. | Rofecoxib | YES |
| Interleukin-11 | IL11 | Interleukin-6, 11 Signaling Pathways, Cytokines and Inflammatory Response, Glucocorticoid Receptor Pathway, Differentiation Pathway, Nuclear Receptors Meta-Pathway, | 83 | .. | .. | .. | Bone marrow, Dendritic cell, Macrophage | Alendronate, Azacitidine | YES |
| Interleukin-2 | IL2 | Interleukin-2,3,5 and GM-CSF signaling, Folate Metabolism, Corticotropin-releasing hormone signaling pathway, Focal Adhesion-PI3K-Akt-mTOR-signaling pathway, PI3K-Akt Signaling Pathway, Nuclear Receptors Meta-Pathway, RAF/MAP kinase cascade, T-Cell Receptor and Co-stimulatory Signaling, Aryl Hydrocarbon Receptor Pathway, Cytokines and Inflammatory Response Pathway, RUNX1 and FOXP3 control the development of regulatory T lymphocytes | 81 | .. | .. | .. | Blood, Bone marrow, Brain, Colon, Kidney, Leukocyte | Metoclopramide, Interferon beta 1B, Cefepime | YES |
| Interleukin-2 receptor | IL2RA | IL-2 Signaling Pathway, IL-7 Signaling Pathway, Thymic Stromal LymphoPoietin (TSLP) Signaling Pathway, Regulatory circuits of the STAT3 signaling pathway, Allograft Rejection, Focal Adhesion-PI3K-Akt-mTOR-signaling pathway, RUNX1 and FOXP3 control the development of regulatory T lymphocytes (Tregs), Interleukin-2 family signaling, Inflammatory Response Pathway, Interactions between immune cells and microRNAs in tumor microenvironment, Interleukin-3, Interleukin-5 and GM-CSF signaling, PI3K-Akt Signaling Pathway, RAF/MAP kinase cascade, | 84 | Appendix, Bone marrow | Lymph node, Tonsil, Spleen | .. | .. | Isotretinoin, Interferon beta 1B | YES |
| Interleukin-6 | IL6 | RAF-independent MAPK1/3 activation, Interleukin-6 family signaling, Senescence-Associated Secretory Phenotype (SASP), Interleukin-10 signaling, Interleukin-4 and Interleukin-13 signaling, | 80 | Appendix | Bone marrow, Lymph node, Tonsil | .. | .. | Ifosfamide, Interferon alpha-2 | YES |
| Interleukin-8 | IL8 (a.k.a. CXCL8) | G alpha (i) signalling events, Class A/1 (Rhodopsin-like receptors), Senescence-Associated Secretory Phenotype (SASP), Interleukin-10 signaling, Interleukin-4 and Interleukin-13 signaling, Activating transcription factor 4 (ATF4) activates genes in response to endoplasmic reticulum stress | 85 | Cerebral cortex, Adrenal gland, Appendix, Bone marrow, Spleen, Heart muscle, Skeletal muscle, Esophagus, Testis, Epididymis, Ovary | Lymph node, Tonsil, Stomach, Kidney | .. | .. | Medroxyprogesterone acetate, Pamidronate | YES |
| Isocitrate dehydrogenase 1 | IDH1 | Abnormal conversion of 2-oxoglutarate to 2-hydroxyglutarate, NADPH regeneration, Cytosine methylation, TCA Cycle and Deficiency of Pyruvate Dehydrogenase complex (PDHc), Glutathione metabolism, Peroxisomal protein import, Neutrophil degranulation, Amino Acid metabolism, miR-targeted genes - TarBase, | 86 | Lung, Nasopharynx, Bronchu, sSalivary gland, Esophagus, Colon, Fallopian tube, Cervix (uterine), Ovary | Thyroid gland, Appendix, Liver, Pancreas, Stomach, Duodenum, Small intestine, Rectum, Kidney, Breast, Endometrium, Soft tissue | Adrenal gland, Gallbladder, Urinary bladder, Testis, Prostate, Epididymis, Seminal vesicle | .. | Temozolomide, Azacitidine | .. |
| Kelch Like ECH Associated Protein 1 | KEAP1 | NRF2-ARE regulation, Photodynamic therapy-induced NFE2L2 (NRF2) survival signaling, Phytochemical activity on NRF2 transcriptional activation, mRNA, protein, and metabolite inducation pathway by cyclosporin A, Hereditary leiomyomatosis, Quercetin and Nf-kB/ AP-1 Induced Cell Apoptosis, NRF2 pathway, Nuclear Receptors Meta-Pathway, Neddylation, Deubiquitination, Class I MHC mediated antigen processing & presentation | 87, 88 | Seminal vesicle, Soft tissue | Hippocampus, Caudate, Appendix, Bone marrow, Lymph node, Tonsil, Spleen, Heart muscle, Skeletal muscle, Smooth muscle, Nasopharynx, Bronchus, Liver, Gallbladder, Pancreas, Salivary gland, Oral mucosa, Esophagus, Stomach, Duodenum, Small intestine, Colon, Rectum, Kidney, Urinary bladder, Testis, Prostate, Epididymis, Fallopian tube, Breast, Vagina, Endometrium, Ovary, Skin | Cerebral cortex, Cerebellum, Thyroid gland, Parathyroid gland, Adrenal gland, Lung, Cervix (uterine), Placenta | .. | Dimethyl fumarate | .. |
| Lysine Methyltransferase 2A | KMT2A | Transcriptional regulation by RUNX1, RUNX1 regulates genes involved in megakaryocyte differentiation and platelet function, Circadian rhythm related genes, Chromatin organization | 89 | Caudate, Parathyroid gland, Liver, Testis | Cerebral cortex, Hippocampus, Cerebellum, Thyroid gland, Adrenal gland, Appendix, Bone marrow, Lymph node, Tonsil, Spleen, Heart muscle, Skeletal muscle, Smooth muscle, Lung, Nasopharynx, Bronchus, Gallbladder, Pancreas, Salivary gland, Oral mucosa, Esophagus, Stomach, Duodenum, Small intestine, Colon, Rectum, Kidney, Urinary bladder, Prostate, Epididymis, Seminal vesicle, Fallopian tube, Breast, Vagina, Cervix (uterine), Endometrium, Ovary, Placenta, Soft tissue, Skin | .. | .. | Cytarabine, Daunorubicin | .. |
| Monoamine Oxidase A | MAOA | Serotonin Transporter Activity, Neurotransmitter clearance, Biogenic Amine Synthesis, Oxidative Stress, Dopamine metabolism, Melatonin metabolism and effects, Amino Acid metabolism, Sudden Infant Death Syndrome (SIDS) Susceptibility Pathways, Neurotransmitter release cycle, Interleukin-4 and Interleukin-13 signaling, Phase I - Functionalization of compounds | 90, 91 | Cerebral cortex, Skeletal muscle, Smooth muscle, Bronchus | Hippocampus, Caudate, Tonsil, Heart muscle, Liver, Testis, Epididymis, Breast, Vagina, Cervix (uterine), Skin | Thyroid gland, Parathyroid gland, Adrenal gland, Appendix, Lung, Nasopharynx, Gallbladder, Pancreas, Salivary gland, Esophagus, Stomach, Duodenum, Small intestine, Colon, Rectum, Kidney, Urinary bladder, Prostate, Seminal vesicle, Fallopian tube, Endometrium, Ovary, Placenta | .. | Phenelzine | .. |
| Monoamine oxidase B | MAOB | Dopamine metabolism, Tryptophan metabolism, Phase I - Functionalization of compounds | 92, 93 | Hippocampus, Spleen | Caudate, Cerebellum, Parathyroid gland, Bone marrow, Tonsil, Skeletal muscle, Lung, Pancreas, Esophagus, Vagina, Skin | Cerebral cortex, Thyroid gland, Adrenal gland, Appendix, Lymph node, Heart muscle, Smooth muscle, Nasopharynx, Bronchus, Liver, Gallbladder, Salivary gland, Oral mucosa, Stomach, Duodenum, Small intestine, Colon, Rectum, Kidney, Urinary bladder,Testis, Prostate, Epididymis, Fallopian tube, Breast, Uterine cervix, Endometrium, Ovary, Placenta, Soft tissue | .. | Phenelzine | YES |
| Myeloperoxidase | MPO | Benzene metabolism, Selenium micronutrient network, Folate metabolism, vitamin B12 metabolism, Neutrophil degranulation | 94 | .. | .. | Bone Marrow | .. | Fludarabine | yes |
| Neuregulin 1 | NRG1 | Apoptosis-related network due to altered Notch3 in ovarian cancer, Signaling by ERBB2, Cardiac Progenitor Differentiation, NRF2 pathway, ErbB Signaling Pathway, Signaling by ERBB4, Nuclear Receptors Meta-Pathway, RAF/MAP kinase cascade, PIP3 activates AKT signaling, Constitutive Signaling by Aberrant PI3K in Cancer, Activation of NMDA receptors and postsynaptic events | 95, 96 | Cerebral cortex, Hippocampus, Caudate, Cerebellum, Parathyroid gland, Bone marrow, Nasopharynx, Prostate | Lymph node, Spleen, Seminal vesicle, Skin | Appendix, Tonsil | .. | Cytarabine | YES |
| Notch1 | NOTCH1 | Signaling by NOTCH1, NOTCH1 regulation of human endothelial cell calcification, Gene regulatory network modelling somitogenesis, Pre-NOTCH Expression and Processing, Gastric Cancer Network 1, Heart Development, PTF1A related regulatory pathway, Notch Signaling, BMP Signaling Pathway in Eyelid Development, Role of Osx and miRNAs in tooth development, Notch Signaling Pathway, Notch Signaling Pathway, Canonical and Non-canonical Notch signaling, Cardiac Progenitor Differentiation, Primary Focal Segmental Glomerulosclerosis FSGS, Transcriptional regulation by RUNX3, miR-targeted genes in epithelium - TarBase, miR-targeted genes in muscle cell - TarBaseNeural Crest Differentiation, Regulation of beta-cell development, Pathways Affected in Adenoid Cystic Carcinoma, Transcriptional Regulation by MECP2, miR-targeted genes in lymphocytes - TarBase, Endoderm Differentiation, Hematopoietic Stem Cell Differentiation, Epithelial to mesenchymal transition in colorectal cancer, Differentiation Pathway, Signaling by NOTCH2, Transcriptional regulation by RUNX2, Transcriptional regulation by RUNX1 | 97 | .. | Cerebral cortex, Hippocampus, Caudate, Cerebellum, Thyroid gland, Parathyroid gland, Bone marrow, Lymph node, Tonsil, Spleen, Heart muscle, Skeletal muscle, Smooth muscle, Nasopharynx, Bronchus, Liver, Pancreas, Salivary gland, Oral mucosa, Esophagus, Duodenum, Small intestine, Kidney, Urinary bladder, Prostate, Epididymis, Seminal vesicle, Fallopian tube, Breast, Vagina, Cervix (uterine), Endometrium, Placenta, Soft tissue, Skin | Adrenal gland, Appendix, Lung, Gallbladder, Stomach, Colon, Rectum, Testis | .. | Temozolomide | YES |
| Nuclear Receptor Subfamily 4 Group A Member 1 | NR4A1 | Corticotropin-releasing hormone signaling pathway, Nuclear receptors, Spinal cord injury, MAPK signaling pathway, Generic transcription pathway, PIP3 activates AKT signaling | 98 | Cerebellum, Caudate, Lung, Duodenum, Small intestine, Gallbladder, Pancreas, Vagina, Ovary, Endometrium, Breast, Smooth muscle, Appendix, Lymph node | Cerebral cortex, Hippocampus, Thyroid gland, Nasopharynx, Oral mucosa, Salivary gland, Esophagus, Stomach, Colon, Rectum, Kidney, Urinary bladder, Testis, Epididymis, Seminal vesicle, Fallopian tube, Cervix, uterine, Placenta, Heart muscle, Skeletal muscle, Skin, Tonsil | Adrenal gland, Bronchus | Soft tissue not detected | Etoposide | yes |
| p110a (catalytic subunit of phosphatidylinositol 3-kinase (PI3K)) | PIK3CA | Neural Crest Cell Migration during Development; PDGFR-beta pathway; PI3K-AKT-mTOR signaling pathway and therapeutic opportunities; Nephrin family interactions; Non-small cell lung cancer;Synaptic signaling pathways associated with autism spectrum disorder; Neural Crest Cell Migration in Cancer; G13 Signaling Pathway; Microglia Pathogen Phagocytosis Pathway;Signaling by NTRK3 (TRKC); Signaling by NTRK2 (TRKB); PI3K Cascade; Overview of interferons-mediated signaling pathway; Copper homeostasis; Signaling by Type 1 Insulin-like Growth Factor 1 Receptor (IGF1R); Signaling by Erythropoietin; Signaling by SCF-KIT; MET in type 1 papillary renal cell carcinoma; Thymic Stromal LymphoPoietin (TSLP) Signaling Pathway; Pathways Affected in Adenoid Cystic Carcinoma;Small cell lung cancer; Signaling Pathways in Glioblastoma; Non-genomic actions of 1,25 dihydroxyvitamin D3; Phosphoinositides metabolism; Signaling by Insulin receptor; Interleukin-3, Interleukin-5 and GM-CSF signaling; Signaling by ERBB2; PIP3 activates AKT signaling; Signaling by ERBB4; IL-4 Signaling Pathway; Nonalcoholic fatty liver disease; Ebola Virus Pathway on Host; Costimulation by the CD28 family; DNA Damage Response (only ATM dependent); Prolactin Signaling Pathway; Human Thyroid Stimulating Hormone (TSH) signaling pathway; Insulin Signaling; ErbB Signaling Pathway; Regulation of Actin Cytoskeleton; Ras Signaling; PI Metabolism; RET signaling; Interleukin-2 family signaling; Signaling by PDGF; GPVI-mediated activation cascade; JAK/STAT; Signaling by VEGF; Toll-like Receptor Signaling Pathway; Focal Adhesion; Angiopoietin Like Protein 8 Regulatory Pathway; Breast cancer pathway; Signaling by NTRK1 (TRKA); Signaling by MET; Signaling by FGFR1; Signaling by FGFR4; Gastrin Signaling Pathway; Regulation of toll-like receptor signaling pathway; Focal Adhesion-PI3K-Akt-mTOR-signaling pathway; DAP12 interactions; Signaling by FGFR2; Signaling by EGFR; Signaling by FGFR3; Brain-Derived Neurotrophic Factor (BDNF) signaling pathway; Chemokine signaling pathway; PI3K-Akt Signaling Pathway; G alpha (q) signalling events; Cell surface interactions at the vascular wall; TCR signaling; VEGFA-VEGFR2 Signaling Pathway; Fc epsilon receptor (FCERI) signaling; Fcgamma receptor (FCGR) dependent phagocytosis | 99 | Caudate, Vagina, Ovary, Heart muscle, Smooth muscle, Skeletal muscle, Soft tissue, Adipose tissue, Bone marrow | Cerebral cortex, Hippocampus, Lung, Oral mucosa, Esophagus, Stomach, Liver, Pancreas, Urinary bladder, Epididymis, Seminal vesicle, Prostate, Uterine Cervix, Skin, Lymph node, Tonsil | Cerebellum, Thyroid gland Parathyroid gland ,Adrenal gland, Nasopharynx, Bronchus, Salivary gland, Duodenum, Small intestine, Colon, Rectum, Gallbladder, Kidney, Testis, Fallopian tube, Endometrium, Placenta, Breast, Appendix, Spleen | .. | Topotecan | YES |
| Phosphatidylinositol-4,5-Bisphosphate 3-Kinase Catalytic Subunit Gamma | PIK3CG | Leptin insulin overlap, Neural crest cell migration during development, PI3K-AKT-mTOR signaling pathway and therapeutic opportunities, G-protein beta: gamma signaling, Neural crest cell migration in cancer, Microglia pathogen phagocytosis pathway, Translation inhibitors in chronically activated PDGFRA cells, Signaling by erythropoietin, IL-5 signaling pathway, Signaling pathways in glioblastoma, Non-genomic actions of 1,25 dihydroxyvitamin D3, Phosphoinositides metabolism, Head and Neck squamous cell carcinoma, GPVI-mediated activation cascade, DNA damage response (only ATM dependent), Prolactin signaling pathway, Insulin signaling, Regulation of actin cytoskeleton, PI metabolism, JAK/STAT, Toll-like receptor signaling path, B cell receptor signaling pathway, Angiopoietin like protein 8 regulatory pathway, Regulation of toll-like receptor signaling pathway | [100](http://www.ncbi.nlm.nih.gov/pubmed/20025958) | Cerebral cortex, Caudate, Thyroid gland, Colon, Liver, Epididymis, Prostate, Vagina, Cervix, uterine | Adrenal gland, Stomach, Duodenum, Small intestine, Rectum, Gallbladder, Kidney, Urinary bladder, Testis, Seminal vesicle, Ovary, Fallopian tubeEndometriumPlacentaBreastSkinAppendixSpleenLymph nodeTonsilBone marrow | .. | .. | Fludarabine | YES |
| Parathyroid hormone | PTH | Osteoblast signaling, Endochondral ossification, Class B/2 (secretin family receptors), Vitamin D receptor pathway, G alpha (s) signaling events | 101 | .. | .. | Parathyroid gland | .. | Pamidronate | YES |
| Phosphatase And Tensin Homolog (PTEN) | PTEN | TCR signaling, PI Metabolism, TP53 Regulates Metabolic Genes, PTEN Regulation, Inositol phosphate (IP3) metabolism, Transcriptional Regulation by MECP2, Deubiquitination | 102 | Hippocampus, Caudate, Appendix, Bone marrow, Lymph node, Tonsil, Lung, Nasopharynx, Gallbladder, Pancreas, Salivary gland, Oral mucosa, Esophagus, Duodenum, Small intestine, Colon, Rectum, Kidney, Epididymis, Seminal vesicle, Fallopian tube, Breast, Uterine cervix, Placenta | Cerebral cortex, Cerebellum, Thyroid gland, Adrenal gland, Spleen, Bronchus, Stomach, Urinary bladder, Testis, Vagina, Endometrium, Soft tissue, Skin | .. | .. | Temozolomide, Topotecan | YES |
| Phosphodiesterase 4 | PDE4A | G alpha (s) signalling events, Opioid Signalling | 103, 104 | .. | .. | .. | Monocyte, Placenta, T cell | Apremilast | .. |
| Phosphodiesterase 4 | PDE4B | G alpha (s) signalling events, Opioid Signalling | 105 | Hippocampus, Thyroid gland, Appendix, Lymph node, Tonsil, Oral mucosa, Stomach, Fallopian tube, Uterine Cervix | Cerebral cortex, Caudate, Adrenal gland, Nasopharynx, Bronchus, Liver, Pancreas, Salivary gland, Duodenum, Small intestine, Colon, Rectum, Kidney, Testis, Prostate, Seminal vesicle, Endometrium, Skin | Lung, Gallbladder, Urinary bladder, Epididymis, Breast, | .. | Apremilast | .. |
| Phosphodiesterase 4 | PDE4D | G alpha (s) signalling events, Opioid Signalling | 106 | Parathyroid gland, Vagina | Cerebral cortex, Hippocampus, Caudate, Thyroid gland, Adrenal gland, Bone marrow, Lymph node, Spleen, Skeletal muscle, Smooth muscle, Lung, Nasopharynx, Bronchus, Gallbladder, Salivary gland, Oral mucosa, Esophagus, Stomach, Urinary bladder, Testis, Breast, Uterine cervix, Ovary, Placenta, Soft tissue, Skin | Cerebellum, Appendix, Tonsil, Heart muscle, Liver, Pancreas, Duodenum, Small intestine, Colon, Rectum, Kidney, Prostate, Epididymis, Seminal vesicle, Fallopian tube, Endometrium | .. | Apremilast | .. |
| Plasminogen Activator | PLAT (a.k.a. tPA) | Dissolution of Fibrin Clot, NOTCH1 regulation of human endothelial cell calcification, Senescence and Autophagy in Cancer, Complement and Coagulation Cascades, Signaling by PDGF | 107, 108 | Bronchus, Liver, Kidney, Endometrium, Skin, Cerebral cortex | Bone marrow, Lymph node, Tonsil, Spleen, Pancreas, Salivary gland, Placenta, Soft tissue | .. | .. | Melphalan | YES |
| Potassium Calcium-Activated Channel Subfamily N Member 2 | KCNN2 | potassium channels, Brain-derived Neurotrophic Factor (BDNF) signaling pathway | 109 | .. | .. | .. | Amygdala, Brain, Caudate nucleus, Cerebellum, Colon, Corpus callosum, Liver, Heart, Hippocampus, Kidney, Pituitary gland,skeletal muscle, spinal cord, Substantia nigra, thalamus | Bendroflumethiazide | no |
| Progesterone receptor | PGR | Generic Transcription Pathway, ESR-mediated signaling, SUMOylation of intracellular receptors, Signaling by ERBB4, Nuclear Receptors, Mammary gland development pathway - Pregnancy and lactation, HSP90 chaperone cycle for steroid hormone receptors (SHR), TFAP2 (AP-2) family regulates transcription of growth factors and their receptors, SUMOylation of DNA damage response and repair proteins, Recognition and association of DNA glycosylase, Displacement of DNA glycosylase by APEX1, TET1,2,3 and TDG demethylate DNA, HATs acetylate histones, SUMOylation of transcription cofactors, Estrogen-dependent gene expression | 110 | .. | Breast | Smooth muscle, Fallopian tube, Cervix (uterine), Endometrium | .. | Levonorgestrel, Etonogestrel, Megestrol, Medroxyprogesterone acetate | .. |
| Prolactin | PRL | Prolactin receptor signaling, Prolactin Signaling Pathway, Mammary gland development pathway - Pregnancy and lactation, JAK/STAT, Prostaglandin Synthesis and Regulation, Growth hormone receptor signaling, Focal Adhesion-PI3K-Akt-mTOR-signaling pathway, PI3K-Akt Signaling Pathway, Amyloid fiber formation | 111 | .. | .. | Pituitary gland | .. | Temozolomide | .. |
| Prostaglandin-Endoperoxide Synthase 2 (a.k.a. cyclooxygenase 2, COX-2) | PTGS2 (a.k.a. COX2) | Nicotinate metabolism, Arachidonic acid metabolism, Biosynthesis of DPA-derived SPMs, Biosynthesis of electrophilic ω-3 PUFA oxo-derivatives, Biosynthesis of EPA- and DHA-derived SPMs, Interleukin-4,10,13 signaling, Eicosanoid Synthesis, Prostaglandin Synthesis and Regulation, Glucocorticoid Receptor Pathway, VEGFA-VEGFR2 Signaling Pathway, Nuclear Receptors Meta-Pathway, | 112 | Bone marrow, Smooth muscle, Lung, Prostate, Cervix (uterine), Placenta, Skin | Seminal vesicle, Fallopian tube, Endometrium | Gallbladder, Urinary bladder | .. | Rofecoxib, Valdecoxib | YES |
| Ribonucleotide Reductase Catalytic Subunit M2b | RRM2B | Genotoxicity pathway; Nucleotide Metabolism; TP53 Regulates Metabolic Genes; Biomarkers for pyrimidine metabolism disorders; Pyrimidine metabolism; miRNA Regulation of DNA Damage Response; DNA Damage Response; Purine metabolism; Interconversion of nucleotide di- and triphosphates | 113 | Salivary gland, Esophagus, Liver, Ovary, Skeletal muscle, Soft tissue, Adipose tissue | Hippocampus, Oral mucosa, Stomach, Duodenum, Small intestine, Gallbladder, Pancreas, Kidney, Urinary bladder, Testis, Seminal vesicle, Uterine Cervix, Placenta, Breast, Skin, Spleen | Cerebral cortex, Cerebellum, Caudate, Thyroid gland, Parathyroid gland, Adrenal gland, Nasopharynx, Bronchus, Lung, Colon, Rectum, Epididymis, Prostate, Fallopian tube, Endometrium, Appendix, Lymph node, Tonsil, Bone marrow | .. | Clofarabine | YES |
| Retinoic acid receptor alpha | RARA | Activation of anterior HOX genes in hindbrain development during early embryogenesis, Generic Transcription Pathway, SUMOylation of intracellular receptors, Wnt/beta-catenin Signaling Pathway in Leukemia, Nuclear Receptors in Lipid Metabolism and Toxicity, Signaling by Retinoic Acid, Adipogenesis, Vitamin A and Carotenoid Metabolism | 114 | .. | .. | .. | Hippocampus | Isotretinoin | YES |
| serotonin receptors | HTR3B | Neurotransmitter receptors and postsynaptic signal transmission | 115 | Cerebellum, Appendix, Spleen | Lymph node, Tonsil | .. | .. | Metoclopramide | .. |
| Sodium-dependent serotonin transporter | SLC18A1 | Transport of bile salts and organic acids, Synaptic Vesicle Pathway | 16 | Appendix, Stomach, Duodenum ,Small intestine, Colon, Rectum | .. | Adrenal gland | .. | Tetrabenazine | .. |
| Sodium-dependent serotonin transporter (a.k.a. Norepinephrine Transporter) | SLC6A2 (a.k.a NET) | Monoamine Transport, NRF2 pathway, Nuclear Receptors Meta-Pathway, Transport of bile salts and organic acids | 116 | Ovary, Placenta | Testis, Soft tissue | Cerebral cortex | .. | Phenelzine | .. |
| Sodium-dependent serotonin transporter (a.k.a. vesicular monoamine transporter 2) | SLC18A2 (a.k.a.VMAT2) | Dopaminergic Neurogenesis, Nicotine Activity on Dopaminergic Neurons, Neurotransmitter release cycle, Transport of bile salts and organic acids, Synaptic Vesicle Pathway | 117 | Lung, Liver | Tonsil, Testis | Caudate, Adrenal gland | .. | Tetrabenazine | .. |
| Solute Carrier Family 2 Member 4 | SLC2A4 (a.k.a. GLUT4) | Translocation of SLC2A4 (GLUT4) to plasma membrane, Cellular hexose transport, Cori cycle, Glycolysis and gluconeogenesis - for workshop, Gluconeogenesis, Transcriptional regulation of white adipocyte differentiation, Glycolysis and gluconeogenesis, Adipogenesis, 22q11,2 delection syndrome, Vitamin D receptor pathway, Insulin signaling, NRF2 pathway, Focal adhesion-PI3K-Akt-mTOR-signaling pathway, Nuclear receptor meta-pathway | 118 | Adipose tissue | Smoth muscle, Skeletal muscle | Heart muscle | .. | Etoposide | yes |
| Sphingosine-1-phosphate receptor | S1PR1 | G alpha (i) signalling events, Class A/1 (Rhodopsin-like receptors),Interleukin-4 and Interleukin-13 signaling, Signal Transduction of S1P Receptor | 119 | Heart muscle, Smooth muscle, Lung, Pancreas, Seminal vesicle, Vagina, Uterine cervix, uterine, Soft tissue | Thyroid gland, Bone marrow, Lymph node, Tonsil, Spleen, Skeletal muscle, Liver, Gallbladder, Salivary gland, Oral mucosa, Esophagus, Duodenum, Small intestine, Colon, Rectum, Urinary bladder, Testis, Epididymis, Fallopian tube, Breast, Endometrium, Ovary | Cerebral cortex, Cerebellum, Parathyroid gland, Adrenal gland, Appendix, Nasopharynx, Bronchus, Stomach, Kidney, Placenta, Skin | .. | Fingolimod | YES |
| Steroid 5-alpha reductase 1 | SRD5A1 | Metabolism of steroid hormones | 120 | Tonsil, Salivary gland, Esophagus, Breast, Uterine Cervix, Endometrium, Skin | Hippocampus, Cerebellum, Thyroid gland, Parathyroid gland, Lung, Nasopharynx, Liver, Gallbladder, Pancreas, Stomach, Duodenum, Small intestine, Colon, Rectum, Testis, Seminal vesicle | Adrenal gland, Appendix, Kidney, Urinary bladder, Prostate, Epididymis, Fallopian tube | .. | Finasteride, Levonorgestrel | .. |
| Steroid 5-alpha reductase 2 | SRD5A2 | Metabolism of steroid hormones | 121 | .. | .. | .. | Prostate, Scalp, Skin | Finasteride | .. |
| Steroid Sulfatase | STS | Sphingolipid metabolism, Gamma carbozylation, hypusine formation and arylsulfatase activation, vitamin D receptor pathway | 122 | Cerebral cortex, Cerebellum, Hippocampus, Caudate, Thyroid gland, Parathyroid gland, Adrenal gland, Nasopharynx, Bronchus, Lung, Salivary gland, Stomach, Duodenum, Small intestine, Colon, Rectum, Liver, Gallbladder, Pancreas, Kidney, Urinary bladder, Testis, Epididymis, Seminal vesicle, Prostate, Ovary, Fallopian tube, Endometrium, Breast, Appendix, Tonsil, Bone marrow | .. | Placenta | .. | Triptorelin pamoate | yes |
| Survivin (a.k.a. baculoviral inhibitor of apoptosis repeat-containing 5) | BIRC5 | Cell Cycle Checkpoints, Mitotic Prometaphase, Mitotic Metaphase and Anaphase, RHO GTPases Activate Formins, RHO GTPases Activate Formins, SUMOylation of DNA replication proteins, Neddylation, TP53 Regulates Transcription of Cell Death Genes, Apoptosis, Interleukin-11 Signaling Pathway, Interleukin-4 and Interleukin-13 signaling, IL-4 Signaling Pathway | 123 | Appendix, Stomach, Duodenum, Small intestine, Colon, Rectum, Urinary bladder, Breast, Uterine cervix, Endometrium | Bone marrow, Lymph node, Tonsil, Oral mucosa, Esophagus, Vagina, Placenta, Skin | Testis | .. | Rofecoxib, Valdecoxib, Cytarabine | YES |
| TATA-Box Binding Protein | TBP | Prion disease pathway, RNA polimerase I transcription, Eukaryotic Transcription initiation, B-WICH comples positively regulates rRna expression, RNA polymerase III transcription, RNA polymerase II transcribes snRNA genes, SIRT1 negatively regulates rRNA expression, RNA polymerase II transcription, NoRC negatively regulates rRNA expression, ESR-mediated signaling, Regulation of TP53 activity throught phosphorylation, HIV life cycle | 124 | Thyroid gland, Parathyroid gland, Adrenal gland, Bronchus, Oral mucosa, Esophagus, Stomach, Duodenum, Colon, Rectum, Liver, Gallbladder, Pancreas, Kidney, Testis, Epididymis, Seminal vesicle, Prostate, Vagina, Endometrium, Cervix, uterine, Smooth muscle, Skeletal muscle, Soft tissue, Skin, Appendix, Spleen | Cerebral cortex, Cerebellum, Hippocampus, Caudate, Nasopharynx, Lung, Salivary gland, Small intestine, Urinary bladder, Fallopian tube, Placenta, Breast, Heart muscle, Adipose tissue, Bone marrow | .. | .. | Etoposide | yes |
| Tet Methylcytosine Dioxygenase 2 | TET2 | Cytosine methylation, TET1,2,3 and TDG demethylate DNA, MECP2 and Associated Rett Syndrome | 125 | .. | Cerebral cortex, Parathyroid gland, Appendix, Spleen, Smooth muscle, Lung, Liver, Pancreas, Prostate, Epididymis, Seminal vesicle, Endometrium, Ovary | Hippocampus, Caudate, Cerebellum, Thyroid gland, Adrenal gland, Bone marrow, Lymph node, Tonsil, Heart muscle, Skeletal muscle, Nasopharynx, Bronchus, Gallbladder, Salivary gland, Oral mucosa, Esophagus, Stomach, Duodenum, Small intestine, Colon, Rectum, Kidney, Urinary bladder, Testis, Fallopian tube, Breast, Vagina, Cervix (uterine), Placenta, Soft tissue, Skin | .. | Azacitidine | .. |
| Thymidylate synthase | TYMS | Serine Metabolism; Trans-sulfuration and one carbon metabolism; One Carbon Metabolism; Fluoropyrimidine Activity; Ethanol effects on histone modifications; Pyrimidine metabolism; Interconversion of nucleotide di- and triphosphates; Circadian rhythm related genes; Retinoblastoma Gene in Cancer; Mitotic G1-G1/S phases | 126 | Thyroid gland, Bronchus, Esophagus, Liver, Kidney, Prostate, Ovary, Fallopian tube, Endometrium, Uterine Cervix, Breast, Heart muscle, Smooth muscle, Skeletal muscle, Spleen, Soft tissue | Cerebral cortex, Hippocampus, Caudate, Parathyroid gland, Adrenal gland, Nasopharynx, Lung, Oral mucosa, Stomach, Duodenum, Small intestine, Colon, Rectum, Gallbladder, Pancreas, Urinary bladder, Testis, Epididymis, Seminal vesicle, Placenta, Skin, Appendix | Lymph node, Tonsil, Bone marrow | .. | Daunorubicin, Topotecan | YES |
| Transforming Growth Factor beta 1 | TGFB1 | Signaling by TGF-beta Receptor Complex, Extracellular vesicle-mediated signaling in recipient cells, Mammary gland development pathway - Embryonic development (Stage 1 of 4), Host Interactions with Influenza Factors, TGF-beta Receptor Signaling, Elastic fibre formation, Cytokines and Inflammatory Response, Simplified Interaction Map Between LOXL4 and Oxidative Stress Pathway, ACE Inhibitor Pathway, Protein alkylation leading to liver fibrosis, Signal transduction through IL1R, Dopaminergic Neurogenesis, Aryl Hydrocarbon Receptor Pathway, Selective expression of chemokine receptors during T-cell polarization, Type 2 papillary renal cell carcinoma, Platelet-mediated interactions with vascular and circulating cells, TGF-beta Signaling Pathway, Envelope proteins and their potential roles in EDMD physiopathology, Cardiac Progenitor Differentiation, Interactions between immune cells and microRNAs in tumor microenvironment, Adipogenesis, IL-3 Signaling Pathway, Senescence and Autophagy in Cancer, Hepatitis C and Hepatocellular Carcinoma, Pathways in clear cell renal cell carcinoma, Endochondral Ossification, Interleukin-11 Signaling Pathway, Differentiation Pathway, Syndecan interactions, Vitamin D Receptor Pathway, Endoderm Differentiation, NRF2 pathway, Transcriptional regulation of white adipocyte differentiation, Nonalcoholic fatty liver disease, DNA Damage Response (only ATM dependent), Primary Focal Segmental Glomerulosclerosis FSGS, Hematopoietic Stem Cell Differentiation, Cell Cycle, T-Cell antigen Receptor (TCR) Signaling Pathway, Pancreatic adenocarcinoma pathway, Epithelial to mesenchymal transition in colorectal cancer, Chromosomal and microsatellite instability in colorectal cancer, Transcriptional regulation by RUNX3, Lung fibrosis, Allograft Rejection, Allograft Rejection, Viral Acute Myocarditis, Spinal Cord Injury, Nuclear Receptors Meta-Pathway, Interleukin-4 and Interleukin-13 signaling, Deubiquitination, Extracellular matrix organization, Cell surface interactions at the vascular wall, TGFBR1 LBD Mutants in Cancer, TGFBR2 MSI Frameshift Mutants in Cancer, TGFBR2 Kinase Domain Mutants in Cancer, TGFBR1 KD Mutants in Cancer, SMAD2/3 Phosphorylation Motif Mutants in Cancer, Elastic fibre formation, Response to elevated platelet cytosolic Ca2+ | 127, 128 | Bronchus, Endometrium, Placenta | Spleen, Stomach, Small intestine | Bone marrow | .. | Etoposide, Isotretinoin, Interferon alpha-2 | YES |
| Transglutaminase 2 | TGM2 | NRF2 pathway, Glycolysis Pathway D (2), Serotonin Receptor 2 and STAT3 Signaling, NRF2-ARE regulation, Serotonin Receptor 2 and ELK-SRF/GATA4 signaling, Interactome of polycomb repressive complex 2 (PRC2), Cell-type Dependent Selectivity of CCK2R Signaling, Asparagine N-linked glycosylation, receptors targeted by epinephrine and norepinephrine (fear signal), rRNA modification in the mitochondrion, Syndecan interactions, Lysosomal oligosaccharide catabolism, Glycosaminoglycan metabolism, Interconversion of nucleotide di- and triphosphates, Cargo concentration in the ER, DNA Mismatch Repair, Metabolism of Dichloroethylene by CYP450, Epilepsy Adverse Outcome Pathway, | 129, 130 | Appendix, Stomach, Urinary bladder, Testis, Cervix (uterine), Soft tissue, Skin, Colon, Cerebral cortex | Heart muscle, Smooth muscle, Lung, Gallbladder, Endometrium | Placenta | .. | Isotretinoin, Finasteride | YES |
| Tumor Necrosis Factor (TNF) | TNF | TNF signaling, CD209 (DC-SIGN) signaling, Interleukin-10 signaling, Interleukin-4 and Interleukin-13 signaling, Cytokines and Inflammatory Response, TNF related weak inducer of apoptosis (TWEAK) Signaling Pathway, TGF-beta Receptor Signaling | 80, 131 | .. | .. | .. | Immune system, Hypothalamo-pituitary-adrenocortical (HPA) axis | Lactulose | YES |
| Tumor protein p53 | TP53 | TP53 Network, TP53 Regulates Metabolic Genes, Regulation of TP53 Expression and Degradation, Regulation of TP53 Activity through Phosphorylation, Regulation of TP53 Activity through Association with Co-factors, Regulation of TP53 Activity through Methylation, TP53 Regulates Transcription of Cell Cycle Genes, TP53 Regulates Transcription of Cell Death Genes, Regulation of TP53 Activity through Acetylation, TP53 Regulates Transcription of DNA Repair Genes, Ultraconserved region 339 modulation of tumor suppressor microRNAs in cancer, MicroRNA network associated with chronic lymphocytic leukemia, Metastatic brain tumor, Photodynamic therapy-induced AP-1 survival signaling, Oncogene Induced Senescence, Photodynamic therapy-induced HIF-1 survival signaling, LncRNA-mediated mechanisms of therapeutic resistance, G1 to S cell cycle control, The effect of progerin on the involved genes in Hutchinson-Gilford Progeria Syndrome, Sandbox PathwayOxidative Stress Induced Senescence, RAC1/PAK1/p38/MMP2 Pathway, Fluoropyrimidine Activity, Copper homeostasis, Transcriptional regulation by RUNX3, SUMOylation of transcription factors, Apoptosis Modulation and Signaling, Signaling Pathways in Glioblastoma, Wnt Signaling Pathway and Pluripotency, Senescence and Autophagy in Cancer, ATM Signaling Pathway, Amyotrophic lateral sclerosis (ALS), DNA IR-Double Strand Breaks (DSBs) and cellular response via ATM, Ferroptosis, Retinoblastoma Gene in Cancer, Factors involved in megakaryocyte development and platelet production, DNA Damage/Telomere Stress Induced Senescence, DNA IR-damage and cellular response via ATR, Oncostatin M Signaling Pathway, DNA Damage Response, Interleukin-4 and Interleukin-13 signaling, DNA Damage Response (only ATM dependent), miRNAs involved in DNA damage response, Pre-NOTCH Expression and Processing, miRNA Regulation of DNA Damage Response, Cell Cycle Checkpoints, PTEN Regulation, TGF-beta Signaling Pathway, Alzheimers Disease, MAPK Signaling Pathway, Deubiquitination, PI3K-Akt Signaling Pathway, PIP3 activates AKT signaling, PI Metabolism, Protein folding, DNA Double Strand Break Response, Generic Transcription Pathway, Mitotic G2-G2/M phases, | 132, 133 | Tonsil, Urinary bladder, Skin | Oral mucosa, Esophagus | .. | .. | Etoposide, Interferon alpha-2, Cytarabine, Daunorubicin, Ifosfamide, Temozolomide, Topotecan | YES |
| Tumour protein p73 | TP73 | TP 53 network, Regulation of TP53 activity through association with co-factors, TP53 regulates transcription of cell death genes, ATM signaling pathway, Apoptosis, Transcriptional regulation by RUNX1, Intrinsic pathway for apoptosis, DNA damagge response (only ATM dependent), Measles virus infection, Sudden infant death syndrome (SIDS) susceptibility pathways | 134 | Tonsil | Oral mucosa, Esophagus, Vagina, Fallopian tube, Cervix, uterine | Nasopharynx, Bronchus, Skin | .. | Cytarabine | YES |
| Vitamin D receptor | VDR | Non-genomic actions of 1,25 dihydroxyvitamin D3, Vitamin D in inflammatory diseases, PI3K/AKT/mTOR - VitD3 Signalling, Vitamin D Metabolism and receptor pathway, Vitamins A and D - action mechanisms, Nuclear Receptors in Lipid Metabolism and Toxicity, Nuclear Receptors Meta-Pathway, SUMOylation of intracellular receptors, Generic Transcription Pathway, | 135, 136 | .. | .. | .. | Lymphocyte, Ubiquitous | Alendronate, Bicalutamide, Cytarabine | YES |
| Voltage-Gated Calcium Channels | CACNA1B | Presynaptic depolarization and calcium channel opening, Synaptic Vesicle Pathway, MAPK Signaling Pathway | 137 | Hippocampus, Caudate, Bone marrow, Lymph node, Heart muscle, Nasopharynx, Salivary gland, Esophagus, Rectum, Urinary bladder Seminal vesicle, Vagina, Ovary, Soft tissue, Skin | Cerebral cortex, Parathyroid gland, Appendix, Tonsil, Bronchus, Liver, Gallbladder, Pancreas, Stomach, Duodenum, Small intestine, Colon, Testis, Fallopian tube, Endometrium | Adrenal gland, Kidney | .. | Ziconotide | .. |
| Voltage-Gated Potassium Channel Subfamily E Regulatory | KCNE1 | Genes targeted by miRNAs in adipocytes, Cardiac conduction | 138 | .. | .. | .. | Ear, Heart, Intestine, Kidney, Lung, Nervous system, Pancreas, Salivary gland | Indapamide | .. |
| Voltage-Gated Potassium Channel Subfamily Q Member 1 | KCNQ1 | Genes targeted by miRNAs in adipocytes, Cardiac conduction, Sudden Infant Death Syndrome (SIDS) Susceptibility Pathways, miR-targeted genes in muscle cell - TarBase, miR-targeted genes in lymphocytesl - TarBase | 139 | Cerebral cortex, Hippocampus, Nasopharynx, Salivary gland, Esophagus, Urinary bladder, Testis, Ovary | Cerebellum, Appendix, Bone marrow, Tonsil, Heart muscle, Skeletal muscle, Smooth muscle, Lung, Bronchus, Liver, Gallbladder, Pancreas, Oral mucosa, Small intestine, Colon, Rectum, Kidney, Epididymis, Fallopian tube, Breast, Cervix (uterine), Placenta, Skin | Thyroid gland, Parathyroid gland, Adrenal gland, Stomach, Duodenum, Seminal vesicle | .. | Indapamide | .. |
| X-linked Inhibitor of Apoptosis | XIAP | Intrinsic pathway for apoptosis, TNF signaling, Copper homeostasis, Nucleotide-binding oligomerization domain, No/cGMP/PKG mediated neuroprotection, Apoptosis modulation and signaling, Regulated necrosis, TCF dependent signaling in response to WNT, Focal adhesion | 140 | Lung, Pancreas, Epididymis, Prostate, Vagina, Endometrium, Cervix, uterine, Smooth muscle, Lymph node, Tonsil | Cerebral cortex, Cerebellum, Hippocampus, Thyroid gland, Parathyroid gland, Nasopharynx, Bronchus, Oral mucos, aEsophagus, Stomach, Duodenum, Small intestine, Colon, Rectum, Liver, Kidney, Urinary bladder, Seminal vesicle, Fallopian tube, Breast, Soft tissue, Skin, Appendix | Adrenal gland, Salivary gland, Gallbladder, Testis, Heart muscle | .. | Cytarabine, Etoposide, Fludarabine | YES |

**References:**

1. Vargas LM, Cerpa W, Muñoz FJ, Zanlungo S, Alvarez AR. Amyloid-β oligomers synaptotoxicity: The emerging role of EphA4/c-Abl signaling in Alzheimer's disease. Biochim Biophys Acta 2018; **1864**: 1148-1159.

2. Mineur YS, Obayemi A, Wigestrand MB, Fote GM, Calarco CA, Li AM, Picciotto MR. Cholinergic signaling in the hippocampus regulates social stress resilience and anxiety- and depression-like behavior. Proc Natl Acad Sci U S A 2013; **110**: 3573-3578.

3. Matrone C, Iannuzzi F, Annunziato L. The Y(682)ENPTY(687) motif of APP: Progress and insights toward a targeted therapy for Alzheimer's disease patients. Ageing Res Rev 2019; **52**: 120-128.

4. Weiss JB, Weber S, Marzulla T, Raber J. Pharmacological inhibition of Anaplastic Lymphoma Kinase rescues spatial memory impairments in Neurofibromatosis 1 mutant mice. Behav Brain Res 2017; **332**: 337-342.

5. Henningsson S, Jonsson L, Ljunggren E, Westberg L, Gillberg C, Råstam M, Anckarsäter H, Nygren G, Landén M, Thuresson K, Betancur C, Leboyer M, Gillberg C, Eriksson E, Melke J. Possible association between the androgen receptor gene and autism spectrum disorder. Psychoneuroendocrinol 2009; **34**: 752-761.

6. Raber J. AR, apoE, and cognitive function. Horm Behav 2008; **53**: 706-715.

7. Carr JS, Bonham LW, Morgans AK, Ryan CJ, Yokoyama JS, Geier EG, AsDNI. Genetic variation in the androgen receptor and measures of plasma testosterone levels suggest androgen dysfunction in Alzheimer’s disease. Front Neurosci 2018; **12**.

8. Pizzamiglio L, Focchi E, Murru L, Tamborini M, Passafaro M, Menna E, Matteoli M, Antonucci F. New role of ATM in controlling GABAergic tone during development. Cereb Cortex 2016; **26**: 3879-3888.

9. Krohn M, Bracke A, Avchalumov Y, Schumacher T, Hofrichter J, Paarmann K, Fröhlich C, Lange C, Brüning T, von Bohlen und Halbach O, Pahnke J. Accumulation of murine amyloid-β mimics early Alzheimer’s disease. Brain 2015; **138**: 2370-2382.

10. Stokes MP, Rush J, MacNeill J, Ren JM, Sprott K, Nardone J, Yang V, Beausoleil SA, Gygi SP, Livingstone M, Zhang H, Polakiewicz RD, Comb MJ. Profiling of UV-induced ATM/ATR signaling pathways. Proc Natl Acad Sci U S A 2007; **104**: 19855-19860.

11. Yamaguchi K, Shioda N, Yabuki Y, Zhang C, Han F, Fukunaga K. SA4503, a potent Sigma-1 receptor ligand, ameliorates synaptic abnormalities and cognitive dysfunction in a mouse model of ATR-X Syndrome. Int J Mol Sci 2018; **19**: 2811.

12. Crupi R, Cambiaghi M, Spatz L, Hen R, Thorn M, Friedman E, Vita G, Battaglia F. Reduced adult neurogenesis and altered emotional behaviors in autoimmune-prone B-cell activating factor transgenic mice. Biol Psychiatry 2010; **67**: 558-566.

13. Zhang C, Wu Z, Hong W, Wang Z, Peng D, Chen J, Yuan C, Yu S, Xu L, Fang Y. Influence of BCL2 gene in major depression susceptibility and antidepressant treatment outcome. J Affect Dis 2014; **155**: 288-294.

14. Gatta L, Cardinale A, Wannenes F, Consoli C, Armani A, Molinari F, Mammi C, Stocchi F, Torti M, Rosano GMC, Fini M. Peripheral blood mononuclear cells from mild cognitive impairment patients show deregulation of Bax and Sod1 mRNAs. Neurosci Lett 2009; **453**: 36-40.

15. Bitencourt RM, Guerra de Souza AC, Bicca MA, Pamplona FA, de Mello N, Passos GF, Medeiros R, Takahashi RN, Calixto JB, Prediger RD. Blockade of hippocampal bradykinin B1 receptors improves spatial learning and memory deficits in middle-aged rats. Behav Brain Res 2017; **316**: 74-81.

16. Amare AT, Schubert KO, Klingler-Hoffmann M, Cohen-Woods S, Baune BT. The genetic overlap between mood disorders and cardiometabolic diseases: a systematic review of genome wide and candidate gene studies. Transl Psychiatry 2017; **7**: e1007.

17. Hashimoto R, Okada T, Kato T, Kosuga A, Tatsumi M, Kamijima K, Kunugi H. The breakpoint cluster region gene on chromosome 22q11 is associated with bipolar disorder. Biol Psychiatry 2005; **57**: 1097-1102.

18. Suberbielle E, Djukic B, Evans M, Kim DH, Taneja P, Wang X, Finucane M, Knox J, Ho K, Devidze N, Masliah E, Mucke L. DNA repair factor BRCA1 depletion occurs in Alzheimer brains and impairs cognitive function in mice. Nature Comm 2015; **6**: 8897.

19. Frappart PO, Lee Y, Lamont J, McKinnon PJ. BRCA2 is required for neurogenesis and suppression of medulloblastoma. EMBO J 2007; **26**: 2732-2742.

20. Sokolow S, Li X, Chen L, Taylor KD, Rotter JI, Rissman RA, Aisen PS, Apostolova LG. Deleterious effect of butyrylcholinesterase K-variant in Donepezil Treatment of mild cognitive impairment. J Alzheimers Dis 2017; **56**: 229-237.

21. De Beaumont L, Pelleieux S, Lamarre-Theroux L, Dea D, Poirier J, Alzheimer's disease cooperative S. butyrylcholinesterase K and apolipoprotein E-varepsilon4 reduce the age of onset of Alzheimer's disease, Accelerate cognitive decline, and modulate Donepezil Response in mild cognitively impaired subjects. J Alzheimers Dis 2016; **54**: 913-922.

22. Lee H-g, Casadesus G, Nunomura A, Zhu X, Castellani RJ, Richardson SL, Perry G, Felsher DW, Petersen RB, Smith MA. The neuronal expression of MYC causes a neurodegenerative phenotype in a novel transgenic mouse. Am J Pathol 2009; **174**: 891-897.

23. Zhang B, Chen X, Lv Y, Wu X, Gui L, Zhang Y, Qiu J, Song G, Yao W, Wan L, Zhang C. Cdh1 overexpression improves emotion and cognitive-related behaviors via regulating hippocampal neuroplasticity in global cerebral ischemia rats. Neurochem Int 2019; **124**: 225-237.

24. Scarr E. Muscarinic Receptors in Psychiatric Disorders – Can We Mimic ‘Health’? Neurosignals 2009; **17**: 298-310.

25. Mura E, Zappettini S, Preda S, Biundo F, Lanni C, Grilli M, Cavallero A, Olivero G, Salamone A, Govoni S, Marchi M. Dual effect of beta-amyloid on α7 and α4β2 nicotinic receptors controlling the release of glutamate, aspartate and GABA in rat hippocampus. PLoS One 2012; **7**: e29661-e29661.

26. Dyrvig M, Mikkelsen JD, Lichota J. DNA methylation regulates CHRNA7 transcription and can be modulated by valproate. Neurosci Lett 2019; **704**: 145-152.

27. Michels M, Danieslki LG, Vieira A, Florentino D, Dall'Igna D, Galant L, Sonai B, Vuolo F, Mina F, Pescador B, Dominguini D, Barichello T, Quevedo J, Dal-Pizzol F, Petronilho F. CD40-CD40 ligand pathway is a major component of acute neuroinflammation and contributes to long-term cognitive dysfunction after sepsis. Mol Med 2015; **21**: 219-226.

28. Ye X, Zhou W, Zhang J, for Alzheimer’s Disease Neuroimaging I. Association of CSF CD40 levels and synaptic degeneration across the Alzheimer's disease spectrum. Neurosci Lett 2019; **694**: 41-45.

29. Ano Y, Ohya R, Kondo K, Nakayama H. Iso-α-acids, hop-derived bitter components of beer, attenuate age-related inflammation and cognitive decline. Frontiers in aging neuroscience 2019; **11**: 16-16.

30. Keeney JTR, Swomley AM, Harris JL, Fiorini A, Mitov MI, Perluigi M, Sultana R, Butterfield DA. Cell cycle proteins in brain in mild cognitive impairment: insights into progression to Alzheimer disease. Neurotox Res 2012; **22**: 220-230.

31. Wu J, Zhao Z, Sabirzhanov B, Stoica BA, Kumar A, Luo T, Skovira J, Faden AI. Spinal cord injury causes brain inflammation associated with cognitive and affective changes: role of cell cycle pathways. J Neurosci 2014; **34**: 10989-11006.

32. Kim H, Kwon Y-A, Ahn IS, Kim S, Kim S, Jo SA, Kim DK. Overexpression of cell cycle proteins of peripheral lymphocytes in patients with Alzheimer's disease. Psychiatry Investig 2016; **13**: 127-134.

33. Gelosa P, Colazzo F, Tremoli E, Sironi L, Castiglioni L. Cysteinyl leukotrienes as potential pharmacological targets for cerebral diseases. Mediators Inflamm 2017; **2017**: 3454212-3454212.

34. Yang Z, Li H, Tang Y, Liu X, Liao Q, Fan C, Wang S. CYP1B1 deiciency ameliorates learning and memory deficits caused by high fat diet in mice. Am J Transl Res 2019; **11**: 2194-2206.

35. Anthoni H, Sucheston LE, Lewis BA, Tapia-Páez I, Fan X, Zucchelli M, Taipale M, Stein CM, Hokkanen M-E, Castrén E, Pennington BF, Smith SD, Olson RK, Tomblin JB, Schulte-Körne G, Nöthen M, Schumacher J, Müller-Myhsok B, Hoffmann P, Gilger JW, Hynd GW, Nopola-Hemmi J, Leppanen PHT, Lyytinen H, Schoumans J, Nordenskjöld M, Spencer J, Stanic D, Boon WC, Simpson E, Mäkelä S, Gustafsson J-Å, Peyrard-Janvid M, Iyengar S, Kere J. The aromatase gene CYP19A1: several genetic and functional lines of evidence supporting a role in reading, speech and language. Behav Genet 2012; **42**: 509-527.

36. Fabbri C, Serretti A. Pharmacogenetics of major depressive disorder: top genes and pathways toward clinical applications. Curr Psychiatry Rep 2015; **17**: 50.

37. D’empaire I, Guico-Pabia CJ, Preskorn SH. Antidepressant treatment and altered CYP2D6 activity: are pharmacokinetic variations clinically relevant? J Psychiatr Pract 2011; **17**: 330-339.

38. Tang J, Yu W, Chen S, Gao Z, Xiao B. Microglia polarization and endoplasmic reticulum stress in chronic social defeat stress induced depression mouse. Neurochem Res 2018; **43**: 985-994.

39. Jackson AR, Shah A, Kumar A. Methamphetamine alters the normal progression by inducing cell cycle arrest in astrocytes. PLoS One 2014; **9**: e109603-e109603.

40. Veldic M, Caruncho HJ, Liu WS, Davis J, Satta R, Grayson DR, Guidotti A, Costa E. DNA-methyltransferase 1 mRNA is selectively overexpressed in telencephalic GABAergic interneurons of schizophrenia brains. Proc Natl Acad Sci U S A 2004; **101**: 348-353.

41. Veldic M, Guidotti A, Maloku E, Davis JM, Costa E. In psychosis, cortical interneurons overexpress DNA-methyltransferase 1. Proc Natl Acad Sci U S A 2005; **102**: 2152-2157.

42. Baets J, Duan X, Wu Y, Smith G, Seeley WW, Mademan I, McGrath NM, Beadell NC, Khoury J, Botuyan M-V, Mer G, Worrell GA, Hojo K, DeLeon J, Laura M, Liu Y-T, Senderek J, Weis J, Van den Bergh P, Merrill SL, Reilly MM, Houlden H, Grossman M, Scherer SS, De Jonghe P, Dyck PJ, Klein CJ. Defects of mutant DNMT1 are linked to a spectrum of neurological disorders. Brain 2015; **138**: 845-861.

43. Ji S, Ding X, Ji J, Wu H, Sun R, Li X, Zhang L, Tian Y. Cranial irradiation inhibits hippocampal neurogenesis via DNMT1 and DNMT3A. Oncol Lett 2018; **15**: 2899-2904.

44. Chouliaras L, Kenis G, Visser PJ, Scheltens P, Tsolaki M, Jones RW, Kehoe PG, Graff C, Girtler NG, Wallin ÅK, Rikkert MO, Spiru L, Elias-Sonnenschein LS, Ramakers IH, Pishva E, Os Jv, Steinbusch HW, Verhey FR, Hove DLvd, Rutten BP. DNMT3A moderates cognitive decline in subjects with mild cognitive impairment: replicated evidence from two mild cognitive impairment cohorts. Epigenomics 2015; **7**: 533-537.

45. Van Esch H, Colnaghi R, Freson K, Starokadomskyy P, Zankl A, Backx L, Abramowicz I, Outwin E, Rohena L, Faulkner C, Leong GM, Newbury-Ecob RA, Challis RC, Õunap K, Jaeken J, Seuntjens E, Devriendt K, Burstein E, Low KJ, O’Driscoll M. Defective DNA polymerase α-primase leads to X-linked intellectual disability associated with severe growth retardation, microcephaly, and hypogonadism. Am J Human Genet 2019; **104**: 957-967.

46. Meng LX, He MY, Xiong M, Zhang XY, Nie SK, Xiong J, Hu D, Zhang ZH, Mao L, Zhang ZT. 2 ',3 '-Dideoxycytidine, a DNA polymerase-beta inhibitor, reverses memory deficits in a mouse model of Alzheimer's disease. J Alzheimers Dis 2019; **67**: 515-525.

47. Gao S, Zhang X, Song Q, Liu J, Ji X, Wang P. POLD1 deficiency is involved in cognitive function impairment in AD patients and SAMP8 mice. Biomed Pharmacother 2019; **114**: 108833.

48. King IF, Yandava CN, Mabb AM, Hsiao JS, Huang H-S, Pearson BL, Calabrese JM, Starmer J, Parker JS, Magnuson T, Chamberlain SJ, Philpot BD, Zylka MJ. Topoisomerases facilitate transcription of long genes linked to autism. Nature 2013; **501**: 58-62.

49. Nkam I, Ramoz N, Breton F, Mallet J, Gorwood P, Dubertret C. Impact of DRD2/ANKK1 and COMT Polymorphisms on Attention and Cognitive Functions in Schizophrenia. PLoS One 2017; **12**: e0170147-e0170147.

50. Zou Y-F, Wang F, Feng X-L, Li W-F, Tian Y-H, Tao J-H, Pan F-M, Huang F. Association of DRD2 gene polymorphisms with mood disorders: A meta-analysis. Journal of Affective Disorders 2012; **136**: 229-237.

51. Lencer R, Bishop JR, Harris MSH, Reilly JL, Patel S, Kittles R, Prasad KM, Nimgaonkar VL, Keshavan MS, Sweeney JA. Association of variants in DRD2 and GRM3 with motor and cognitive function in first-episode psychosis. Eur Arch Psychiatry Clin Neurosci 2014; **264**: 345-355.

52. Ting JH, Marks DR, Schleidt SS, Wu JN, Zyskind JW, Lindl KA, Blendy JA, Pierce RC, Jordan-Sciutto KL. Targeted gene mutation of E2F1 evokes age-dependent synaptic disruption and behavioral deficits. J Neurochem 2014; **129**: 850-863.

53. Zammit AR, Katz MJ, Zimmerman ME, Bitzer M, Lipton RB. Low eGFR is associated with dysexecutive and amnestic mild cognitive impairment. Alzheimers Dement 2015; **1**: 152-159.

54. Hsu W-Y, Lane H-Y, Lin C-H. Medications used for cognitive enhancement in patients with schizophrenia, bipolar disorder, Alzheimer's disease, and Parkinson's disease. Front Psychiatry 2018; **9**: 91-91.

55. Mao D, Reuter CM, Ruzhnikov MRZ, Beck AE, Farrow EG, Emrick LT, Rosenfeld JA, Mackenzie KM, Robak L, Wheeler MT, Burrage LC, Jain M, Liu P, Calame D, Küry S, Sillesen M, Schmitz-Abe K, Tonduti D, Spaccini L, Iascone M, Genetti CA, Graf M, Tran A, Alejandro M, Lee BH, Thiffault I, Agrawal PB, Bernstein JA, Bellen HJ, Chao H-T. De novo EIF2AK1 and EIF2AK2 variants are associated with developmental delay, leukoencephalopathy, and neurologic decompensation. BioRxiv 2019: 757039.

56. Frolinger T, Smith C, Cobo CF, Sims S, Brathwaite J, Boer Sd, Huang J, Pasinetti GM. Dietary polyphenols promote resilience against sleep deprivation–induced cognitive impairment by activating protein translation. FASEB J 2018; **32**: 5390-5404.

57. Huynh TN, Shah M, Koo SY, Faraud KS, Santini E, Klann E. eIF4E/Fmr1 double mutant mice display cognitive impairment in addition to ASD-like behaviors. Neurobiol Dis 2015; **83**: 67-74.

58. Le-Niculescu H, Kurian SM, Yehyawi N, Dike C, Patel SD, Edenberg HJ, Tsuang MT, Salomon DR, Nurnberger Jr JI, Niculescu AB. Identifying blood biomarkers for mood disorders using convergent functional genomics. Mol Psychiatry 2008; **14**: 156.

59. Jürgenson M, Aonurm-Helm A, Zharkovsky A. Partial reduction in neural cell adhesion molecule (NCAM) in heterozygous mice induces depression-related behaviour without cognitive impairment. Brain Res 2012; **1447**: 106-118.

60. Khonsari RH, Delezoide A-L, Kang W, Hébert JM, Bessières B, Bodiguel V, Collet C, Legeai-Mallet L, Sharpe PT, Fallet-Bianco C. Central nervous system malformations and deformations in FGFR2-related craniosynostosis. Am J Med Genet A 2012; **158A**: 2797-2806.

61. Dwivedi Y, Rizavi HS, Conley RR, Roberts RC, Tamminga CA, Pandey GN. Altered gene expression of brain-derived neurotrophic factor and receptor Tyrosine Kinase B in postmortem brain of suicide subjects. Arch Gen Psychiatry 2003; **60**: 804-815.

62. Choi M, Wang SE, Ko SY, Kang HJ, Chae SY, Lee SH, Kim Y-S, Duman RS, Son H. Overexpression of human GATA-1 and GATA-2 interferes with spine formation and produces depressive behavior in rats. PLoS One 2014; **9**: e109253-e109253.

63. Kerner B, Jasinska AJ, DeYoung J, Almonte M, Choi O-W, Freimer NB. Polymorphisms in the GRIA1 gene region in psychotic bipolar disorder. American Journal of Medical Genetics Part B: Neuropsychiatr Genet 2009; **150B**: 24-32.

64. Galyamina AG, Kovalenko IL, Smagin DA, Kudryavtseva NN. Changes in the expression of neurotransmitter system genes in the ventral tegmental area in depressed mice: RNA-SEQ data. Neurosci Behav Physiol 2018; **48**: 591-602.

65. Mignogna ML, Giannandrea M, Gurgone A, Fanelli F, Raimondi F, Mapelli L, Bassani S, Fang H, Van Anken E, Alessio M, Passafaro M, Gatti S, Esteban JA, Huganir R, D'Adamo P. The intellectual disability protein RAB39B selectively regulates GluA2 trafficking to determine synaptic AMPAR composition. Nature Comm 2015; **6**: 6504-6504.

66. Utge S, Kronholm E, Partonen T, Soronen P, Ollila HM, Loukola A, Perola M, Salomaa V, Porkka-Heiskanen T, Paunio T. Shared genetic background for regulation of mood and sleep: association of GRIA3 with sleep duration in healthy Finnish women. Sleep 2011; **34**: 1309-1316.

67. Sequeira A, Mamdani F, Ernst C, Vawter MP, Bunney WE, Lebel V, Rehal S, Klempan T, Gratton A, Benkelfat C, Rouleau GA, Mechawar N, Turecki G. Global brain gene expression analysis links glutamatergic and GABAergic alterations to suicide and major depression. PLoS One 2009; **4**: e6585-e6585.

68. Zarate C, Jr., Machado-Vieira R, Henter I, Ibrahim L, Diazgranados N, Salvadore G. Glutamatergic modulators: the future of treating mood disorders? Harv Rev Psychiatry 2010; **18**: 293-303.

69. Gupta SC, Ravikrishnan A, Liu J, Mao Z, Pavuluri R, Hillman BG, Gandhi PJ, Stairs DJ, Li M, Ugale RR, Monaghan DT, Dravid SM. The NMDA receptor GluN2C subunit controls cortical excitatory-inhibitory balance, neuronal oscillations and cognitive function. Sci Rep 2016; **6**: 38321-38321.

70. Javitt DC. Glutamate as a therapeutic target in psychiatric disorders. Mol Psychiatry 2004; **9**: 984-997.

71. Yamamoto H, Hagino Y, Kasai S, Ikeda K. Specific roles of NMDA receptor subunits in mental disorders. Curr Mol Med 2015; **15**: 193-205.

72. Gao L, Gao Y, Xu E, Xie J. Microarray analysis of the major depressive disorder mRNA profile data. Psychiatry Investig 2015; **12**: 388-396.

73. Chai YL, Hilal S, Chong JPC, Ng YX, Liew OW, Xu X, Ikram MK, Venketasubramanian N, Richards AM, Lai MKP, Chen CP. Growth differentiation factor-15 and white matter hyperintensities in cognitive impairment and dementia. Medicine 2016; **95**: e4566-e4566.

74. François M, Leifert WR, Hecker J, Faunt J, Fenech MF. Guanine-quadruplexes are increased in mild cognitive impairment and correlate with cognitive function and chromosomal DNA damage. DNA Repair 2016; **46**: 29-36.

75. Tunc-Ozcan E, Wert SL, Lim PH, Ferreira A, Redei EE. Hippocampus-dependent memory and allele-specific gene expression in adult offspring of alcohol-consuming dams after neonatal treatment with thyroxin or metformin. Mol Psychiatry 2018; **23**: 1643-1651.

76. Martín-Montañez E, Millon C, Boraldi F, Garcia-Guirado F, Pedraza C, Lara E, Santin LJ, Pavia J, Garcia-Fernandez M. IGF-II promotes neuroprotection and neuroplasticity recovery in a long-lasting model of oxidative damage induced by glucocorticoids. Redox Biol 2017; **13**: 69-81.

77. Wilson KE, Demyanovich H, Rubin LH, Wehring HJ, Kilday C, Kelly DL. Relationship of interferon-γ to cognitive function in midlife women with schizophrenia. Psychiatr Quarterly 2018; **89**: 937-946.

78. Pinto EF, Andrade C. Interferon-related depression: A primer on mechanisms, treatment, and prevention of a common clinical problem. Curr Neuropharmacol 2016; **14**: 743-748.

79. Valentine AD, Meyers CA, Kling MA, Richelson E, Hauser P. Mood and cognitive side effects of interferon-alpha therapy. Semin Oncol 1998; **25**: 39-47.

80. Soczynska JK, Kennedy SH, Goldstein BI, Lachowski A, Woldeyohannes HO, McIntyre RS. The effect of tumor necrosis factor antagonists on mood and mental health-associated quality of life: Novel hypothesis-driven treatments for bipolar depression? NeuroToxicol 2009; **30**: 497-521.

81. Barbosa IG, Ferreira RdA, Rocha NP, Mol GC, da Mata Chiaccjio Leite F, Bauer IE, Teixeira AL. Predictors of cognitive performance in bipolar disorder: The role of educational degree and inflammatory markers. J Psychiatr Res 2018; **106**: 31-37.

82. Maes M, Carvalho AF. The compensatory immune-regulatory reflex system (CIRS) in depression and bipolar disorder. Mol Neurobiol 2018; **55**: 8885-8903.

83. Uher R, Nader Perroud, Mandy Y.M. Ng, Joanna Hauser, Neven Henigsberg, Wolfgang Maier, Ole Mors, Anna Placentino, Psy.D., Marcella Rietschel, Daniel Souery, Tina Žagar, Piotr M. Czerski, Borut Jerman, B.Sc., Erik Roj Larsen, Thomas G. Schulze, Astrid Zobel, Sarah Cohen-Woods, Katrina Pirlo, B.Sc., Amy W. Butler, Pierandrea Muglia, Michael R. Barnes, Lathrop M, Anne Farmer, F.R.C.Psych., Gerome Breen, Katherine J. Aitchison, M.R.C.Psych., Ian Craig, Cathryn M. Lewis, Ph.D. , and, Peter McGuffin, F.R.C.P., F.R.C.Psych., Ph.D. Genome-Wide pharmacogenetics of antidepressant response in the GENDEP project. Am J Psychiatry 2010; **167**: 555-564.

84. Gao Q, Camous X, Lu Y-X, Lim M-L, Larbi A, Ng T-P. Novel inflammatory markers associated with cognitive performance: Singapore longitudinal ageing studies. Neurobiol Aging 2016; **39**: 140-146.

85. Stuart MJ, Baune BT. Chemokines and chemokine receptors in mood disorders, schizophrenia, and cognitive impairment: A systematic review of biomarker studies. Neurosci Biobehav Rev 2014; **42**: 93-115.

86. Kesler SR, Noll K, Cahill DP, Rao G, Wefel JS. The effect of IDH1 mutation on the structural connectome in malignant astrocytoma. J Neurooncol 2017; **131**: 565-574.

87. Tapias V, Jainuddin S, Ahuja M, Stack C, Elipenahli C, Vignisse J, Gerges M, Starkova N, Xu H, Starkov AA, Bettendorff L, Hushpulian DM, Smirnova NA, Gazaryan IG, Kaidery NA, Wakade S, Calingasan NY, Thomas B, Gibson GE, Dumont M, Beal MF. Benfotiamine treatment activates the Nrf2/ARE pathway and is neuroprotective in a transgenic mouse model of tauopathy. Hum Mol Genet 2018; **27**: 2874-2892.

88. Shirai Y, Fujita Y, Hashimoto R, Ohi K, Yamamori H, Yasuda Y, Ishima T, Suganuma H, Ushida Y, Takeda M, Hashimoto K. Dietary intake of sulforaphane-rich broccoli sprout extracts during juvenile and adolescence can prevent phencyclidine-induced cognitive deficits at adulthood. PLoS One 2015; **10**: e0127244-e0127244.

89. Jakovcevski M, Ruan H, Shen EY, Dincer A, Javidfar B, Ma Q, Peter CJ, Cheung I, Mitchell AC, Jiang Y, Lin CL, Pothula V, Stewart AF, Ernst P, Yao W-D, Akbarian S. Neuronal Kmt2a/Mll1 histone methyltransferase is essential for prefrontal synaptic plasticity and working memory. J Neurosci 2015; **35**: 5097-5108.

90. Delport A, Harvey BH, Petzer A, Petzer JP. Methylene blue analogues with marginal monoamine oxidase inhibition retain antidepressant-like activity. ACS ChemNeurosci 2018; **9**: 2917-2928.

91. Eisner P, Klasen M, Wolf D, Zerres K, Eggermann T, Eisert A, Zvyagintsev M, Sarkheil P, Mathiak KA, Zepf F, Mathiak K. Cortico-limbic connectivity in MAOA-L carriers is vulnerable to acute tryptophan depletion. Human Brain Mapping 2017; **38**: 1622-1635.

92. Moriguchi S, Wilson AA, Miler L, Rusjan PM, Vasdev N, Kish SJ, Rajkowska G, Wang J, Bagby M, Mizrahi R, Varughese B, Houle S, Meyer JH. Monoamine Oxidase B total distribution volume in the prefrontal cortex of major depressive disorder. JAMA Psychiatry 2019; **76**: 634-641.

93. Deo AJ, Huang Y-y, Hodgkinson CA, Xin Y, Oquendo MA, Dwork AJ, Arango V, Brent DA, Goldman D, Mann JJ, Haghighi F. A large-scale candidate gene analysis of mood disorders: evidence of neurotrophic tyrosine kinase receptor and opioid receptor signaling dysfunction. Psychiatr Genet 2013; **23**: 47-55.

94. Zarbato GF, de Souza Goldim MP, Giustina AD, Danielski LG, Mathias K, Florentino D, de Oliveira Junior AN, da Rosa N, Laurentino AO, Trombetta T, Gomes ML, Steckert AV, Moreira AP, Schuck PF, Fortunato JJ, Barichello T, Petronilho F. Dimethyl fumarate limits neuroinflammation and oxidative stress and improves cognitive impairment after polymicrobial sepsis. Neurotox Res 2018; **34**: 418-430.

95. Hei Y, Chen R, Mao X, Wang J, Long Q, Liu W. Neuregulin1 attenuates cognitive deficits and hippocampal CA1 neuronal apoptosis partly via ErbB4 receptor in a rat model of chronic cerebral hypoperfusion. Behav Brain Res 2019; **365**: 141-149.

96. Dabbah-Assadi F, Alon D, Golani I, Doron R, Kremer I, Beloosesky R, Shamir A. The influence of immune activation at early vs late gestation on fetal NRG1-ErbB4 expression and behavior in juvenile and adult mice offspring. Brain Behav Immun 2019.

97. Feng S, Shi T, Qiu J, Yang H, Wu Y, Zhou W, Wang W, Wu H. Notch1 deficiency in postnatal neural progenitor cells in the dentate gyrus leads to emotional and cognitive impairment. FASEB J 2017; **31**: 4347-4358.

98. Kwapis JL, Alaghband Y, López AJ, Long JM, Li X, Shu G, Bodinayake KK, Matheos DP, Rapp PR, Wood MA. HDAC3-mediated repression of the Nr4a family contributes to age-related impairments in long-term memory. J Neurosci 2019; **39**: 4999-5009.

99. Dwivedi Y, Rizavi HS, Teppen T, Zhang H, Mondal A, Roberts RC, Conley RR, Pandey GN. Lower Phosphoinositide 3-Kinase (PI 3-kinase) activity and differential expression levels of selective catalytic and regulatory PI 3-Kinase subunit isoforms in prefrontal cortex and hippocampus of suicide subjects. Neuropsychopharmacol 2008; **33**: 2324-2340.

100. Passos GF, Figueiredo CP, Prediger RDS, Silva KABS, Siqueira JM, Duarte FS, Leal PC, Medeiros R, Calixto JB. Involvement of phosphoinositide 3-kinase gamma in the neuro-inflammatory response and cognitive impairments induced by beta-amyloid 1-40 peptide in mice. Brain Behav Immun 2010; **24**: 493-501.

101. Premkumar M, Sable T, Dhanwal D, Dewan R. Vitamin D homeostasis, bone mineral metabolism, and seasonal affective disorder during 1 year of Antarctic residence. Arch Osteoporos 2013; **8**: 129-129.

102. Napoli E, Ross-Inta C, Wong S, Hung C, Fujisawa Y, Sakaguchi D, Angelastro J, Omanska-Klusek A, Schoenfeld R, Giulivi C. Mitochondrial dysfunction in Pten haplo-insufficient mice with social deficits and repetitive behavior: interplay between Pten and p53. PLoS One 2012; **7**: e42504-e42504.

103. Richter W, Menniti FS, Zhang H-T, Conti M. PDE4 as a target for cognition enhancement. Expert Opin Ther Targets 2013; **17**: 1011-1027.

104. Titus DJ, Wilson NM, Freund JE, Carballosa MM, Sikah KE, Furones C, Dietrich WD, Gurney ME, Atkins CM. Chronic cognitive dysfunction after traumatic brain injury is improved with a Phosphodiesterase 4B inhibitor. J Neurosci 2016; **36**: 7095-7108.

105. Rutten K, Wallace TL, Works M, Prickaerts J, Blokland A, Novak TJ, Santarelli L, Misner DL. Enhanced long-term depression and impaired reversal learning in phosphodiesterase 4B-knockout (PDE4B−/−) mice. Neuropharmacol 2011; **61**: 138-147.

106. Giorgi M, Modica A, Pompili A, Pacitti C, Gasbarri A. The induction of cyclic nucleotide phosphodiesterase 4 gene (PDE4D) impairs memory in a water maze task. Behav Brain Res 2004; **154**: 99-106.

107. Pu H, Shi Y, Zhang L, Lu Z, Ye Q, Leak RK, Xu F, Ma S, Mu H, Wei Z, Xu N, Xia Y, Hu X, Hitchens TK, Bennett MVL, Chen J. Protease-independent action of tissue plasminogen activator in brain plasticity and neurological recovery after ischemic stroke. Proc Natl Acad Sci 2019; **116**: 9115-9124.

108. Bahi A, Dreyer J-L. Hippocampus-specific deletion of tissue plasminogen activator “tPA” in adult mice impairs depression- and anxiety-like behaviors. Eur Neuropsychopharmacol 2012; **22**: 672-682.

109. Kuiper E, Nelemans A, Luiten P, Nijholt I, Dolga A, Eisel U. KCa2 and KCa3 channels in learning and memory processes, and neurodegeneration. Front Pharmacol 2012; **3**.

110. Beckley EH, Scibelli AC, Finn DA. Progesterone receptor antagonist CDB-4124 increases depression-like behavior in mice without affecting locomotor ability. Psychoneuroendocrinol 2011; **36**: 824-833.

111. Reddy LF, Waltz JA, Green MF, Wynn JK, Horan WP. Probabilistic reversal learning in schizophrenia: stability of deficits and potential causal mechanisms. Schizophr Bull 2016; **42**: 942-951.

112. Gamble-George JC, Baldi R, Halladay L, Kocharian A, Hartley N, Silva CG, Roberts H, Haymer A, Marnett LJ, Holmes A, Patel S. Cyclooxygenase-2 inhibition reduces stress-induced affective pathology. ELife 2016; **5**: e14137.

113. Pitceathly RDS, Smith C, Fratter C, Alston CL, He L, Craig K, Blakely EL, Evans JC, Taylor J, Shabbir Z, Deschauer M, Pohl U, Roberts ME, Jackson MC, Halfpenny CA, Turnpenny PD, Lunt PW, Hanna MG, Schaefer AM, McFarland R, Horvath R, Chinnery PF, Turnbull DM, Poulton J, Taylor RW, Gorman GS. Adults with RRM2B-related mitochondrial disease have distinct clinical and molecular characteristics. Brain 2012; **135**: 3392-3403.

114. Bremner JD, McCaffery P. The neurobiology of retinoic acid in affective disorders. Progr Neuro-Psychopharmacol Biol Psychiatry 2008; **32**: 315-331.

115. Švob Štrac D, Pivac N, Mück-Šeler D. The serotonergic system and cognitive function. Transl Neurosci 2016; **7**: 35-49.

116. Bozorgmehr A, Alizadeh F, Ofogh SN, Hamzekalayi MRA, Herati S, Moradkhani A, Shahbazi A, Ghadirivasfi M. What do the genetic association data say about the high risk of suicide in people with depression? A novel network-based approach to find common molecular basis for depression and suicidal behavior and related therapeutic targets. J Affect Disord 2018; **229**: 463-468.

117. Padmakumar M, Jaeken J, Ramaekers V, Lagae L, Greene D, Thys C, Van Geet C, BioResource N, Stirrups K, Downes K, Turro E, Freson K. A novel missense variant in SLC18A2 causes recessive brain monoamine vesicular transport disease and absent serotonin in platelets. JIMD Rep 2019; **47**: 9-16.

118. Pearson-Leary J, McNay EC. Novel roles for the insulin-regulated glucose transporter-4 in hippocampally dependent memory. J Neurosci 2016; **36**: 11851-11864.

119. Asle-Rousta M, Oryan S, Ahmadiani A, Rahnema M. Activation of sphingosine 1-phosphate receptor-1 by SEW2871 improves cognitive function in Alzheimer's disease model rats. EXCLI J 2013; **12**: 449-461.

120. Agís-Balboa RC, Pinna G, Pibiri F, Kadriu B, Costa E, Guidotti A. Down-regulation of neurosteroid biosynthesis in corticolimbic circuits mediates social isolation-induced behavior in mice. Proc Natl Acad Sci U S A 2007; **104**: 18736-18741.

121. Anckaerts C, van Gastel J, Leysen V, Hinz R, Azmi A, Simoens P, Shah D, Kara F, Langbeen A, Bols P, Laloux C, Prevot V, Verhoye M, Maudsley S, Van der Linden A. Image-guided phenotyping of ovariectomized mice: altered functional connectivity, cognition, myelination, and dopaminergic functionality. Neurobiol Aging 2019; **74**: 77-89.

122. Chatterjee S, Humby T, Davies W. Behavioural and Psychiatric Phenotypes in Men and Boys with X-Linked Ichthyosis: Evidence from a Worldwide Online Survey. PloS one 2016; **11**: e0164417-e0164417.

123. Scaini G, Fries GR, Valvassori SS, Zeni CP, Zunta-Soares G, Berk M, Soares JC, Quevedo J. Perturbations in the apoptotic pathway and mitochondrial network dynamics in peripheral blood mononuclear cells from bipolar disorder patients. Transl Psy 2017; **7**: e1111-e1111.

124. Choubtum L, Witoonpanich P, Hanchaiphiboolkul S, Bhidayasiri R, Jitkritsadakul O, Pongpakdee S, Wetchaphanphesat S, Boonkongchuen P, Pulkes T. Analysis of SCA8, SCA10, SCA12, SCA17 and SCA19 in patients with unknown spinocerebellar ataxia: a Thai multicentre study. BMC Neurol 2015; **15**: 166-166.

125. Gontier G, Iyer M, Shea JM, Bieri G, Wheatley EG, Ramalho-Santos M, Villeda SA. Tet2 rescues age-related regenerative decline and enhances cognitive function in the adult mouse brain. Cell Reports 2018; **22**: 1974-1981.

126. Zhang L, Xue Z, Liu Q, Liu Y, Xi S, Cheng Y, Li J, Yan J, Shen Y, Xiao C, Xie Z, Qiu Z, Jiang H. Disrupted folate metabolism with anesthesia leads to myelination deficits mediated by epigenetic regulation of ERMN. EBioMedicine 2019; **43**: 473-486.

127. Tang C-Z, Yang J-T, Liu Q-H, Wang Y-R, Wang W-S. Up-regulated miR-192-5p expression rescues cognitive impairment and restores neural function in mice with depression via the Fbln2-mediated TGF-β1 signaling pathway. FASEB J 2019; **33**: 606-618.

128. Turner CA, Thompson RC, Bunney WE, Schatzberg AF, Barchas JD, Myers RM, Akil H, Watson SJ. Altered choroid plexus gene expression in major depressive disorder. Front Hum Neurosci 2014; **8**: 238-238.

129. Graae L, Karlsson R, Paddock S. Significant association of estrogen receptor binding site variation with bipolar disorder in females. PLoS One 2012; **7**: e32304-e32304.

130. Krzysztoń-Russjan J, Zielonka D, Jackiewicz J, Kuśmirek S, Bubko I, Klimberg A, Marcinkowski JT, Anuszewska EL. A study of molecular changes relating to energy metabolism and cellular stress in people with Huntington’s disease: looking for biomarkers. J Bioenerg Biomembr 2013; **45**: 71-85.

131. Berthold-Losleben M, Himmerich H. The TNF-alpha system: functional aspects in depression, narcolepsy and psychopharmacology. Curr Neuropharmacol 2008; **6**: 193-202.

132. Hashimoto R, Fujimaki K, Jeong MR, Senatorov VV, Christ L, Leeds P, Chuang DM, Takeda M. Neuroprotective actions of lithium. Seishin Shinkeigaku Zasshi 2003; **105**: 81-86.

133. Cenini G, Sultana R, Memo M, Butterfield DA. Elevated levels of pro-apoptotic p53 and its oxidative modification by the lipid peroxidation product, HNE, in brain from subjects with amnestic mild cognitive impairment and Alzheimer's disease. J Cell Mol Med 2008; **12**: 987-994.

134. Mattson MP, Ashery U. No more brain tangles with DeltaNp73. Trends in Biochem Sci 2009; **34**: 6-8.

135. Glocke M, Lang F, Schaeffeler E, Lang T, Schwab M, Lang UE. Impact of Vitamin D Receptor VDR rs2228570 polymorphism in oldest old. Kidney and Blood Press Res 2013; **37**: 311-322.

136. Ahmadi S, Mirzaei K, Hossein-Nezhad A, Shariati G. Vitamin D receptor FokI genotype may modify the susceptibility to schizophrenia and bipolar mood disorder by regulation of dopamine D1 receptor gene expression. Minerva Med 2012; **103**: 383-391.

137. Heyes S, Pratt WS, Rees E, Dahimene S, Ferron L, Owen MJ, Dolphin AC. Genetic disruption of voltage-gated calcium channels in psychiatric and neurological disorders. Prog Neurobiol 2015; **134**: 36-54.

138. McCaffery JM, Papandonatos GD, Faulconbridge LF, Erar B, Peter I, Wagenknecht LE, Pajewski NM, Anderson A, Wadden TA, Wing RR, Look ARG. Genetic predictors of depressive symptoms in the Look AHEAD Trial. Psychosom Med 2015; **77**: 982-992.

139. Bruce HA, Kochunov P, Paciga SA, Hyde CL, Chen X, Xie Z, Zhang B, Xi HS, O'Donnell P, Whelan C, Schubert CR, Bellon A, Ament SA, Shukla DK, Du X, Rowland LM, O'Neill H, Hong LE. Potassium channel gene associations with joint processing speed and white matter impairments in schizophrenia. Genes Brain Behav 2017; **16**: 515-521.

140. Christie L-A, Su JH, Tu CH, Dick MC, Zhou J, Cotman CW. Differential regulation of inhibitors of apoptosis proteins in Alzheimer's disease brains. Neurobiol Dis 2007; **26**: 165-173.
