# Supplementary material for: Molecular basis of mood and cognitive adverse events elucidated via a combination of pharmacovigilance data mining and functional enrichment analysis
Source: Arch Toxicol. 2020 Jun 5;94(8):2829–45. doi: 10.1007/s00204-020-02788-1 (PMC7395038; doi:10.1007/s00204-020-02788-1)
Supplement: Supplementary file 3 — Supplementary file3 (DOCX 26 kb) [file 204_2020_2788_MOESM3_ESM.docx]

Online Resource 3

**Molecular basis of mood and cognitive adverse events elucidated via a combination of pharmacovigilance data mining and functional enrichment analysis**

Christos Andronis^1,*^, João Pedro Silva^2,*^, Eftychia Lekka^1^, Vassilis Virvilis^1^, Helena Carmo^2^, Konstantina Bampali^3^, Margot Ernst^3^, Yang Hu^4^, Irena Loryan^4^, Jacques Richard^5^, Félix Carvalho^2,#^, Miroslav M. Savić^6,#^

^1^Biovista, 34 Rodopoleos Street, 16777 Athens, Greece

^2^UCIBIO, REQUIMTE, Laboratory of Toxicology, Department of Biological Sciences, Faculty of Pharmacy, University of Porto, 4050-313, Porto, Portugal

^3^Department of Molecular Neurosciences, Medical University of Vienna, Spitalgasse 4, A-1090 Vienna, Austria

^4^Translational PKPD group, Department of Pharmaceutical Biosciences, Associate member of SciLifeLab, Uppsala University, Sweden

^5^Sanofi R&D, 371 avenue Professeur Blayac, Montpellier, 34000 France

^6^Department of Pharmacology, Faculty of Pharmacy, University of Belgrade, Vojvode Stepe 450, 11000 Belgrade, Serbia

*The authors contributed equally to the manuscript.

#Corresponding authors:

Félix Carvalho, UCIBIO, REQUIMTE, Laboratory of Toxicology, Faculty of Pharmacy, University of Porto, Portugal, Tel. +351 220428600, E-mail: felixdc@ff.up.pt; Miroslav Savić, Faculty of Pharmacy, University of Belgrade, Serbia, Tel. +381 113951280, E-mail: miroslav@pharmacy.bg.ac.rs

**Supplementary Table 3** – Pathway enrichment analysis for both mood and cognitive-related targets, performed using g:Profiler, based on Reactome Pathway Analysis. Pathways were ordered according to their adjusted p-value (the higher the p-value, the higher the pathway is enriched). Noteworthy, candidate pathway elements identified as being associated with both mood and cognitive AEs were included in both lists for analysis.

|  | **Pathway ID** | **Pathway Description** | **Adjusted *p-*value** | **Intersections** |
| --- | --- | --- | --- | --- |
| 1 | REAC:R-HSA-438066 | Unblocking of NMDA receptors, glutamate binding and activation | 9.44E+04 | GRIA1,GRIA2,GRIA3,GRIA4,GRIN1,GRIN2A,GRIN2B,GRIN2D,GRIN2C |
| 2 | REAC:R-HSA-112315 | Transmission across Chemical Synapses | 1.80E+07 | ACHE,BCHE,CHRNA4,CHRNA7,CHRNB2,GRIA1,GRIA2,GRIA3,GRIA4,GRIN1,GRIN2A,GRIN2B,GRIN2D,GRIN3A,MAOA,NRG1,HTR3B,SLC18A2,CACNA1B,GRIN2C |
| 3 | REAC:R-HSA-8849932 | Synaptic adhesion-like molecules | 1.12E+08 | GRIA1,GRIA3,GRIA4,GRIN1,GRIN2A,GRIN2B,GRIN2D,GRIN2C |
| 4 | REAC:R-HSA-9620244 | Long-term potentiation | 1.12E+08 | GRIA1,GRIA2,GRIN1,GRIN2A,GRIN2B,GRIN2D,NRG1,GRIN2C |
| 5 | REAC:R-HSA-112316 | Neuronal System | 1.03E+09 | ACHE,BCHE,CHRNA4,CHRNA7,CHRNB2,GRIA1,GRIA2,GRIA3,GRIA4,GRIN1,GRIN2A,GRIN2B,GRIN2D,GRIN3A,MAOA,NRG1,HTR3B,SLC18A2,CACNA1B,KCNQ1,KCNN2,GRIN2C |
| 6 | REAC:R-HSA-6785807 | Interleukin-4 and Interleukin-13 signaling | 8.66E+08 | BCL2,MYC,IL1B,IL10,IL6,IL8,MAOA,PTGS2,S1PR1,BIRC5,TNF,TP53 |
| 7 | REAC:R-HSA-1280215 | Cytokine Signaling in Immune system | 9.56E+08 | TNFSF13B,BCL2,MYC,CD86,EGFR,EIF4E,FLT3,GRIN1,GRIN2B,GRIN2D,IFNG,IFNAR1,IL1B,IL10,IL11,IL2,IL2RA,IL6,IL8,MAOA,NRG1,PIK3CA,PRL,PTGS2,S1PR1,BIRC5,TNF,TP53,EIF2AK2,APP |
| 8 | REAC:R-HSA-442755 | Activation of NMDA receptors and postsynaptic events | 1.10E-06 | GRIA1,GRIA2,GRIA3,GRIA4,GRIN1,GRIN2A,GRIN2B,GRIN2D,GRIN3A,NRG1,GRIN2C |
| 9 | REAC:R-HSA-112314 | Neurotransmitter receptors and postsynaptic signal transmission | 1.20E-06 | CHRNA4,CHRNA7,CHRNB2,GRIA1,GRIA2,GRIA3,GRIA4,GRIN1,GRIN2A,GRIN2B,GRIN2D,GRIN3A,NRG1,HTR3B,GRIN2C |
| 10 | REAC:R-HSA-449147 | Signaling by Interleukins | 2.79E-06 | BCL2,MYC,CD86,EGFR,FLT3,GRIN1,GRIN2B,GRIN2D,IFNG,IL1B,IL10,IL11,IL2,IL2RA,IL6,IL8,MAOA,NRG1,PIK3CA,PTGS2,S1PR1,BIRC5,TNF,TP53,APP |
| 11 | REAC:R-HSA-399710 | Activation of AMPA receptors | 5.54E-06 | GRIA1,GRIA2,GRIA3,GRIA4 |
| 12 | REAC:R-HSA-212436 | Generic Transcription Pathway | 7.89E-06 | ABL1,AR,ATM,ATR,BAX,BDNF,BRCA1,MYC,DDIT3,E2F1,EGFR,ESR1,GATA1,GRIA2,GRIN2A,GRIN2B,H2AFX,IFNG,IL2,IL2RA,KMT2A,NOTCH1,PTEN,PGR,RRM2B,RARA,BIRC5,TP53,VDR,CDK2,CDK4,CDK6,TP73,NR4A1,TBP |
| 13 | REAC:R-HSA-74160 | Gene expression (Transcription) | 3.12E-05 | ABL1,AR,ATM,ATR,BAX,BDNF,BRCA1,MYC,DDIT3,DNMT1,DNMT3A,E2F1,EGFR,ESR1,GATA1,GRIA2,GRIN2A,GRIN2B,H2AFX,IFNG,IL2,IL2RA,KMT2A,NOTCH1,PTEN,PGR,RRM2B,RARA,BIRC5,TET2,TP53,VDR,CDK2,CDK4,CDK6,TP73,NR4A1,TBP |
| 14 | REAC:R-HSA-73857 | RNA Polymerase II Transcription | 8.94E-05 | ABL1,AR,ATM,ATR,BAX,BDNF,BRCA1,MYC,DDIT3,E2F1,EGFR,ESR1,GATA1,GRIA2,GRIN2A,GRIN2B,H2AFX,IFNG,IL2,IL2RA,KMT2A,NOTCH1,PTEN,PGR,RRM2B,RARA,BIRC5,TP53,VDR,CDK2,CDK4,CDK6,TP73,NR4A1,TBP |
| 15 | REAC:R-HSA-3108232 | SUMO E3 ligases SUMOylate target proteins | 1.17E-04 | AR,BRCA1,TOP2A,DNMT1,DNMT3A,TOP1,ESR1,PGR,RARA,BIRC5,TP53,VDR |
| 16 | REAC:R-HSA-2990846 | SUMOylation | 1.71E-04 | AR,BRCA1,TOP2A,DNMT1,DNMT3A,TOP1,ESR1,PGR,RARA,BIRC5,TP53,VDR |
| 17 | REAC:R-HSA-6783783 | Interleukin-10 signaling | 1.97E-04 | CD86,IL1B,IL10,IL6,IL8,PTGS2,TNF |
| 18 | REAC:R-HSA-438064 | Post NMDA receptor activation events | 7.01E-04 | GRIA1,GRIA2,GRIN1,GRIN2A,GRIN2B,GRIN2D,NRG1,GRIN2C |
| 19 | REAC:R-HSA-9617324 | Negative regulation of NMDA receptor-mediated neuronal transmission | 7.87E-04 | GRIN1,GRIN2A,GRIN2B,GRIN2D,GRIN2C |
| 20 | REAC:R-HSA-6794362 | Protein-protein interactions at synapses | 1.63E-03 | GRIA1,GRIA3,GRIA4,GRIN1,GRIN2A,GRIN2B,GRIN2D,GRIN2C |
| 21 | REAC:R-HSA-383280 | Nuclear Receptor transcription pathway | 1.97E-03 | AR,ESR1,PGR,RARA,VDR,NR4A1 |
| 22 | REAC:R-HSA-4090294 | SUMOylation of intracellular receptors | 2.08E-03 | AR,ESR1,PGR,RARA,VDR |
| 23 | REAC:R-HSA-9609736 | Assembly and cell surface presentation of NMDA receptors | 2.28E-03 | GRIN1,GRIN2A,GRIN2B,GRIN2D,GRIN3A,GRIN2C |
| 24 | REAC:R-HSA-416993 | Trafficking of GluR2-containing AMPA receptors | 3.68E-03 | GRIA1,GRIA2,GRIA3,GRIA4 |
| 25 | REAC:R-HSA-162582 | Signal Transduction | 5.54E-03 | ABL1,AR,BCL2,BAX,BDNF,BCR,MYC,CDH1,CHRM1,CD86,CYSLTR1,DRD2,E2F1,EGFR,ESR1,EIF4E,FLT3,GRIN1,GRIN2B,GRIN2D,GNRHR,H2AFX,IGF2,IL2,IL2RA,IL6,IL8,NRG1,NOTCH1,PIK3CA,PTEN,PDE4A,PDE4B,PDE4D,PLAT,PGR,RARA,S1PR1,BIRC5,TNF,TP53,CDK2,CDK4,BDKRB1,PTH,XIAP,APP,NR4A1,TBP,PIK3CG |
| 26 | REAC:R-HSA-109606 | Intrinsic Pathway for Apoptosis | 7.23E-03 | BCL2,BAX,E2F1,TP53,XIAP,TP73 |
| 27 | REAC:R-HSA-453279 | Mitotic G1-G1/S phases | 1.30E-02 | ABL1,MYC,TOP2A,POLA1,E2F1,TYMS,CDK2,CDK4,CDK6 |
| 28 | REAC:R-HSA-9616222 | Transcriptional regulation of granulopoiesis | 2.03E-02 | MYC,E2F1,H2AFX,KMT2A,RARA,CDK2,CDK4 |
| 29 | REAC:R-HSA-2219528 | PI3K/AKT Signaling in Cancer | 2.03E-02 | CD86,EGFR,ESR1,NRG1,PIK3CA,PTEN,NR4A1 |
| 30 | REAC:R-HSA-6803204 | TP53 Regulates Transcription of Genes Involved in Cytochrome C Release | 2.35E-02 | ATM,BAX,TP53,TP73 |
| 31 | REAC:R-HSA-5693532 | DNA Double-Strand Break Repair | 3.20E-02 | ABL1,ATM,ATR,BRCA1,BRCA2,POLD1,H2AFX,TP53,CDK2 |
| 32 | REAC:R-HSA-5693567 | HDR through Homologous Recombination (HRR) or Single Strand Annealing (SSA) | 3.75E-02 | ABL1,ATM,ATR,BRCA1,BRCA2,POLD1,H2AFX,CDK2 |
| 33 | REAC:R-HSA-139915 | Activation of PUMA and translocation to mitochondria | 4.23E-02 | E2F1,TP53,TP73 |
| 34 | REAC:R-HSA-5633008 | TP53 Regulates Transcription of Cell Death Genes | 4.48E-02 | ATM,BAX,BIRC5,TP53,TP73 |
| 35 | REAC:R-HSA-141333 | Biogenic amines are oxidatively deaminated to aldehydes by MAOA and MAOB | 4.98E-02 | MAOA,MAOB |
