# Supplementary material for: Molecular basis of mood and cognitive adverse events elucidated via a combination of pharmacovigilance data mining and functional enrichment analysis
Source: Arch Toxicol. 2020 Jun 5;94(8):2829–45. doi: 10.1007/s00204-020-02788-1 (PMC7395038; doi:10.1007/s00204-020-02788-1)
Supplement: Supplementary file 4 — Supplementary file4 (DOCX 25 kb) [file 204_2020_2788_MOESM4_ESM.docx]

Online Resource 4

**Molecular basis of mood and cognitive adverse events elucidated via a combination of pharmacovigilance data mining and functional enrichment analysis**

Christos Andronis^1,*^, João Pedro Silva^2,*^, Eftychia Lekka^1^, Vassilis Virvilis^1^, Helena Carmo^2^, Konstantina Bampali^3^, Margot Ernst^3^, Yang Hu^4^, Irena Loryan^4^, Jacques Richard^5^, Félix Carvalho^2,#^, Miroslav M. Savić^6,#^

^1^Biovista, 34 Rodopoleos Street, 16777 Athens, Greece

^2^UCIBIO, REQUIMTE, Laboratory of Toxicology, Department of Biological Sciences, Faculty of Pharmacy, University of Porto, 4050-313, Porto, Portugal

^3^Department of Molecular Neurosciences, Medical University of Vienna, Spitalgasse 4, A-1090 Vienna, Austria

^4^Translational PKPD group, Department of Pharmaceutical Biosciences, Associate member of SciLifeLab, Uppsala University, Sweden

^5^Sanofi R&D, 371 avenue Professeur Blayac, Montpellier, 34000 France

^6^Department of Pharmacology, Faculty of Pharmacy, University of Belgrade, Vojvode Stepe 450, 11000 Belgrade, Serbia

*The authors contributed equally to the manuscript.

#Corresponding authors:

Félix Carvalho, UCIBIO, REQUIMTE, Laboratory of Toxicology, Faculty of Pharmacy, University of Porto, Portugal, Tel. +351 220428600, E-mail: felixdc@ff.up.pt; Miroslav Savić, Faculty of Pharmacy, University of Belgrade, Serbia, Tel. +381 113951280, E-mail: miroslav@pharmacy.bg.ac.rs

**Supplementary Table 4** – Pathway enrichment analysis for mood-related CPEs, performed using g:Profiler, based on Reactome Pathway Analysis. Pathways were ordered according to their adjusted *p*-value (the higher the *p*-value, the higher the pathway is enriched). Only CPEs related to pharmaceuticals associated with mood AEs were included in the analysis.

|  | **GO.ID** | **Pathway description** | ***p*-Value** | **Genes** |
| --- | --- | --- | --- | --- |
| 1 | REAC:R-HSA-438066 | Unblocking of NMDA receptors, glutamate binding and activation | 6.48E+02 | GRIA1,GRIA2,GRIA3,GRIA4,GRIN1,GRIN2A,GRIN2B,GRIN2D |
| 2 | REAC:R-HSA-8849932 | Synaptic adhesion-like molecules | 1.96E+06 | GRIA1,GRIA3,GRIA4,GRIN1,GRIN2A,GRIN2B,GRIN2D |
| 3 | REAC:R-HSA-112315 | Transmission across Chemical Synapses | 8.50E+05 | ACHE,CHRNA7,CHRNB2,GRIA1,GRIA2,GRIA3,GRIA4,GRIN1,GRIN2A,GRIN2B,GRIN2D,GRIN3A,MAOA,HTR3B,SLC18A2 |
| 4 | REAC:R-HSA-442755 | Activation of NMDA receptors and postsynaptic events | 1.03E+08 | GRIA1,GRIA2,GRIA3,GRIA4,GRIN1,GRIN2A,GRIN2B,GRIN2D,GRIN3A |
| 5 | REAC:R-HSA-9620244 | Long-term potentiation | 2.92E+08 | GRIA1,GRIA2,GRIN1,GRIN2A,GRIN2B,GRIN2D |
| 6 | REAC:R-HSA-112314 | Neurotransmitter receptors and postsynaptic signal transmission | 3.42E+08 | CHRNA7,CHRNB2,GRIA1,GRIA2,GRIA3,GRIA4,GRIN1,GRIN2A,GRIN2B,GRIN2D,GRIN3A |
| 7 | REAC:R-HSA-6785807 | Interleukin-4 and Interleukin-13 signaling | 1.72E-06 | TP53,BIRC5,BCL2,IL6,PTGS2,IL1B,IL10,MAOA,TNF |
| 8 | REAC:R-HSA-6794362 | Protein-protein interactions at synapses | 7.62E-06 | GRIA1,GRIA3,GRIA4,GRIN1,GRIN2A,GRIN2B,GRIN2D |
| 9 | REAC:R-HSA-416993 | Trafficking of GluR2-containing AMPA receptors | 2.34E-05 | GRIA1,GRIA2,GRIA3,GRIA4 |
| 10 | REAC:R-HSA-438064 | Post NMDA receptor activation events | 1.10E-04 | GRIA1,GRIA2,GRIN1,GRIN2A,GRIN2B,GRIN2D |
| 11 | REAC:R-HSA-8877330 | RUNX1 and FOXP3 control the development of regulatory T lymphocytes (Tregs) | 1.13E-04 | IL2,IFNG,IL2RA |
| 12 | REAC:R-HSA-3108232 | SUMO E3 ligases SUMOylate target proteins | 1.44E-04 | TP53,PGR,BIRC5,VDR,ESR1 |
| 13 | REAC:R-HSA-9609736 | Assembly and cell surface presentation of NMDA receptors | 1.53E-04 | GRIN1,GRIN2A,GRIN2B,GRIN2D,GRIN3A |
| 14 | REAC:R-HSA-2990846 | SUMOylation | 1.71E-04 | TP53,PGR,BIRC5,VDR,ESR1 |
| 15 | REAC:R-HSA-9617324 | Negative regulation of NMDA receptor-mediated neuronal transmission | 2.44E-04 | GRIN1,GRIN2A,GRIN2B,GRIN2D |
| 16 | REAC:R-HSA-399721 | Glutamate binding, activation of AMPA receptors and synaptic plasticity | 5.56E-04 | GRIA1,GRIA2,GRIA3,GRIA4 |
| 17 | REAC:R-HSA-399719 | Trafficking of AMPA receptors | 5.56E-04 | GRIA1,GRIA2,GRIA3,GRIA4 |
| 18 | REAC:R-HSA-4090294 | SUMOylation of intracellular receptors | 5.56E-04 | PGR,VDR,ESR1 |
| 19 | REAC:R-HSA-5633008 | TP53 Regulates Transcription of Cell Death Genes | 7.72E-04 | TP53,BAX,BIRC5 |
| 20 | REAC:R-HSA-6783783 | Interleukin-10 signaling | 1.20E-03 | IL6,PTGS2,IL1B,IL10,TNF |
| 21 | REAC:R-HSA-383280 | Nuclear Receptor transcription pathway | 2.90E-03 | PGR,VDR,ESR1 |
| 22 | REAC:R-HSA-109606 | Intrinsic Pathway for Apoptosis | 4.11E-03 | TP53,BAX,BCL2 |
| 23 | REAC:R-HSA-8878171 | Transcriptional regulation by RUNX1 | 6.31E-03 | IL2,ESR1,GATA1,IFNG,IL2RA |
| 24 | REAC:R-HSA-5684996 | MAPK1/MAPK3 signaling | 7.42E-03 | FLT3,IL2,IL2RA,IL6,GRIN1,GRIN2B,GRIN2D |
| 25 | REAC:R-HSA-9022699 | MECP2 regulates neuronal receptors and channels | 7.71E-03 | GRIA2,GRIN2A,GRIN2B |
| 26 | REAC:R-HSA-442982 | Ras activation upon Ca2+ influx through NMDA receptor | 1.43E-02 | GRIN1,GRIN2B,GRIN2D |
| 27 | REAC:R-HSA-6794361 | Neurexins and neuroligins | 1.68E-02 | GRIN1,GRIN2A,GRIN2B,GRIN2D |
| 28 | REAC:R-HSA-8986944 | Transcriptional Regulation by MECP2 | 1.86E-02 | BDNF,GRIA2,GRIN2A,GRIN2B |
| 29 | REAC:R-HSA-5683057 | MAPK family signaling cascades | 1.96E-02 | FLT3,IL2,IL2RA,IL6,GRIN1,GRIN2B,GRIN2D |
| 30 | REAC:R-HSA-9607240 | FLT3 Signaling | 3.16E-02 | FLT3,IL2,IL2RA,GRIN1,GRIN2B,GRIN2D,PIK3CA |
| 31 | REAC:R-HSA-9006115 | Signaling by NTRK2 (TRKB) | 3.72E-02 | BDNF,GRIN2B,PIK3CA |
| 32 | REAC:R-HSA-442660 | Na+/Cl- dependent neurotransmitter transporters | 4.04E-02 | SLC18A1,SLC6A2,SLC18A2 |
| 33 | REAC:R-HSA-442742 | CREB1 phosphorylation through NMDA receptor-mediated activation of RAS signaling | 4.24E-02 | GRIN1,GRIN2B,GRIN2D |
