# Supplementary material for: Molecular basis of mood and cognitive adverse events elucidated via a combination of pharmacovigilance data mining and functional enrichment analysis
Source: Arch Toxicol. 2020 Jun 5;94(8):2829–45. doi: 10.1007/s00204-020-02788-1 (PMC7395038; doi:10.1007/s00204-020-02788-1)
Supplement: Supplementary file 5 — Supplementary file5 (DOCX 28 kb) [file 204_2020_2788_MOESM5_ESM.docx]

Online Resource 5

**Molecular basis of mood and cognitive adverse events elucidated via a combination of pharmacovigilance data mining and functional enrichment analysis**

Christos Andronis^1,*^, João Pedro Silva^2,*^, Eftychia Lekka^1^, Vassilis Virvilis^1^, Helena Carmo^2^, Konstantina Bampali^3^, Margot Ernst^3^, Yang Hu^4^, Irena Loryan^4^, Jacques Richard^5^, Félix Carvalho^2,#^, Miroslav M. Savić^6,#^

^1^Biovista, 34 Rodopoleos Street, 16777 Athens, Greece

^2^UCIBIO, REQUIMTE, Laboratory of Toxicology, Department of Biological Sciences, Faculty of Pharmacy, University of Porto, 4050-313, Porto, Portugal

^3^Department of Molecular Neurosciences, Medical University of Vienna, Spitalgasse 4, A-1090 Vienna, Austria

^4^Translational PKPD group, Department of Pharmaceutical Biosciences, Associate member of SciLifeLab, Uppsala University, Sweden

^5^Sanofi R&D, 371 avenue Professeur Blayac, Montpellier, 34000 France

^6^Department of Pharmacology, Faculty of Pharmacy, University of Belgrade, Vojvode Stepe 450, 11000 Belgrade, Serbia

*The authors contributed equally to the manuscript.

#Corresponding authors:

Félix Carvalho, UCIBIO, REQUIMTE, Laboratory of Toxicology, Faculty of Pharmacy, University of Porto, Portugal, Tel. +351 220428600, E-mail: felixdc@ff.up.pt; Miroslav Savić, Faculty of Pharmacy, University of Belgrade, Serbia, Tel. +381 113951280, E-mail: miroslav@pharmacy.bg.ac.rs

**Supplementary Table 5** – Pathway enrichment analysis for cognitive-related targets, performed using g:Profiler, based on Reactome Pathway Analysis. Pathways were ordered according to their adjusted p-value (the higher the p-value, the higher the pathway is enriched). Only CPEs related to pharmaceuticals associated with cognitive AEs were included in the analysis.

|  | **GO.ID** | **Description** | ***p*-Value** | **Genes** |
| --- | --- | --- | --- | --- |
| 1 | REAC:R-HSA-8877330 | RUNX1 and FOXP3 control the development of regulatory T lymphocytes (Tregs) | 5.69E-04 | IL2,IFNG,IL2RA |
| 2 | REAC:R-HSA-6803204 | TP53 Regulates Transcription of Genes Involved in Cytochrome C Release | 7.55E-04 | TP53,BAX,ATM |
| 3 | REAC:R-HSA-629594 | Highly calcium permeable postsynaptic nicotinic acetylcholine receptors | 5.46E-03 | CHRNA4,CHRNA7,CHRNB2 |
| 4 | REAC:R-HSA-181431 | Acetylcholine binding and downstream events | 1.19E-02 | CHRNA4,CHRNA7,CHRNB2 |
| 5 | REAC:R-HSA-622327 | Postsynaptic nicotinic acetylcholine receptors | 1.19E-02 | CHRNA4,CHRNA7,CHRNB2 |
| 6 | REAC:R-HSA-139915 | Activation of PUMA and translocation to mitochondria | 1.75E-02 | TP53,E2F1,TP73 |
| 7 | REAC:R-HSA-6804116 | TP53 Regulates Transcription of Genes Involved in G1 Cell Cycle Arrest | 1.79E-02 | TP53,CDK2,E2F1 |
| 8 | REAC:R-HSA-8864260 | Transcriptional regulation by the AP-2 (TFAP2) family of transcription factors | 2.29E-02 | MYC,EGFR,ESR1 |
| 9 | REAC:R-HSA-1362277 | Transcription of E2F targets under negative control by DREAM complex | 4.66E-02 | MYC,TOP2A,E2F1 |
| 10 | REAC:R-HSA-4090294 | SUMOylation of intracellular receptors | 5.28E-05 | PGR,VDR,AR,ESR1 |
| 11 | REAC:R-HSA-5633008 | TP53 Regulates Transcription of Cell Death Genes | 9.21E-05 | TP53,BAX,BIRC5,ATM |
| 12 | REAC:R-HSA-109606 | Intrinsic Pathway for Apoptosis | 2.29E-04 | TP53,BAX,XIAP,BCL2 |
| 13 | REAC:R-HSA-383280 | Nuclear Receptor transcription pathway | 4.92E-04 | PGR,VDR,AR,ESR1 |
| 14 | REAC:R-HSA-1538133 | G0 and Early G1 | 4.11E-03 | MYC,TOP2A,CDK2,E2F1 |
| 15 | REAC:R-HSA-5685938 | HDR through Single Strand Annealing (SSA) | 4.69E-03 | ABL1,ATM,ATR,BRCA1 |
| 16 | REAC:R-HSA-5693616 | Presynaptic phase of homologous DNA pairing and strand exchange | 6.39E-03 | ATM,ATR,BRCA1,BRCA2 |
| 17 | REAC:R-HSA-5693579 | Homologous DNA Pairing and Strand Exchange | 8.62E-03 | ATM,ATR,BRCA1,BRCA2 |
| 18 | REAC:R-HSA-109581 | Apoptosis | 2.98E-02 | TP53,BAX,XIAP,BCL2 |
| 19 | REAC:R-HSA-5357801 | Programmed Cell Death | 3.19E-02 | TP53,BAX,XIAP,BCL2 |
| 20 | REAC:R-HSA-8878166 | Transcriptional regulation by RUNX2 | 3.35E-02 | BAX,ABL1,AR,ESR1 |
| 21 | REAC:R-HSA-114452 | Activation of BH3-only proteins | 4.13E-02 | TP53,BCL2,E2F1,TP73 |
| 22 | REAC:R-HSA-6796648 | TP53 Regulates Transcription of DNA Repair Genes | 4.44E-02 | TP53,ATM,ATR,BRCA1 |
| 23 | REAC:R-HSA-2559585 | Oncogene Induced Senescence | 4.62E-02 | TP53,E2F1,CDK4,CDK6 |
| 24 | REAC:R-HSA-9617324 | Negative regulation of NMDA receptor-mediated neuronal transmission | 3.12E-04 | GRIN1,GRIN2A,GRIN2B,GRIN2D,GRIN2C |
| 25 | REAC:R-HSA-438066 | Unblocking of NMDA receptors | -- | GRIN1,GRIN2A,GRIN2B,GRIN2D,GRIN2C |
| 26 | REAC:R-HSA-8849932 | Synaptic adhesion-like molecules | 4.06E-04 | GRIN1,GRIN2A,GRIN2B,GRIN2D,GRIN2C |
| 27 | REAC:R-HSA-5685942 | HDR through Homologous Recombination (HRR) | 2.55E-03 | ATM,POLD1,ATR,BRCA1,BRCA2 |
| 28 | REAC:R-HSA-6783783 | Interleukin-10 signaling | 7.66E-03 | IL6,IL8,CD86,IL1B,TNF |
| 29 | REAC:R-HSA-6804756 | Regulation of TP53 Activity through Phosphorylation | 1.07E-02 | TP53,ATM,CDK2,ATR,BRCA1 |
| 30 | REAC:R-HSA-8939211 | ESR-mediated signaling | 2.62E-02 | PGR,BCL2,MYC,EGFR,ESR1 |
| 31 | REAC:R-HSA-5693565 | Recruitment and ATM-mediated phosphorylation of repair and signaling proteins at DNA double strand breaks | 3.35E-02 | TP53,ABL1,ATM,BRCA1,H2AFX |
| 32 | REAC:R-HSA-5693606 | DNA Double Strand Break Response | 3.56E-02 | TP53,ABL1,ATM,BRCA1,H2AFX |
| 33 | REAC:R-HSA-9620244 | Long-term potentiation | 9.59E-06 | GRIN1,GRIN2A,GRIN2B,GRIN2D,NRG1,GRIN2C |
| 34 | REAC:R-HSA-9609736 | Assembly and cell surface presentation of NMDA receptors | 7.92E-04 | GRIN1,GRIN2A,GRIN2B,GRIN2D,GRIN3A,GRIN2C |
| 35 | REAC:R-HSA-912446 | Meiotic recombination | 2.16E-02 | ATM,CDK2,BRCA1,BRCA2,H2AFX,CDK4 |
| 36 | REAC:R-HSA-438064 | Post NMDA receptor activation events | 2.76E-02 | GRIN1,GRIN2A,GRIN2B,GRIN2D,NRG1,GRIN2C |
| 37 | REAC:R-HSA-2219528 | PI3K/AKT Signaling in Cancer | 4.63E-02 | EGFR,ESR1,PTEN,CD86,NRG1,NR4A1 |
| 38 | REAC:R-HSA-5693567 | HDR through Homologous Recombination (HRR) or Single Strand Annealing (SSA) | 3.25E-04 | ABL1,ATM,POLD1,CDK2,ATR,BRCA1,BRCA2 |
| 39 | REAC:R-HSA-5693538 | Homology Directed Repair | 4.39E-04 | ABL1,ATM,POLD1,CDK2,ATR,BRCA1,BRCA2 |
| 40 | REAC:R-HSA-8878171 | Transcriptional regulation by RUNX1 | 1.21E-03 | ABL1,IL2,ESR1,GATA1,IFNG,IL2RA,KMT2A |
| 41 | REAC:R-HSA-9616222 | Transcriptional regulation of granulopoiesis | 2.12E-03 | MYC,KMT2A,CDK2,E2F1,H2AFX,RARA,CDK4 |
| 42 | REAC:R-HSA-442755 | Activation of NMDA receptors and postsynaptic events | 6.39E-03 | GRIN1,GRIN2A,GRIN2B,GRIN2D,GRIN3A,NRG1,GRIN2C |
| 43 | REAC:R-HSA-1500620 | Meiosis | 1.28E-02 | ATM,CDK2,ATR,BRCA1,BRCA2,H2AFX,CDK4 |
| 44 | REAC:R-HSA-1474165 | Reproduction | 4.58E-02 | ATM,CDK2,ATR,BRCA1,BRCA2,H2AFX,CDK4 |
| 45 | REAC:R-HSA-5693532 | DNA Double-Strand Break Repair | 1.03E-04 | TP53,ABL1,ATM,POLD1,CDK2,ATR,BRCA1,BRCA2 |
| 46 | REAC:R-HSA-5684996 | MAPK1/MAPK3 signaling | 4.92E-02 | IL2,EGFR,IL2RA,IL6,GRIN1,GRIN2B,GRIN2D,NRG1 |
| 47 | REAC:R-HSA-3108232 | SUMO E3 ligases SUMOylate target proteins | 8.81E+06 | TP53,PGR,BIRC5,VDR,AR,TOP2A,DNMT1,DNMT3A,ESR1 |
| 48 | REAC:R-HSA-2990846 | SUMOylation | 1.20E+08 | TP53,PGR,BIRC5,VDR,AR,TOP2A,DNMT1,DNMT3A,ESR1 |
| 49 | REAC:R-HSA-6785807 | Interleukin-4 and Interleukin-13 signaling | 4.69E-05 | TP53,BIRC5,BCL2,MYC,IL6,IL8,IL1B,S1PR1,TNF |
| 50 | REAC:R-HSA-453279 | Mitotic G1-G1/S phases | 8.89E-04 | ABL1,POLA1,MYC,TOP2A,TYMS,CDK2,E2F1,CDK4,CDK6 |
| 51 | REAC:R-HSA-112314 | Neurotransmitter receptors and postsynaptic signal transmission | 1.57E-03 | CHRNA4,CHRNA7,CHRNB2,GRIN1,GRIN2A,GRIN2B,GRIN2D,GRIN3A,NRG1 |
| 52 | REAC:R-HSA-2559583 | Cellular Senescence | 7.69E-03 | TP53,ATM,IL6,IL8,CDK2,E2F1,H2AFX,CDK4,CDK6 |
| 53 | REAC:R-HSA-5683057 | MAPK family signaling cascades | 2.37E-02 | IL2,MYC,EGFR,IL2RA,IL6,GRIN1,GRIN2B,GRIN2D,NRG1 |
| 54 | REAC:R-HSA-73894 | DNA Repair | 9.21E-03 | TP53,ABL1,ATM,POLD1,CDK2,ATR,BRCA1,BRCA2,POLB,H2AFX |
| 55 | REAC:R-HSA-112315 | Transmission across Chemical Synapses | 2.26E-04 | ACHE,BCHE,CHRNA4,CHRNA7,CHRNB2,GRIN1,GRIN2A,GRIN2B,GRIN2D,GRIN3A,NRG1,CACNA1B |
