# Supplementary material for: Molecular basis of mood and cognitive adverse events elucidated via a combination of pharmacovigilance data mining and functional enrichment analysis
Source: Arch Toxicol. 2020 Jun 5;94(8):2829–45. doi: 10.1007/s00204-020-02788-1 (PMC7395038; doi:10.1007/s00204-020-02788-1)
Supplement: Supplementary file 6 — Supplementary file6 (DOCX 24 kb) [file 204_2020_2788_MOESM6_ESM.docx]

Online Resource 6

**Molecular basis of mood and cognitive adverse events elucidated via a combination of pharmacovigilance data mining and functional enrichment analysis**

Christos Andronis^1,*^, João Pedro Silva^2,*^, Eftychia Lekka^1^, Vassilis Virvilis^1^, Helena Carmo^2^, Konstantina Bampali^3^, Margot Ernst^3^, Yang Hu^4^, Irena Loryan^4^, Jacques Richard^5^, Félix Carvalho^2,#^, Miroslav M. Savić^6,#^

^1^Biovista, 34 Rodopoleos Street, 16777 Athens, Greece

^2^UCIBIO, REQUIMTE, Laboratory of Toxicology, Department of Biological Sciences, Faculty of Pharmacy, University of Porto, 4050-313, Porto, Portugal

^3^Department of Molecular Neurosciences, Medical University of Vienna, Spitalgasse 4, A-1090 Vienna, Austria

^4^Translational PKPD group, Department of Pharmaceutical Biosciences, Associate member of SciLifeLab, Uppsala University, Sweden

^5^Sanofi R&D, 371 avenue Professeur Blayac, Montpellier, 34000 France

^6^Department of Pharmacology, Faculty of Pharmacy, University of Belgrade, Vojvode Stepe 450, 11000 Belgrade, Serbia

*The authors contributed equally to the manuscript.

#Corresponding authors:

Félix Carvalho, UCIBIO, REQUIMTE, Laboratory of Toxicology, Faculty of Pharmacy, University of Porto, Portugal, Tel. +351 220428600, E-mail: felixdc@ff.up.pt; Miroslav Savić, Faculty of Pharmacy, University of Belgrade, Serbia, Tel. +381 113951280, E-mail: miroslav@pharmacy.bg.ac.rs

**Supplementary Table 6** – AOPs associated with mood and/or cognitive AEs retrieved from AOPWiki and AOP-Knowledge Base. AOP IDs and titles are provided in accordance with the terminology of the referred databases.

|  | **AOP title** | **Molecular Initiating Event (MIE)** | **Adverse Outcome (AO)** | **AOP Link** |
| --- | --- | --- | --- | --- |
| **AOP ID** |  |  |  |  |
| 12 | Chronic binding of antagonist to N-methyl-D-aspartate receptors (NMDARs) during brain development leads to neurodegeneration with impairment in learning and memory in aging. | Inhibition of N-methyl-D-aspartate (NMDA) receptors | Neurodegeneration;  Impairment in learning and memory | <https://aopwiki.org/aops/12> |
| 13 | Chronic binding of antagonist to N-methyl-D-aspartate receptors (NMDARs) during brain development induces impairment of learning and memory abilities. | Inhibition of N-methyl-D-aspartate (NMDA) receptors | Neurodegeneration;  Impairment in learning and memory | <https://aopwiki.org/aops/13> |
| 17 | Binding of electrophilic chemicals to SH(thiol)-group of proteins and /or to seleno-proteins involved in protection against oxidative stress during brain development leads to impairment of learning and memory. | Binding to SH/SeH proteins involved in protection against oxidative stress | Impairment in learning and memory (during the developmental period) | <https://aopwiki.org/aops/17> |
| 42 | Inhibition of Thyroperoxidase and Subsequent Adverse Neurodevelopmental Outcomes in Mammals | Inhibition of thyroid peroxidase | Decreased cognitive function | <https://aopwiki.org/aops/42> |
| 48 | Binding of agonists to ionotropic glutamate receptors in adult brain causes excitotoxicity that mediates neuronal cell death, contributing to learning and memory impairment. | Activation of ionotropic glutamate receptors | Impairment in learning and memory | <https://aopwiki.org/aops/48> |
| 54 | Inhibition of Na+/I- symporter (NIS) leads to learning and memory impairment. | Inhibition of Na+/I- symporter (NIS) | Impairment in learning and memory | <https://aopwiki.org/aops/54> |
| 134 | Sodium Iodide Symporter (NIS) Inhibition and subsequent adverse neurodevelopmental outcomes in mammals | Inhibition of Na+/I- symporter (NIS) | Decreased cognitive function | <https://aopwiki.org/aops/134> |
| 214 | Network of SSRIs (selective serotonin reuptake inhibitors). | Activation of glucocorticoid receptors | Depression | <https://aopwiki.org/aops/214> |
| 222 | Mental stress to agitation. | Increase in glucocorticoid receptor activity | Agitation | <https://aopwiki.org/aops/222> |
| 224 | Serotonin transporter activation to depression. | Increase in serotonin transporter activity | Depression | <https://aopwiki.org/aops/224> |
| 225 | Serotonin transporter activation to agitation. | Increase in serotonin transporter activity | Agitation | <https://aopwiki.org/aops/225> |
