# Supplementary material for: Molecular basis of mood and cognitive adverse events elucidated via a combination of pharmacovigilance data mining and functional enrichment analysis
Source: Arch Toxicol. 2020 Jun 5;94(8):2829–45. doi: 10.1007/s00204-020-02788-1 (PMC7395038; doi:10.1007/s00204-020-02788-1)
Supplement: Supplementary file 7 — Supplementary file7 (DOCX 439 kb) [file 204_2020_2788_MOESM7_ESM.docx]

Online Resource 7

**Molecular basis of mood and cognitive adverse events elucidated via a combination of pharmacovigilance data mining and functional enrichment analysis**

Christos Andronis^1,*^, João Pedro Silva^2,*^, Eftychia Lekka^1^, Vassilis Virvilis^1^, Helena Carmo^2^, Konstantina Bampali^3^, Margot Ernst^3^, Yang Hu^4^, Irena Loryan^4^, Jacques Richard^5^, Félix Carvalho^2,#^, Miroslav M. Savić^6,#^

^1^Biovista, 34 Rodopoleos Street, 16777 Athens, Greece

^2^UCIBIO, REQUIMTE, Laboratory of Toxicology, Department of Biological Sciences, Faculty of Pharmacy, University of Porto, 4050-313, Porto, Portugal

^3^Department of Molecular Neurosciences, Medical University of Vienna, Spitalgasse 4, A-1090 Vienna, Austria

^4^Translational PKPD group, Department of Pharmaceutical Biosciences, Associate member of SciLifeLab, Uppsala University, Sweden

^5^Sanofi R&D, 371 avenue Professeur Blayac, Montpellier, 34000 France

^6^Department of Pharmacology, Faculty of Pharmacy, University of Belgrade, Vojvode Stepe 450, 11000 Belgrade, Serbia

*The authors contributed equally to the manuscript.

#Corresponding authors:

Félix Carvalho, UCIBIO, REQUIMTE, Laboratory of Toxicology, Faculty of Pharmacy, University of Porto, Portugal, Tel. +351 220428600, E-mail: felixdc@ff.up.pt; Miroslav Savić, Faculty of Pharmacy, University of Belgrade, Serbia, Tel. +381 113951280, E-mail: miroslav@pharmacy.bg.ac.rs


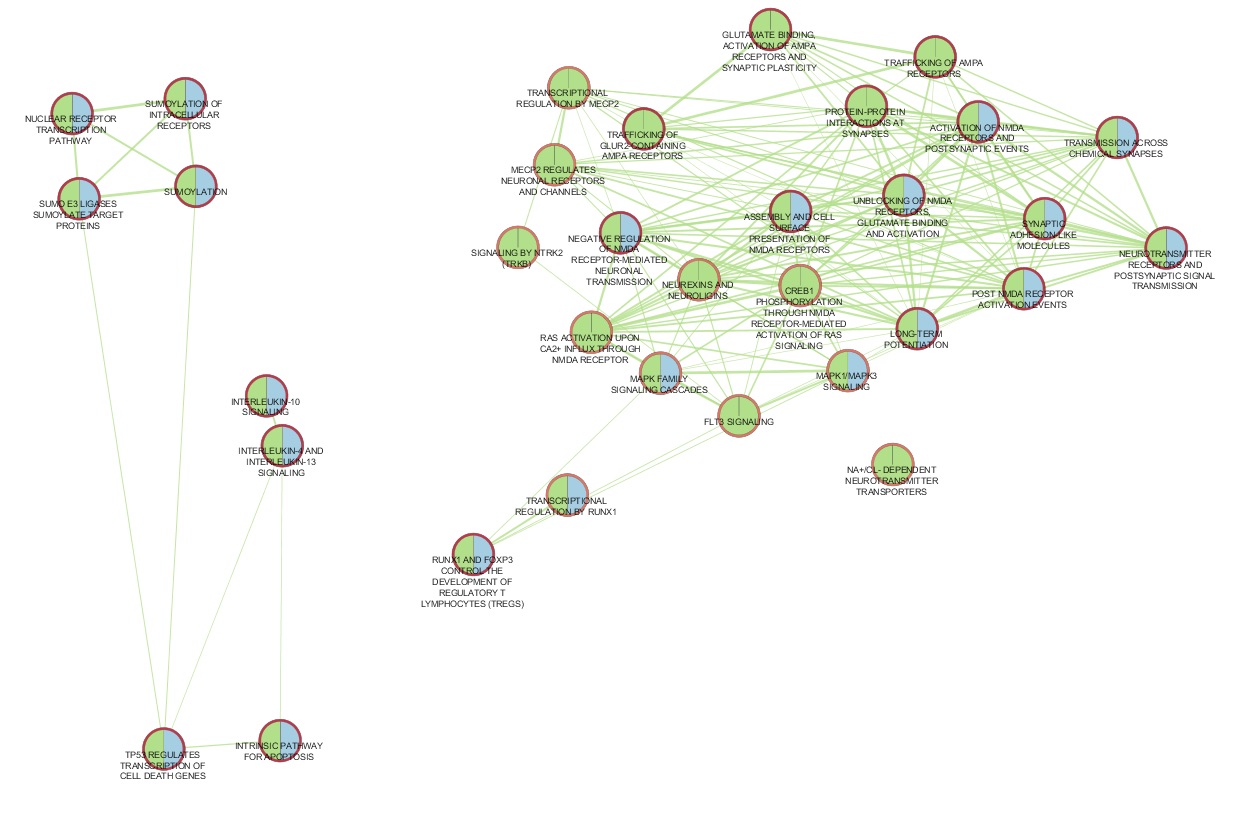


**Supplementary Figure 1 - Pathway cross-talk analysis for mood-related AEs.** The cross-talk among pathways comprising targets identified as being associated with mood- (green) was established using g:Profiler, followed by Cytoscape software analysis. Nodes common to both mood and cognitive AEs are represented in both green and blue colors.

**
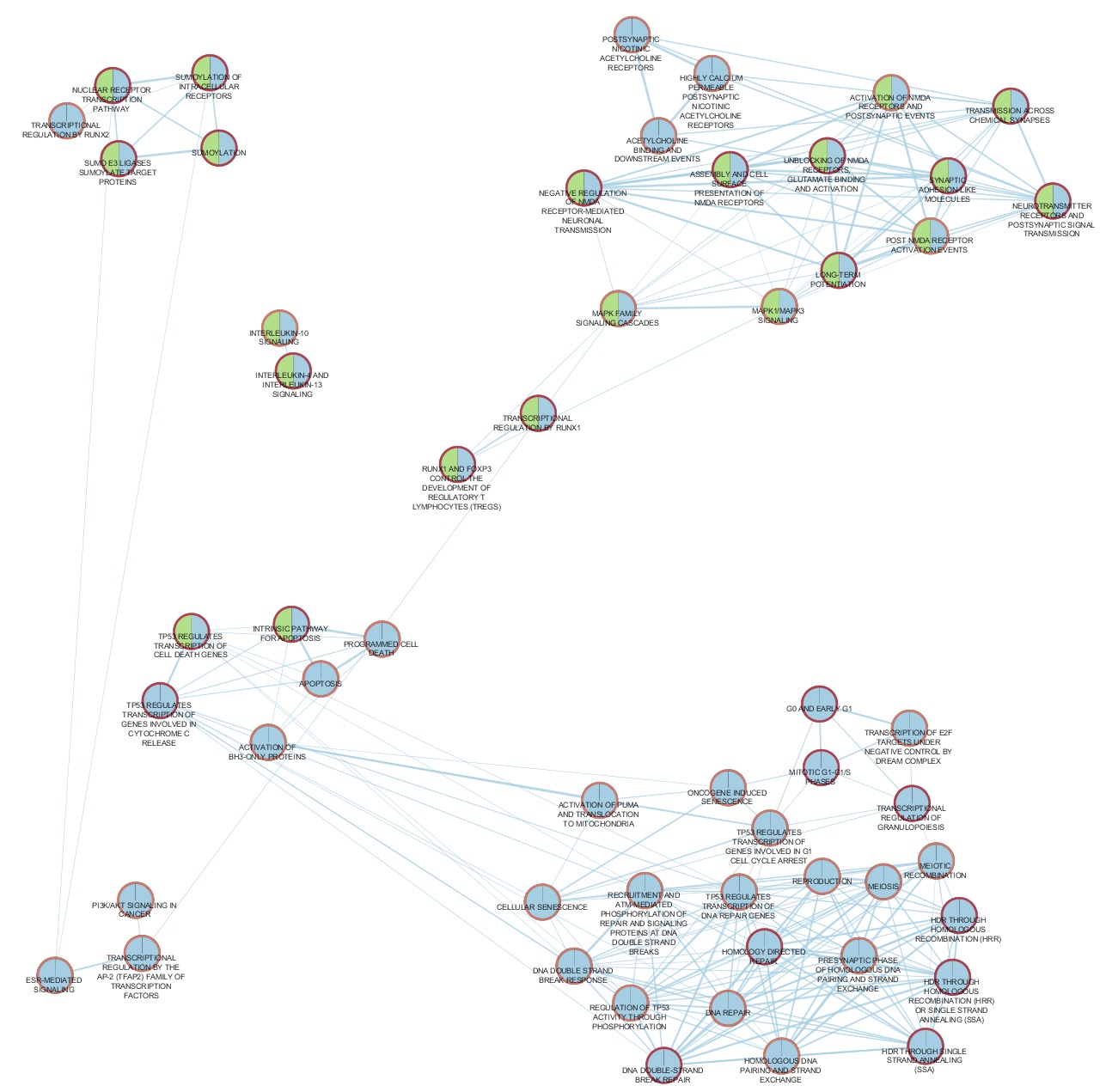
**

**Supplementary Figure 2** **- Pathway cross-talk analysis for cognition-related AEs.** The cross-talk among pathways comprising targets identified as being associated with cognition- (blue) was established using g:Profiler, followed by Cytoscape software analysis. Nodes common to both mood and cognitive AEs are represented in both green and blue colors.
